# Highly Stable Heterometallic Catalysts for Polyester Depolymerization and Polymerization at High Temperatures

**DOI:** 10.1021/jacs.5c12243

**Published:** 2025-09-29

**Authors:** Natalia V. Reis, Yali Zhou, Bige Bati, Ying Wang, Gary S. Nichol, Jennifer A. Garden, Andrew P. Dove

**Affiliations:** † School of Chemistry, 1724 University of Birmingham, Edgbaston, Birmingham B15 2TT, U.K.; ‡ EaStCHEM School of Chemistry, 3124 University of Edinburgh, Joseph Black Building, David Brewster Road, Edinburgh EH9 3FJ, Scotland, U.K.

## Abstract

Chemical depolymerization to monomer is a valuable tool in the quest to prevent the accumulation of single-use plastic, especially poly­(ethylene terephthalate), PET, in the environment. However, the protic nucleophiles and high temperatures usually required in this process make the development of rationally designed, stable catalysts very challenging. Herein, we report a series of heterobimetallic catalysts, combining Zn­(II) and Mg­(II) with K­(I) and Na­(I) to form “ate” complexes that are stable in air and at high temperatures, which can be applied in the depolymerization of PET and poly­(lactide), PLA. The catalysts present outstanding stability and remain active after 4 successive PET additions over several days on the bench. Reaction studies reveal the influence of the metal coordinated in the inner pocket of the ligand upon the activity of the metal coordinated in the outer pocket, with different heterobimetallic catalysts being found to be optimal for the depolymerization of PET and PLA respectively. Their potential for application in industrially relevant bulk polymerization of *rac*-lactide is also demonstrated and provides key insights for rational heterometallic catalyst design in the (de)­polymerization of PET and PLA.

## Graphical abstract

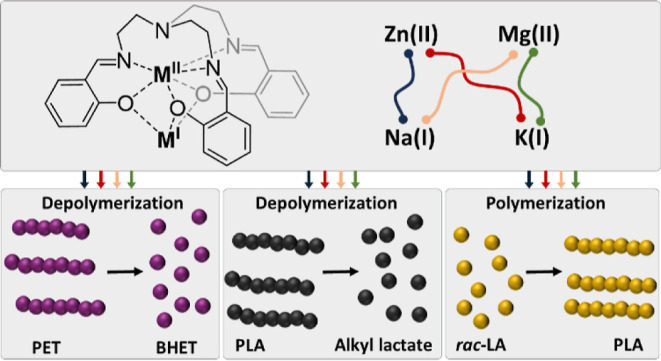

## Introduction

Catalyst design has been inspired by nature across a multitude of chemical transformations, for both small molecules and macromolecules. Multimetallic synergy, prevalent in enzymes, has particularly inspired multimetallic and heterometallic catalyst design within CO_2_ reduction^1,2^ and C–H activation,^3,4^ as well as lactide polymerization and CO_2_/epoxide copolymerization. Enzymes play key roles in degradation, displaying cooperative multimetallic effects in the breakdown of phosphomonoesters due to the proximity effect of two metal centers (Figure 1A).^5^ Beyond small molecules, enzymes have been reported for polyester depolymerization, including for polyethylene terephthalate (PET). This is a highly important target because PET is one of the most produced and used synthetic polyesters in circulation. The majority of PET (54%) is used in textiles and fibers, followed by resin for rigid containers and single-use bottles (29%—24 million metric tons (MMT)); the rest is used in films and other applications.^6,7^ Unlike depolymerization of other oxygenated polymers, such as polycarbonates to epoxide and CO_2_, the chemical recycling to monomer (CRM) of PET is widely achieved by methanolysis, hydrolysis or glycolysis, the latter using ethylene glycol to yield bis­(2-hydroxyethyl) terephthalate (BHET). As a consequence of the chemical inertness and poor solubility of PET, high temperatures (170 °C or above) and high pressure (20–40 atm) are commonly used, which requires robust catalyst systems. However, enzymes are typically unstable at high temperatures, and depolymerization is restricted to the amorphous regions of PET.

**Figure 1:**
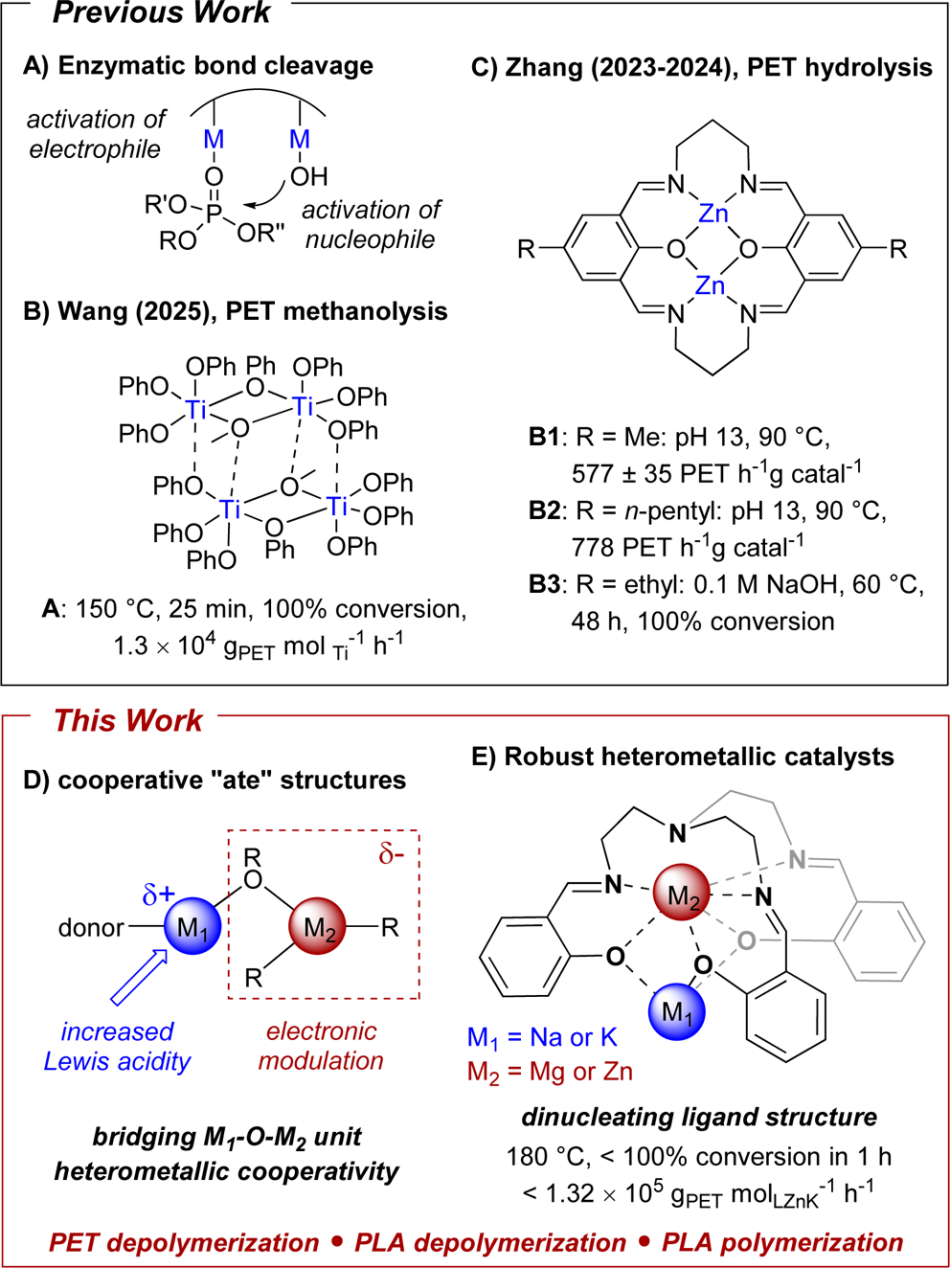
(A) Enzyme-catalyzed depolymerization of phosphomonoesters; (B) highly active multimetallic titanium cluster for PET depolymerization;^31^ (C) bimetallic catalyst for PET depolymerization based on a dinucleating ligand scaffold;^8,29,33^ (D) electronic modulation through heterometallic “ate” structures featuring a M-O-M′ bridging unit; (E) robust heterometallic catalysts for PET depolymerization based on a dinucleating TrenSal ligand scaffold.

The use of metal-based catalysts to fully depolymerize PET is an attractive alternative to enzymatic depolymerization processes. However, the harsh conditions often applied in industrially relevant PET depolymerization, specifically high temperatures and excess alcohol, also limit the potential for tailored catalyst design to improve depolymerization activity and selectivity.^8–13^ Therefore, most of the metal-based catalysts reported for PET depolymerization are simple species such as organocatalysts, metal salts (Mg or Zn Cl_2_ and (OAc)_2_) or metal-supported systems, such as Pt and Ru/Nb_2_O_5_.^5,10,14–16^

More recently, monometallic catalysts supported by an ancillary ligand have been reported, including examples based on Schiff base, pincer, guanidine, phosphine and NHC ligands (refer to Figure S2 for details).^11,17–28^ In 2021, the Jones group reported the first bimetallic catalyst for PET depolymerization.^8,12,13,29–35^ A few months ago, Wang and co-workers reported a highly active titanium phenoxide catalyst (Figure 1B),^31^ which displayed very high activity toward PET depolymerization (1.3 × 10^4^ g_PET_ mol_Ti_^–1^ h^–1^ at 150 °C), despite the lack of controlled coordination geometry at Ti due to the cluster structure. Notably, all of the multimetallic catalysts reported for PET depolymerization feature a bridging M-O-M or M-N-M unit (refer to Figure S3 for a comprehensive literature review). This electronic communication between the two metal centers can modulate the electronics at each metal center, opening up the potential for multimetallic cooperativity beyond the metal proximity effect reported for enzymes. Yet for aggregated catalysts (e.g., cluster **A**, Figure 1B), this electronic communication would be destroyed upon deaggregation to monomeric catalyst under depolymerization conditions i.e. with an excess of Lewis donors present from the polyester and alcohol. In spite of this, only one set of multimetallic catalysts reported for PET depolymerization is based on a macrocyclic ligand scaffold designed to hold two metal centers in close proximity (**B1–B3**, Figure 1C), rather than an aggregated structure.^8,29,33^ Demonstrating the influence of the bimetallic structure, catalyst **B1** outperformed Zn­(OAc)_2_, as the latter was inactive under the reaction conditions (pH 8, 40 °C). Bimetallic **B1** also depolymerized crystalline PET and was active under a wide range of reaction conditions (30–340 °C, pH 8–14).^29^

We hypothesized that cooperative heterometallic catalysts could further enhance catalyst performance in PET and PLA depolymerization. Heterometallic catalysts have outperformed their homometallic analogues across multiple fields including metal–halogen exchange,^36^ CO_2_/epoxide copolymerization,^37,38^ and the ring-opening polymerization of cyclic esters,^39,40^ which is particularly relevant as it involves similar key mechanistic steps to polyester depolymerization i.e. monomer coordination and nucleophilic attack (see Figure 1A for phosphomonoester analogue). Cooperativity can arise because the two metals perform different roles in a mechanism, and/or because one metal can influence the activity of the other by electronic modification. The latter typically occurs where the two metals are connected through a heteroatom, most typically through a M-O-M′ bridging unit (Figure 1D).^40,41^ While exploiting heterometallic cooperativity in PET depolymerization is an attractive target, there are challenges associated with designing robust catalysts stable toward the harsh conditions required for PET depolymerization under industrially relevant conditions i.e. 180 °C in excess alcohol/glycol. To tackle this challenge, we developed four heterometallic catalysts based on the heptadentate TrenSal ligand, selected as it can form highly stable metal complexes due to extensive chelation and three stable phenoxide units. Here, we show that heterometallic catalysts can depolymerize both aliphatic and aromatic polyesters, PLA and PET (Figure 1E). These complexes were stable in ethylene glycol at 180 °C and displayed different activities depending on the combination of heterometals used, highlighting the influence of the metal coordinated in the inner and outer pocket of the ligand. Moreover, activity was retained after the addition of fresh polymer batches over several days under an air atmosphere. These catalysts could also be applied in bulk polymerization of lactide, thus taking steps toward the possibility of closed-loop chemical recycling.

## Results and Discussion

The series of dinuclear catalysts were synthesized by sequential metalation of the TrenSal ligand [LH_3_], which itself was synthesized following previously reported methodology.^42^ Ligand deprotonation was carried out using the organoalkali metal reagent (KHMDS, NaHMDS or NaH) followed by reaction with divalent Mg­(HMDS)_2_ or Zn­(Et)_2_ to access the neutral bimetallic species (Figure 2 top). Single crystals suitable for X-ray diffraction were obtained from THF at −34 °C, and the molecular structures evidenced that Mg­(II) or Zn­(II) is hexacoordinated in the inner pocket of the ligand, with Na­(I) or K­(I) occupying a distorted octahedral environment in the outer pocket (Figure 2 bottom). The alkali metal impacts the aggregation state of the complex in the solid state; systems containing Na­(I) crystallized in a triclinic space group *P*1̅ and displayed monomeric structures, with the coordination number of Na­(I) satisfied by three equivalents of THF. On the other hand, the larger K­(I) favors dimerization, and complexes **LZnK** and **LMgK** crystallized in a tetragonal space group *I*4_1_/*a*.

**Figure 2:**
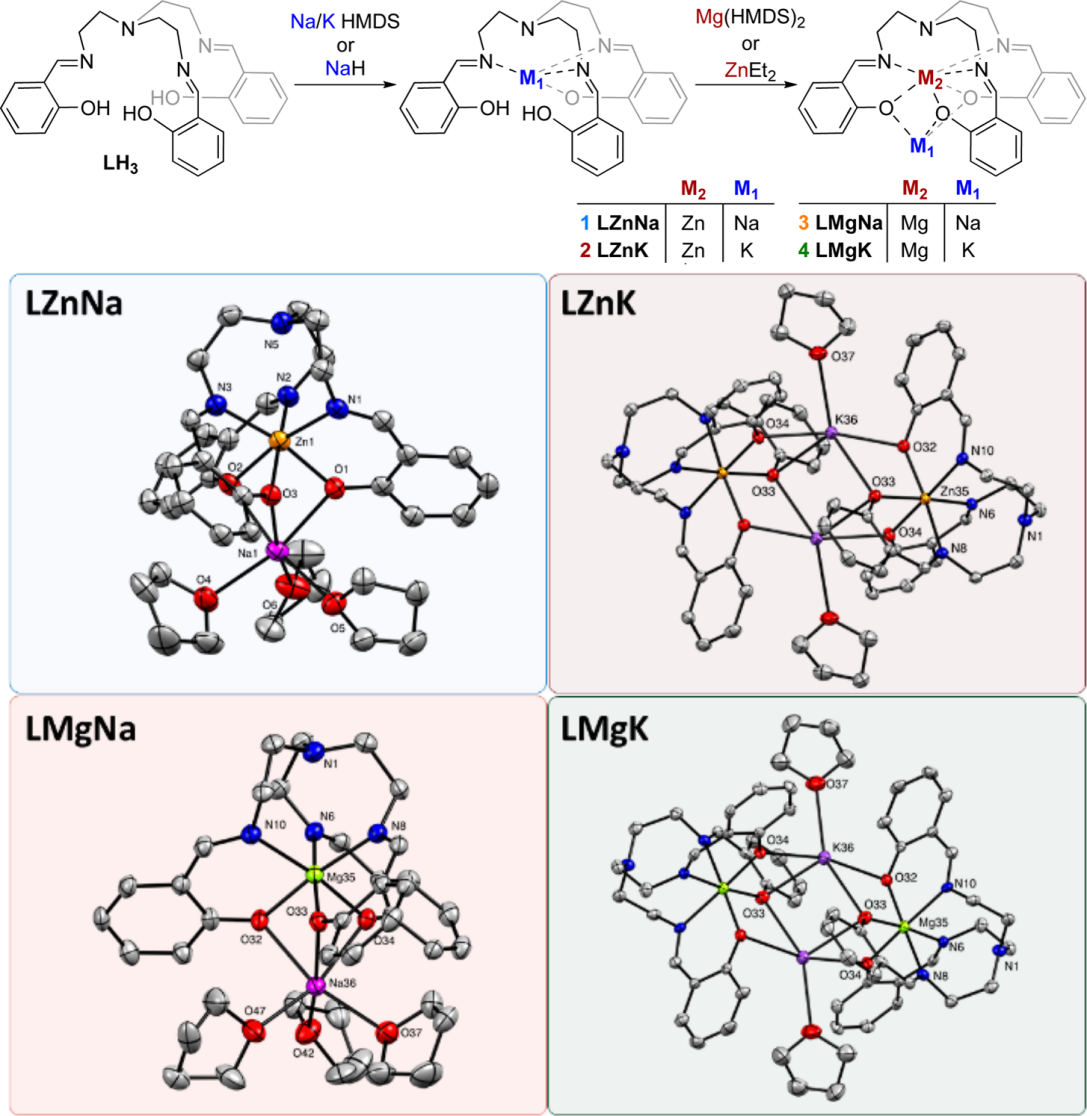
Synthesis of bimetallic complexes **LZnNa**, **LZnK**, **LMgNa** and **LMgK** with respective molecular structures.

In both cases, K­(I) is saturated by two bridging phenolic oxygen atoms from an adjacent ligand, one THF and an additional agostic interaction with an adjacent aromatic ring (K···H­(Ar) 2.965(15) Å for **2** and 2.987(4) Å for **4**), (Tables S1–S5, Figures S4–S7). In all cases, the metal–metal’ distances are within the range of 2.926–3.827 Å (Na–Zn, 2.978 Å; K–Zn, 3.795 Å and 3.827 Å; Na–Mg, 2.936 Å; K–Mg, 3.741 Å and 3.763 Å). Previous studies have shown that intermetallic proximity in the range of 3–5 Å can deliver improved catalyst performance in lactide polymerization and CO_2_/epoxide ring-opening copolymerization.^40,43,44^ which has directed ligand design toward dinucleating scaffolds.

The relative positions of the metals are likely related to different parameters such as ionic radii and electronegativity. Alkali metals (Na and K) have larger ionic radii (102 and 138 pm, respectively) when compared to Zn and Mg, and therefore occupy the more flexible outer pocket of the ligand. In this series, metals with higher electronegativity (Zn > Mg > Na > K) preferentially coordinate within the inner pocket (N_3_O_3_), which contains more Lewis donors than the outer pocket (O_3_). This trend differs from previously reported LNaAlMe, where Na­(I) occupied the inner pocket and Al­(III) was positioned in the outer pocket. In this case, the presence of the Al-methyl group forced Al­(III) to occupy the larger pocket, and the high oxophilicity of Al may also be a contributing factor.^45^ Although the complexes crystallized as monomeric or dimeric structures, diffusion-ordered spectroscopy (DOSY) NMR analysis indicated that all complexes are monomeric in *d*_8_-THF, after comparing the diffusion obtained in each system to a calibration curve (Table S6).

While the complex synthesis was carried out under an inert atmosphere, the use of excess protic reagents (i.e., ethylene glycol) and high temperatures under depolymerization conditions led us to investigate the stability of the complexes toward heat and alcohols. The stability of the catalyst at the temperature of the reaction was confirmed by TGA analysis of each complex. No decomposition was observed after the system was kept at 180 °C for 3 h, apart from the loss of the coordinated THF; this was confirmed by ^1^H NMR spectroscopic analysis of the complexes before and after thermal treatment (Figures S32–S35). Next, **LZnNa** or **LMgNa** was combined with BnOH in a 1:1 ratio in toluene at 120 °C and analyzed by NMR spectroscopy (Figures S40 and S41). No significant chemical shift was observed in the signals that correspond to the catalysts, and the only difference in the BnOH resonances was a change in the splitting of the benzylic–CH_2_ resonance from a doublet to a singlet, indicating a weak interaction or H-bonding with the catalyst. Providing further support for the catalyst stability toward BnOH, DOSY NMR spectroscopic analysis gave different diffusion coefficients for **LZnNa** and BnOH, showing no reaction between them (Figure S38). Finally, heterometallic **LZnK** was investigated in the presence of 18-crown-6 ether (2 equiv). Crown ethers excel at extracting alkali metal cations, and 18-crown-6 is perfectly size matched for K^+^. Two sets of single crystals suitable for XRD were obtained from THF and toluene respectively, which showed that the heterometallic structures persisted even in the presence of crown ethers and excess Lewis donors (Figures S8 and S9).

The activity of the complexes toward PET depolymerization was investigated using benchtop ethylene glycol, without purification or drying, in air. The reaction was performed using 0.3 mm PET powder with ethylene glycol at 180 °C with 0.01 equiv of catalyst; NMP was added as an internal standard. All systems were active for the glycolysis of PET accessing full conversion in less than 40 min (Figure 3). The high level of chelation and steric hindrance around the divalent metal coordinated in the inner pocket of the ligand likely makes it inaccessible to the substrates, however, it still plays an important role in the activity of the catalyst. The catalyst activity of both the Na­(I) and K­(I) complexes increased when the inner pocket metal was changed from Mg­(II) to a more electronegative Zn­(II). The ligand framework allows electronic communication between the two metal centers to form “ate” complexes, with the presence of the divalent metal thus enhancing the Lewis acidity of the alkali metals;^46–48^ **LZnK** was the most active catalyst of those tested, depolymerizing 1.32 × 10^5^ g_PET_ mol_**LZnK**_^–1^ h^–1^ at 180 °C (ESI Table S7, Entry 2), thus bringing this into a comparable activity to the highly active titanium cluster **C** (1.3 × 10^4^ g_PET_ mol_Ti_^–1^ h^–1^ at 150 °C).

**Figure 3:**
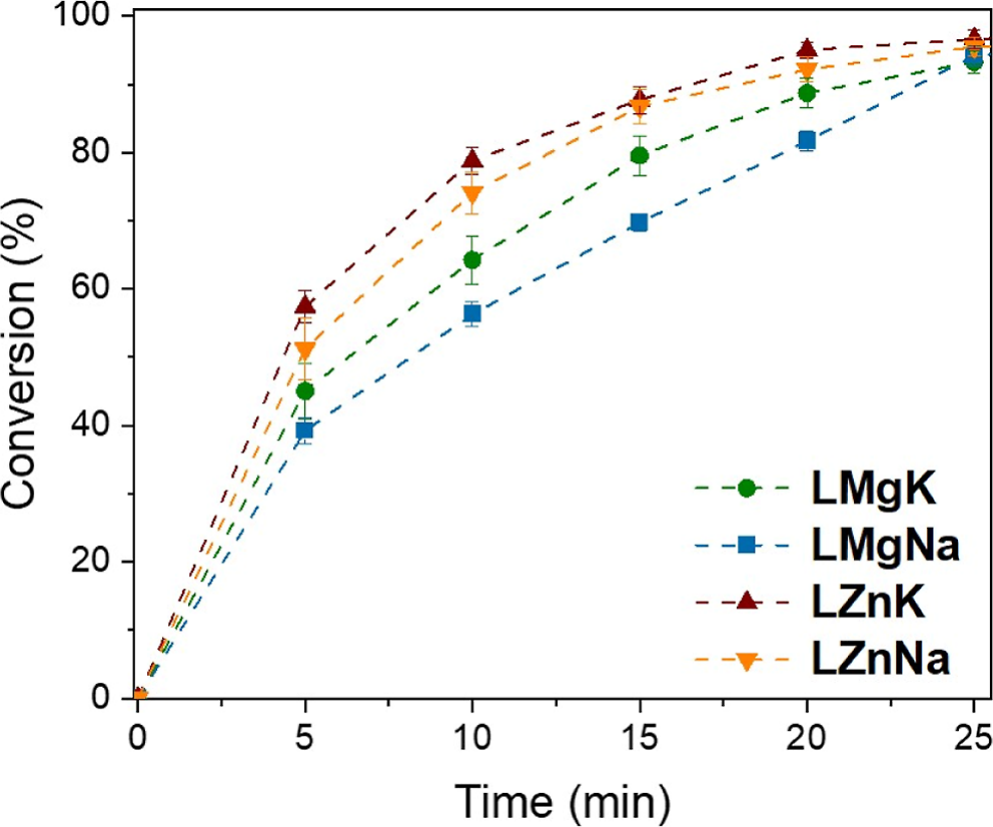
Plot of conversion *vs* time for the heterogeneous depolymerization of PET with ethylene glycol at 180 °C using catalysts **LMgK**, **LMgNa**, **LZnK** and **LZnNa** (see Table S7 for additional details).

The influence of the metal coordinated in the inner pocket of the ligand was evident when comparing depolymerization using complexes **LZnNa** and **LMgNa** with the homometallic TrenSal complex **LNa**_**3**_ (Figure 4), synthesized following previously reported procedures.^49^ When only Na­(I) was coordinated in the ligand structure, the system remained active but depolymerization was slower. The reaction rate was improved when Mg­(II) was introduced into the inner pocket (**LMgNa**), and improved even further when Zn­(II) was coordinated in the inner pocket instead, clearly demonstrating how the inner metal can activate the external metal and enhance depolymerization. PET depolymerization using the **LMgNa** system was also compared with the monometallic **L**_**2**_ **Mg**_**3**_ **·6H**_**2**_ **O** analogue, which was synthesized following literature procedures,^50^ and a mixture of 1:1 **L**_**2**_ **Mg**_**3**_ **·6H**_**2**_ **O** and **LNa**_**3**_. In this case, heterometallic **LMgNa** outperformed the homometallic mixture, demonstrating the importance of the coordination of both metal centers to the same ligand structure (Figure S29).

**Figure 4:**
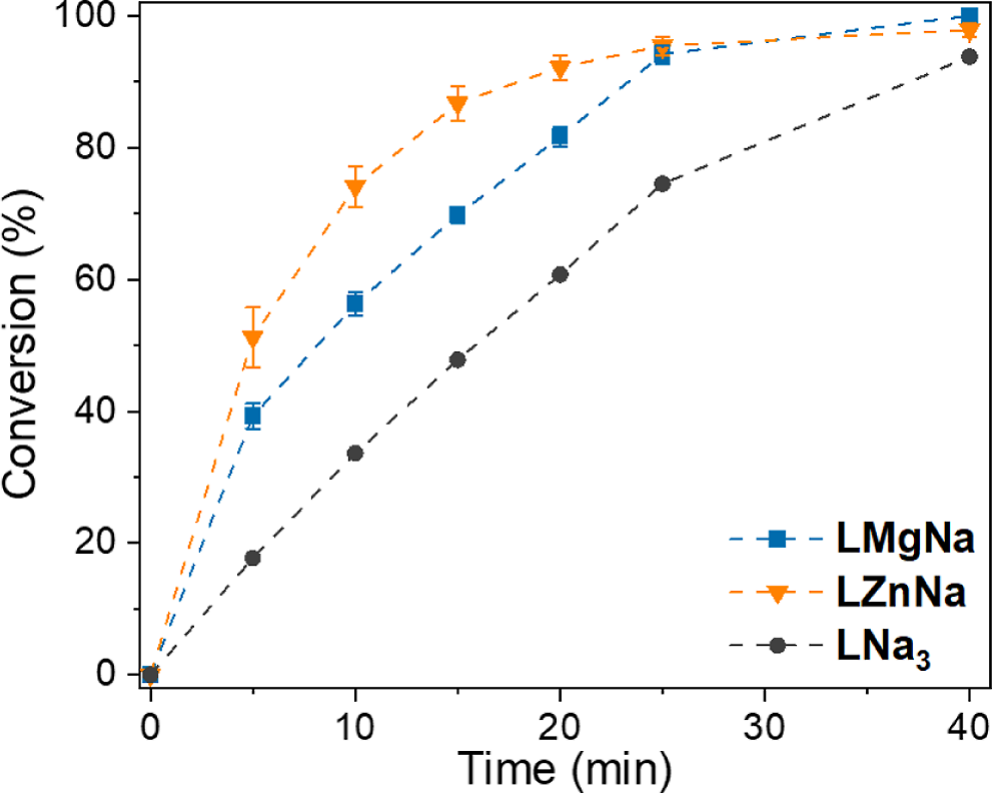
Comparison of conversion *vs* time plots for the heterogeneous depolymerization of PET with ethylene glycol at 180 °C using catalysts **LMgNa**, **LZnNa** and monometallic **LNa**_**3**_.

To further explore the utility of these catalysts, PET depolymerization was investigated at different temperatures using **LZnK** and **LMgK**, as the K-containing complexes consistently outperformed the Na analogues. Therefore, **LZnK** and **LMgK** were tested under demanding conditions and using real-world waste plastic, to push the limits of these catalyst systems. Both **LZnK** and **LMgK** remained active, albeit slower, at a lower temperature range (150 and 120 °C), with full conversion after 40 h at 150 °C (Figures S23 and S24). At all three temperatures, **LZnK** outperformed **LMgK** (Figure S23), and the activity enhancement with **LZnK** is especially notable at 150 °C (Figure S24). Longer reaction times were also required when PET pellets were used as the source at 180 °C (Figure S25); approximately 9 days were required for complete depolymerization with **LMgK** indicating that, in line with other studies, depolymerization rate is directly related to both reaction temperature and polymer surface area.^51–53^

To confirm that the catalyst remained stable under the depolymerization conditions, a reaction mixture containing **LMgK** or **LZnK**, ethylene glycol and NMP (as internal standard) was heated at 180 °C for 5 h before PET was added. In both cases the catalyst remained active with little variation in the rate of polymerization compared to the original system, where the reaction mixture was only heated for 5 min to ensure the reaction mixture was at the desired temperature prior to PET addition (Figures S26 and S27). Exploiting this stability, we sought to apply the catalysts over several depolymerization cycles. Using **LMgK**, subsequent batches of powdered PET were added to the depolymerization after the initial reaction was completed, without removal of the BHET product. The system remained highly active, albeit with slightly reduced activity, and the new PET was completely consumed in 1 h (Figure 5). The system was subsequently cooled to room temperature and held for 24 h on the bench. After a day, the system was heated at 180 °C for 5 min and a third batch of PET was added to the same system, again without BHET removal. Gratifyingly, the system remained active and again depolymerized all of the newly added PET in less than 1 h. The same procedure was repeated leaving the reaction mixture at room temperature for 7 and 25 days before it was heated at 180 °C for 5 min and a fresh batch of PET was added. In all cases, the system was active with the depolymerization going to completion after approximately 2 h. It is important to note that between the PET additions, the system was kept under air. The **LZnK** catalyst showed similar, albeit slightly lower stability (refer to Figure S28 for details). Despite the reduced activity for runs 4–5, both catalysts showed remarkable stability and the potential to be used on a larger scale with the possibility to be easily stored and transported.

**Figure 5:**
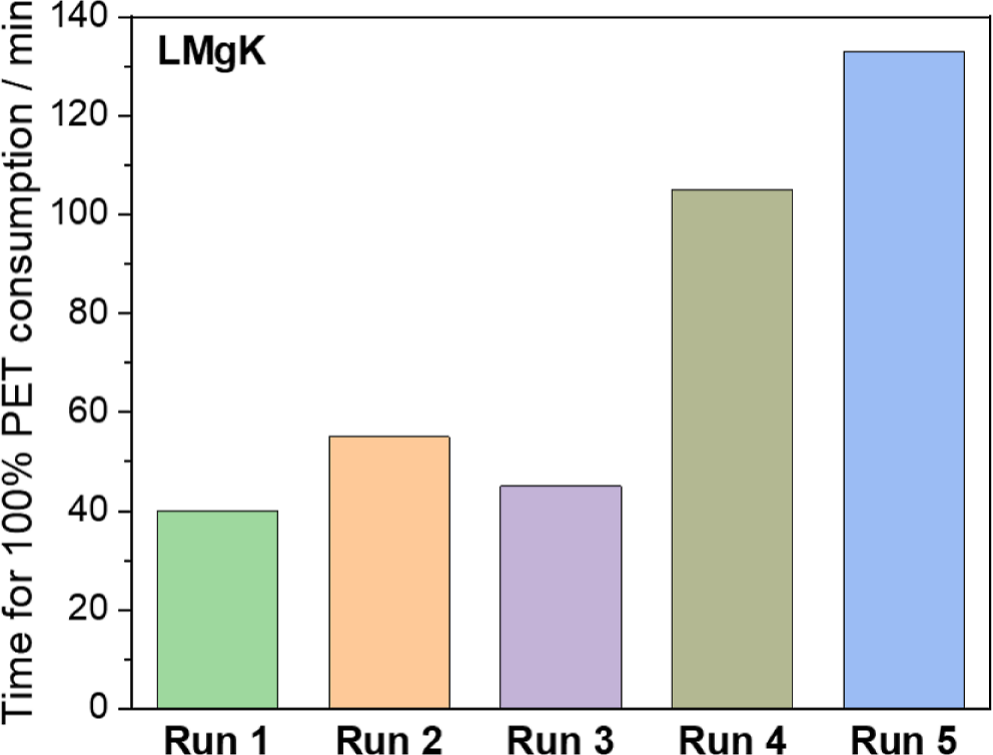
Depolymerization of successive additions of PET using **LMgK**. After complete depolymerization, the system was left to cool down to room temperature for different periods of time prior to restarting the reaction via heating to 180 °C for 5 min and adding a new batch of PET (Run 2: immediate addition; Run 3: 24 h; Run 4: 7 days and Run 5: 25 days).

Finally, **LMgK** and **LZnK** were applied to a postconsumer waste PET bottle (Figures S30 and S31). In both cases, the catalysts mediated the depolymerization of the bottle in ≤2 h, with a straightforward recovery of BHET from precipitation in water. This suggests that both **LMgK** and **LZnK** can tolerate additives present in commercial PET bottles, with **LZnK** displaying higher activities. In both cases, the depolymerization of PET powder was faster than that of the PET bottle, especially in the initial stages of the reaction (Figures S30 and S31), which is attributed to the higher surface area of the PET powder.

Encouraged by the different activities obtained based on the catalyst design during PET depolymerization, and the potential to expand the applicability of our system, the same set of catalysts was applied in the depolymerization of PLA. All systems were active, with full conversion in as little as 14 min (Figure 6). Interestingly, the order of activity was different when compared to depolymerization of PET, showing the potential of heterobimetallic catalysts for modulating activity and selectivity in depolymerization. While the same pattern was generally observed with the Zn­(II) complexes outperforming the Mg­(II) analogues, the overall order of reactivity changed, and the combination of a smaller ionic radius Na­(I) with Zn­(II) or Mg­(II) mostly surpassed the depolymerization activity of systems with K­(I). The enhanced performance of catalysts containing K­(I) for PET depolymerization can be correlated to the enhanced ability of K­(I) to form metal-π interactions with the polymer backbone compared to Na­(I).^4,54,55^ Overall, the **LZnNa**, **LMgNa** and **LZnK** catalysts showed similar activity for PLA depolymerization, with **LMgK** as the least active. Therefore, the order of depolymerization rates follow the trends:

PET: ZnK > ZnNa > MgK > MgNa.
PLA: ZnNa > MgNa ∼ ZnK > MgK.

**Figure 6:**
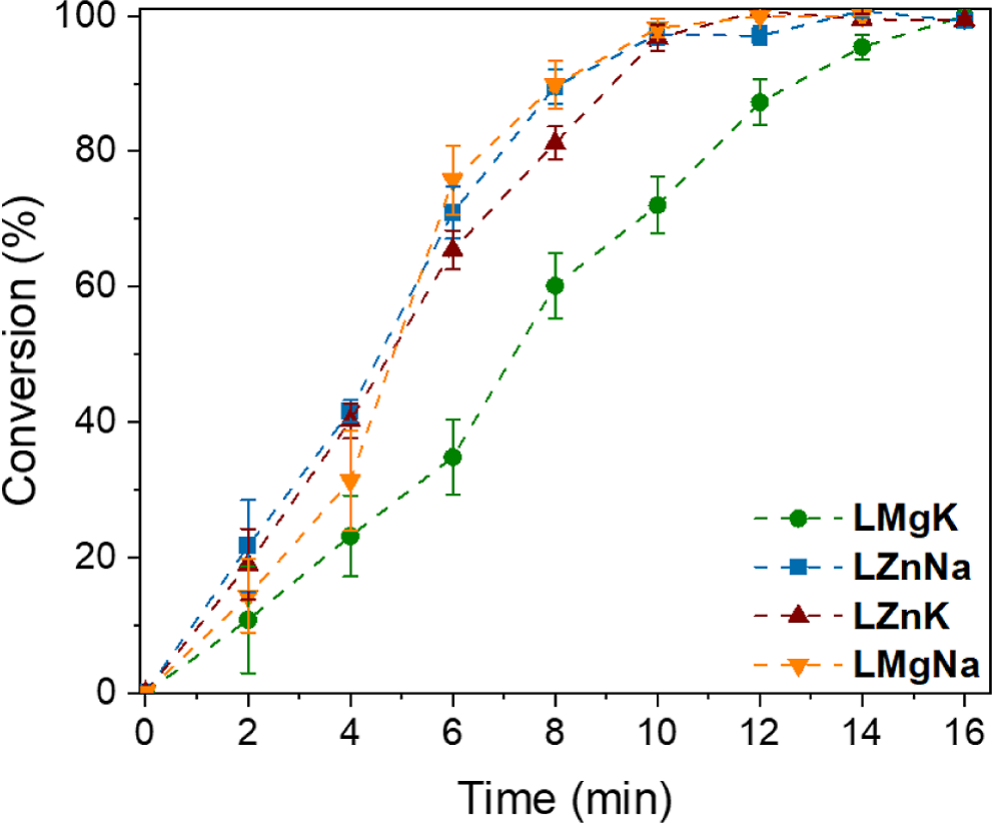
Depolymerization of PLA using catalysts **LZnNa**, **LZnK**, **LMgNa** and **LMgK**.

These results evidence how the metal combination influences the activity of each catalyst toward different polymer systems, opening a new area of research where a rational design of catalyst, from ligand structure to metal centers, can be explored to improve activity and selectivity toward plastic depolymerization.

To study the possibility of creating a closed loop system, the polymerization of lactide, LA, was investigated using **LZnNa**, **LZnK**, **LMgNa** and **LMgK**. The stability of the catalysts to high temperature and protic impurities suggested that bulk polymerization would be possible. However, we initially focused on understanding the *rac*-LA polymerization kinetics of the catalyst family in toluene and THF, using BnOH as an initiator (Figures 7 and 8), on account of the practical challenges of performing such studies in bulk. While control reactions showed that all four complexes were active in the absence of BnOH (Table S8), the activities were significantly higher when BnOH was present. Benzoxide end-capped polymers were obtained by MALDI-ToF (refer to Supporting Information for details), and the SEC data generally gave good agreement with one polymer chain per BnOH initiator (Table S8). Taken together with the catalyst:BnOH reactivity studies (vide supra), this suggests that an activated monomer mechanism occurs, as previously reported for metal complexes featuring strong metal-alkoxide bonds such as TrenSal complexes (Figure S42).^56–59^

**Figure 7:**
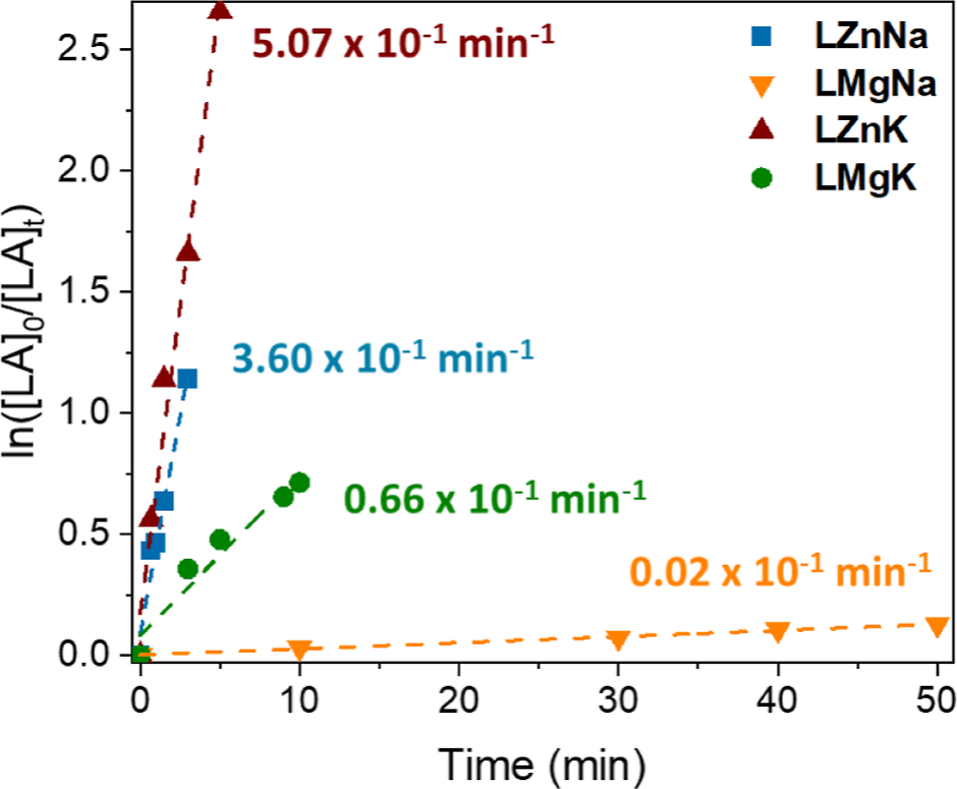
Polymerization of *rac*-LA for all catalysts **LZnNa**, **LZnK**, **LMgNa** and **LMgK** performed in toluene.

**Figure 8:**
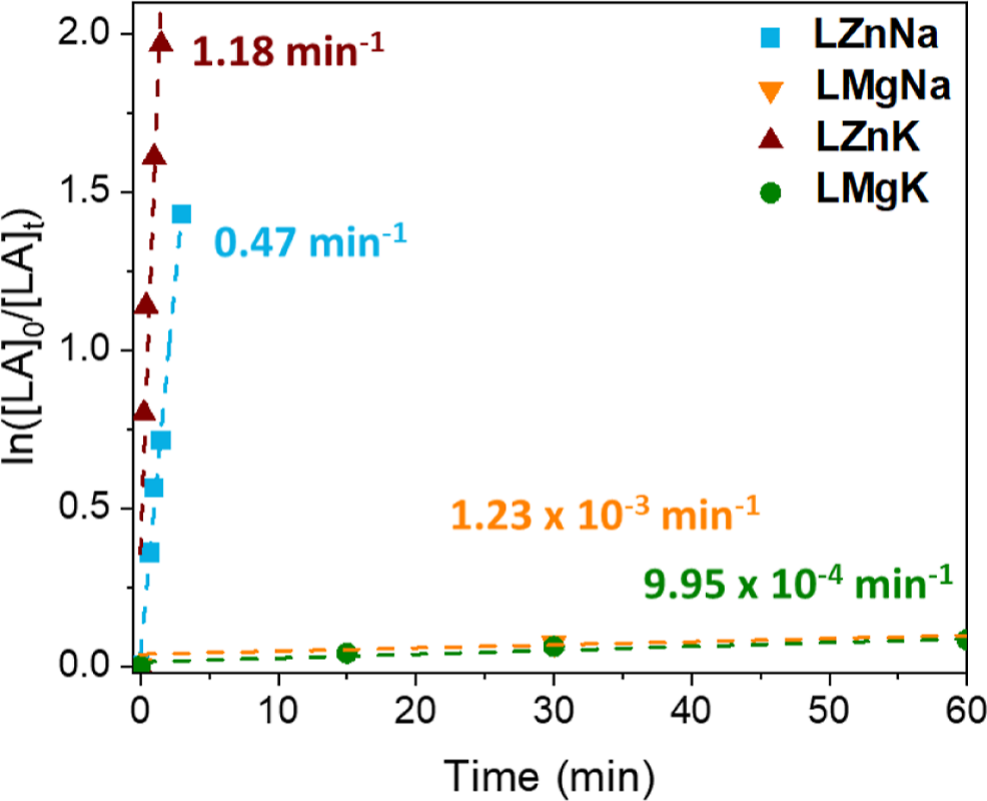
Polymerization of *rac*-LA for all catalysts **LZnNa**, **LZnK**, **LMgNa** and **LMgK** performed in THF.

Kinetic analysis showed the same trend for *rac*-LA polymerization as for PET depolymerization (ZnK > ZnNa > MgK > MgNa, Figures 7 and 8 and Tables S9 and S10), and hence a different order of activity from the depolymerization of PLA. Specifically, the K­(I)-based complexes generally gave higher polymerization activities than their Na­(I) analogues, with higher rates obtained when Zn­(II) was coordinated in the inner pocket. While it is important to note that the Mg-based catalysts were less soluble than the Zn-based systems, the consistent trend of the Zn­(II) complexes outperforming their Mg­(II) analogues in *rac*-LA polymerization, PLA depolymerization and PET depolymerization suggests that the formation of “ate” complexes is important. “Ate” complexes, such as sodium magnesiate (NaMgR_3_) or potassium zincate (KZnR_3_) species can enhance the Lewis acidity of the Na^+^ or K^+^ cation, facilitating coordination (thus activation) of Lewis donors such as cyclic ester monomers or ester groups in a polyester. As a consequence of the higher electronegativity of Zn vs Mg, there may be an increased “ate” character in **LNaZn** and **LKZn** compared to the Mg­(II) counterparts. The enhanced activities of the K­(I)-based complexes over Na­(I) are attributed to the larger ionic radius of K, which has previously been reported to facilitate *rac*-LA coordination thus activation toward nucleophilic attack and ring-opening.^48^ Notably, the most active catalyst (**LZnK**) displayed excellent activities giving full conversion of 100 equiv. *rac*-LA in just 5 min (THF, 60 °C with 1 equiv. BnOH). On the other hand, the SEC data generally shows that the Mg­(II) complexes gave better polymerization control compared to the Zn­(II) counterparts in both THF (e.g., **LZnNa/K**, *D̵*_M_ = 1.38–1.71, Table S9; **LMgNa/K**, *D̵*_M_ = 1.07–1.74, Table S10) and toluene (e.g., **LZnNa/K**, *D̵*_M_ = 1.51–2.10, Table S9; **LMgNa/K**, *D̵_M_* = 1.05–2.31, Table S10).

The influence of the metal in the “inner” pocket was confirmed by attempts to polymerize *rac*-LA using the mono-Na (**LH**_**2**_ **Na**) and mono-K (**LH**_**2**_ **K**) versions of the catalyst, in THF and toluene. The monometallic catalysts were highly active (Table S11). While **LH**_**2**_ **K** outperformed **LH**_**2**_ **Na** in terms of activity, converting 89 equiv of LA in 1 min in THF at 60 °C, the dispersities were broad (*D̵*_M_ = 2.11).^49^ Compared to the monoalkali metal analogues, the heterometallic complexes containing Zn­(II) in the inner pocket mostly presented better activity and polymerization control with narrower *D̵*_M_ values (Table S11). For example, **LZnNa** converted 51 equiv of LA in 1.5 min in THF (*D̵*_M_ = 1.40), whereas **LH**_**2**_ **Na** converted 29 equiv under identical conditions (*D̵*_M_ = 1.47). In contrast, the presence of Mg­(II) in the inner pocket slowed the polymerization process compared to the mono-Na and K but increased the polymerization control (Table S11). Overall, these findings provide strong evidence for the importance of the influence of the inside metal on the behavior of the outside one, in both polymerization and depolymerization processes (Figures 9 and S42).

**Figure 9:**
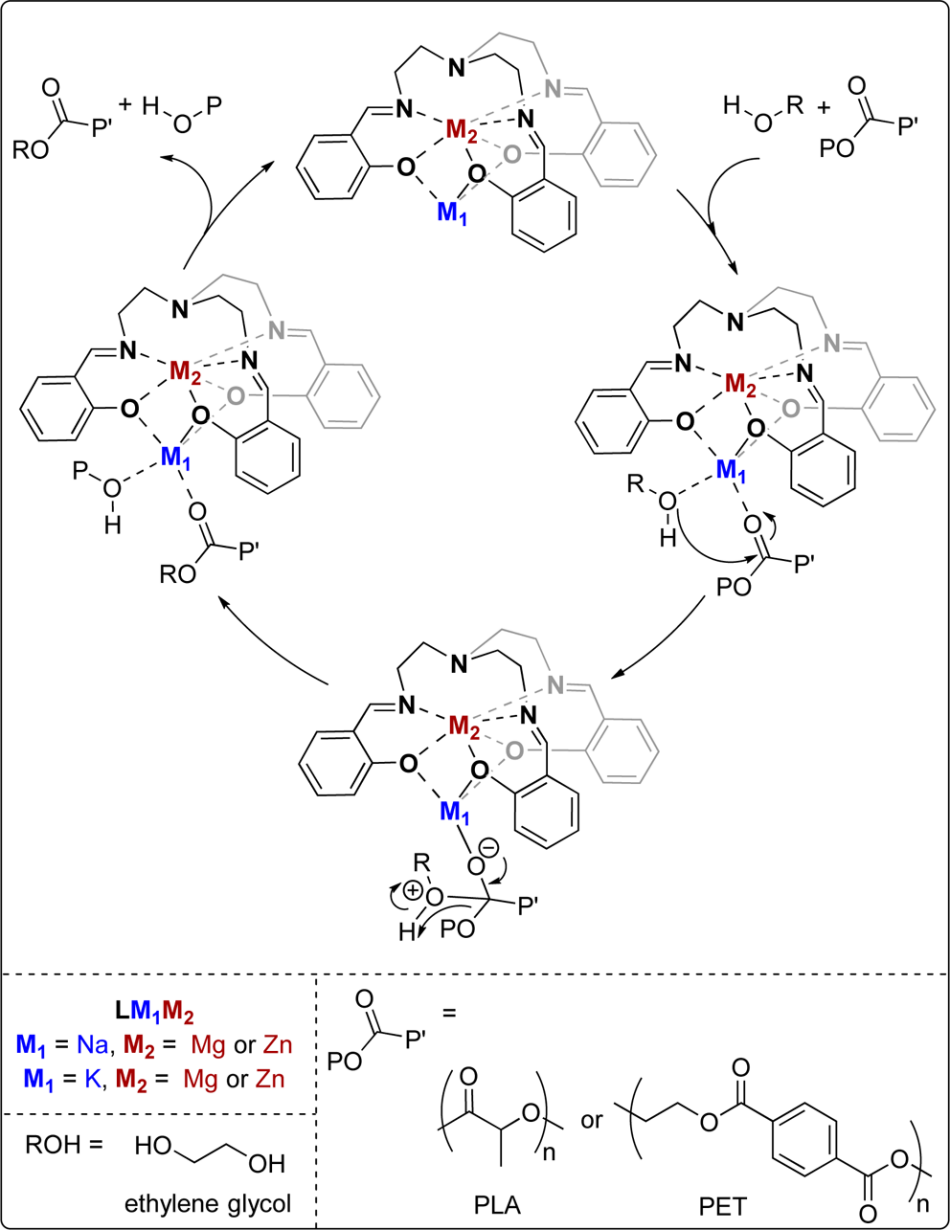
Proposed mechanism for the depolymerization of poly­(lactic acid) or poly­(ethylene terephthalate) by catalysts **LZnNa**, **LZnK**, **LMgNa** and **LMgK**, in the presence of excess ethylene glycol.

Finally, **LMgK** was applied for the polymerization of l-LA in bulk at 180 °C, to mimic industrially relevant conditions. The system was reacted for 20 min and then quenched, with conversion reaching 95%. These findings are highly promising as they expand the catalyst design to extreme reaction conditions for polymerization as well as depolymerization. It also highlights the importance of the heterometallic indirect synergic effect where one metal center helps activate the other metal, increasing the reaction rate without participating directly in the polymer/monomer or alcohol coordination, which are key steps in both the polymerization and depolymerization of polyesters.

## Conclusions

We have reported the first example of applying heterobimetallic catalysis in the depolymerization of PET and PLA. The four new heterometallic catalyst species reported combine alkali metals with divalent metals and, in contrast to many typical metallo-organic catalyst species, were all surprisingly stable in air atmosphere, at high temperature in the presence of protic solvents and impurities. This stability enabled their study as catalysts for depolymerization and polymerization reactions: PET and PLA depolymerization as well as *rac*-LA bulk polymerization, under industry relevant conditions. Important synergic effects between the two metals demonstrate that even when the metal coordinated in the inner pocket of the TrenSal ligand was not accessible for reaction, it played an important role in dictating the catalytic performance of the exposed metal center in the outer pocket. Most notably, these studies demonstrate that even for similar transformations—namely PET and PLA depolymerization—subtle differences in catalytic trends were evident, underlining the concept that rational ligand and catalyst design in heterobimetallic systems can be extended to depolymerization reactions. This demonstrates differences between the ideal heterocombinations for aliphatic and aromatic polyesters, which may be due to the enhanced propensity for K to form metal-π interactions, bringing the PET backbone into proximity with the active metal center to catalyze alcoholysis. The high stability has enabled these catalysts to demonstrate recyclability, retaining activity when stored under air, as well as utility in other demanding reactions, exemplified here by the bulk polymerization of *rac*-lactide. Beyond the immediate demonstration in polymerization/depolymerization, these findings highlight the potential to harness heterometallic synergy for rational catalyst design in demanding reaction environments.

## Supplementary Material



## Notes

All synthetic procedures, instrumentation, characterization and additional data (NMR spectra, crystallographic data, MALDI-ToF and TGA) (PDF).

## Abbreviations

PET: polyethylene terephthalate
LA: lactide
PLA: poly­(lactic acid)
TGA: thermogravimetric analysis
DOSY: diffusion ordered spectroscopy
HMDS: bis­(trimethylsilyl)­amide
NMP: *N*-methyl-2-pyrrolidone
TrenSal: 2,2′,2″-tris­(salicylideneimino) triethylamine.
