# Supplementary material for: Highly Stable Heterometallic Catalysts for Polyester Depolymerization and Polymerization at High Temperatures
Source: J Am Chem Soc. 2025 Sep 29;147(40):36697–705. doi: 10.1021/jacs.5c12243 (PMC12512191; doi:10.1021/jacs.5c12243)
Supplement: Supplementary file 1 [file ja5c12243_si_001.pdf]

Supporting Information

Highly stable heterometallic catalysts for polyester depolymerization and polymerization at high temperatures

Natalia V. Reis[a], Yali Zhou[b], Bige Bati[a], Ying Wang[b], Gary S. Nichol[b], Jennifer A. Garden[b]*, Andrew P. Dove[a]*

[a] School of Chemistry, University of Birmingham, Edgbaston, Birmingham B15 2TT, UK

[b] EaStCHEM School of Chemistry, University of Edinburgh, Joseph Black Building, David Brewster Road, Edinburgh, Scotland, UK

Contents

Materials 4

Characterization and Instrumentation 4

Experimental Details 6

Synthesis and characterization of TrenSal complexes **LZnNa, LZnK, LMgNa**, **LMgK** and **LH$_2$K** 6

Depolymerization of PET powder 7

Figure S1. Experimental set-up for PET depolymerization, highlighting the heterogeneous nature. 8

Polymerization of rac-LA in THF or toluene solvent 8

Supplementary Figures and Tables 9

Figure S2. Key ligand classes of monometallic catalysts featuring ancillary ligands for PLA and PET depolymerization.[7–19] 9

Figure S3. Comprehensive literature examples of homobimetallic and homomultimetallic catalysts for PLA and PET depolymerization.[20–30] 10

Crystallographic Data 11

Table S1. Crystallographic data and refinement detail for complexes **LZnNa**, **LMgNa** and **LMgK**. 11

Table S2. Crystallographic data and refinement detail for complexes **LZnK**, **[LZnK]$_\infty$** and **[K(THF)$_2$(18-c-6)]$^+$[K{LZn}$_2$]$^-$** 12

Table S3. Selected bond lengths (Å) for complexes **LZnNa**, **LMgNa** and **LMgK**. 12

Table S4. Selected bond lengths (Å) for complexes **LZnK, [LZnK]$_\infty$** and **[K(THF)$_2$(18-c-6)]$^+$[K{LZn}$_2$]$^-$**. 13

Table S5. Selected bond angles (°) for complexes **LZnNa**, **LMgNa**, **LZnK**, **LMgK, [LZnK]$_\infty$** and **[K(THF)$_2$(18-c-6)]$^+$[K{LZn}$_2$]$^-$** 13

Figure S4. Molecular structure of complex **LZnNa**·3THF crystallized from THF solvent with displacement ellipsoids at the 50% probability level. Hydrogen atoms, solvents and disorder in the THF are omitted for clarity. 16

Figure S5. Molecular structure of complex [**LZn$_2$K**]$_2$·2THF crystallized from THF solvent with displacement ellipsoids at the 50% probability level. Hydrogen atoms and solvents are omitted for clarity. 16

Figure S6. Molecular structure of complex **LMgNa**·3THF crystallized from THF solvent with displacement ellipsoids at the 50% probability level. Hydrogen atoms, solvents and disorder in the THF are omitted for clarity. 17

Figure S7. Molecular structure of complex [**LMgK**]$_2$·2THF crystallized from THF solvent with displacement ellipsoids at the 50% probability level. Hydrogen atoms, solvents and disorder in the THF are omitted for clarity. 17

Figure S8. Molecular structure of complex **LZnK** crystallized from THF in the presence of 2 equivalents of 18-crown-6 ether shown with chemically correct proportions, denoted as **[K(THF)$_2$(18-c-6)]$^+$[K{LZn}$_2$]$^-$** with displacement ellipsoids at the 50% probability level. Hydrogen atoms and solvents are omitted for clarity. 18

Figure S9. Molecular structure of the unit cell (top) and the polymeric structure (bottom) of complex **LZnK** crystallized from toluene in the presence of 2 equivalents of 18-crown-6 ether, denoted **[LZnK]$_\infty$**, with displacement ellipsoids at the 50% probability level. Hydrogen atoms and solvents are omitted for clarity. 18

^{1}H and ^{13}C NMR Spectra 19

Figure S10. ^{1}H NMR spectrum of **LZnNa** (THF-d$_8$, 500 MHz, 298 K). 19

Figure S11. ^{13}C NMR spectrum of **LZnNa** (THF-d$_8$, 126 MHz, 298 K). 19

Figure S12. ^{1}H NMR spectrum of **LZnK** (THF-d$_8$, 500 MHz, 298 K). 20

Figure S13. ^{13}C NMR spectrum of **LZnK** (THF-d$_8$, 126 MHz, 298 K). 20

Figure S14. ^{1}H NMR spectrum of **LMgNa** (THF-d$_8$, 500 MHz, 298 K). 21

Figure S15. ^{13}C NMR spectrum of **LMgNa** (THF-d$_8$, 126 MHz, 298 K). 21

Figure S16. ^{1}H NMR spectrum of **LMgK** (THF-d$_8$, 500 MHz, 298 K). 22

Figure S17. ^{13}C NMR spectrum of **LMgK** (THF-d$_8$, 126 MHz, 298 K). 22

Figure S18. ^{1}H NMR spectrum of **LH$_2$K** (DMSO-d$_6$, 500 MHz, 298 K). 23

Figure S19. ^{13}C NMR spectrum of **LH$_2$K** (DMSO-d$_6$, 126 MHz, 298 K). 23

Table S6. Diffusion coefficients and predicted molecular weights for complexes **LZnNa**, **LZnK**, **LMgNa**, **LMgK**, **LH$_2$Na** and **LH$_2$K** (THF-d$_8$, 500 MHz, 298 K).[a] 24

Depolymerization Data 25

Figure S20. ^{1}H NMR spectrum of BHET crystals obtained from depolymerization of PET (DMSO-d$_6$, 400 MHz, 298K). 25

Figure S21. Overlaid ^{1}H NMR spectra (400 MHz, DMSO-d$_6$) of PET depolymerization catalyzed by **LZnK** in ethylene glycol at 180 °C. Spectra correspond to aliquots taken at 5 mins (bottom) and 40 mins (top). Conversion was determined from the integration ratio of the BHET signal at δ = 8.10 ppm (s, 4H) relative to the internal standard NMP signal at δ = 2.71 ppm (s, 3H), using the formula given in Equation (1). 25

Figure S22. Overlaid ^{1}H NMR spectra of PET depolymerization catalyzed by **LZnNa** in ethylene glycol at 180 °C over a 75 minute. 26

Table S7. Depolymerization of PET with ethylene glycol at 180 °C using complexes **LZnNa**, **LZnK**, **LMgNa** and **LMgK**. 26

Figure S23. (a) Conversion *vs* time for PET depolymerization catalyzed by **LZnK** at 120, 150 and 180 °C over a period of 180 minutes. (b) Conversion *vs* time for PET depolymerization catalyzed by **LMgK** at 120, 150 and 180 °C over a period of 180 hours. The inset graph for PET depolymerization catalyzed by **LMgK** at 180 °C shows

the x-axis in minutes, to enable comparison with the **LZnK** catalyzed reaction shown in (a).....27

Figure S24 Conversion *vs* time for PET depolymerization catalyzed by **LZnK** or **LMgK** at 150 °C over a period of 48 hours. 28

Figure S25. Conversion *vs* time for PET depolymerization catalyzed with **LMgK** using PET pellets.....29

Figure S26. Conversion *vs* time of PET catalyzed by **LMgK** heating the system with catalyst, NMP, and ethylene glycol at 180 °C for 5 min and 5 h before PET addition and kinetics data point collection. 30

Figure S27. Conversion *vs* time of PET catalyzed by **LZnK** heating the system with catalyst, NMP, and ethylene glycol at 180 °C for 5 min and 5 h before PET addition and kinetics data point collection. 31

Figure S28. Depolymerization of successive additions of PET using **LZnK**. After complete depolymerization, the system was left to cool down to room temperature for different periods of time prior to restarting the reaction *via* heating to 180 °C for 5 min and adding a new batch of PET (Run 2: immediate addition; Run 3: 24 h; Run 4: 7 days and Run 5: 3 weeks). 32

Figure S29. Comparison of the kinetics curves for the depolymerization of PET with ethylene glycol at 180 °C using catalysts **LMgNa**, **$L_2Mg_3 \cdot 6H_2O$** (denoted as **LMg** in the key) and **LNa_3**. 33

Figure S30. Conversion *vs* time for commercial PET bottle (5 mm^2) and PET powder, catalyzed by (a) **LZnK** and (b) **LMgK** 33

Figure S31. Conversion *vs* time (with a logarithmic x-axis) for commercial PET bottle (5 mm^2) and PET powder depolymerization, catalyzed by a) **LZnK** and b) **LMgK**. 34

Figure S32. a) ^1H NMR spectra of complex **LZnNa** before and after heating at 180 °C for 3 h, (DMSO-d_6, 400 MHz, 298 K). b) TGA isothermal analysis of complex **LZnNa** at 180 °C for 3 h. 34

Figure S33. a) ^1H NMR spectra of complex **LMgNa** before and after heating at 180 °C for 3 h, (DMSO-d_6, 400 MHz, 298 K). b) TGA isothermal analysis of complex **LMgNa** at 180 °C for 3 h. 35

Figure S34. a) ^1H NMR spectra of complex **LZnK** before and after heating at 180 °C for 3 h (DMSO-d_6, 400 MHz, 298 K). b) TGA isothermal analysis of complex **LZnK** at 180 °C for 3 h. 35

Figure S35. a) ^1H NMR spectra of complex **LMgK** before and after heating at 180 °C for 3 h (DMSO-d_6, 400 MHz, 298 K). b) TGA isothermal analysis of complex **LMgK** at 180 °C for 3 h. 36

Figure S36. Overlaid ^1H NMR spectra (400 MHz, DMSO-d□) of PLA depolymerization catalyzed by **LZnK** in ethylene glycol at 180 °C. Spectra correspond to aliquots taken at 2 min (bottom) and 15 min (top). Conversion was determined from the integration ratio of the 2-HEtLa signal at δ = 1.25 ppm (d, 6H) relative to the internal standard NMP signal at δ = 2.71 ppm (s, 3H), using the formula given in Equation (2). 36

Figure S37. Overlaid ^1H NMR spectra of PLA depolymerization catalyzed by complex **LZnNa** in ethylene glycol at 180 °C over a 16 minute period. 37

Polymerization Data 38

Table S8 Ring-opening polymerisation of *rac*-LA with complexes **LZnNa**, **LZnK**, **LMgNa** and **LMgK** in the presence and absence of BnOH.[a] 38

Table S9. Ring-opening polymerisation of *rac*-LA with Zn-based complexes **LZnNa** and **LZnK** at 60 °C in toluene or THF using various time point to determine the reaction kinetics.[a]..39

Table S10. Ring-opening polymerisation of *rac*-LA with Mg-based complexes **LMgNa** and **LMgK** at 60 °C in toluene or THF using various time point to determine the reaction kinetics.[a]..40

Table S11. Ring-opening polymerisation of *rac*-LA with **LZnNa**, **LZnK**, **LMgNa**, **LMgK**, **LH_2Na** and **LH_2K** at 60 °C in toluene or THF to benchmark heterometallic complexes against the homometallic alkali metal analogues.[a] 41

Figure S38. DOSY NMR spectrum (500 MHz) for the stoichiometric combination between complex **LZnNa** and BnOH (1:1) in THF-d_8 at 298 K. 41

Figure S39. MALDI-ToF mass spectrum of PLA produced by **LZnNa** in the presence of BnOH in toluene at 60 °C showing the series of peaks corresponding to (i) linear BnO-PLA-H (blue $[M+K]^+$, yellow $[M+Na]^+$); and (ii) cyclic PLA (grey $[M+K]^+$, orange $[M+Na]^+$). 42

Figure S40. Overlaid ^{1}H NMR spectra (THF-d_8, 500 MHz) for the stoichiometric combination of complex **LMgNa** and BnOH at 298 K. 42

Figure S41. Overlaid ^{1}H NMR spectra (THF-d_8, 500 MHz) for the stoichiometric reaction of complex **LZnNa** and *rac*-LA in the presence or absence of BnOH at 298 K. 43

Figure S42. Proposed mechanism for the ring-opening polymerization of lactide by catalysts **LZnNa**, **LZnK**, **LMgNa** and **LMgK**, in the presence of 1 equiv. of benzyl alcohol (BnOH) as an exogeneous initiator. 44

References..45

Materials

All manipulations involving air- or moisture-sensitive compounds were performed under an argon atmosphere using standard Schlenk-line techniques and gloveboxes. Depolymerization reactions were performed in air in a 20 mL scintillation vial. All reagents purchased from Sigma-Aldrich, Fisher Scientific, Acros Organic or Fluorochem were used as received unless stated otherwise. PET ($M_{n,NMR}$ = 30 100 g mol^{-1}) and PLA ($M_{n,SEC}$ = 61 500 g mol^{-1}, $Đ$ = 2.29) were purchased from Goodfellow Cambridge Limited and used in the form white powder (PET) and white pellets sized 3 – 5 mm (PET and PLA). Dry THF and toluene were collected from a solvent purification system (Innovative Technologies) and stored in the presence of activated molecular sieves (4 Å) under argon. Deuterated NMR solvents (d_8-toluene, d_8-THF and $CDCl_3$) were degassed by three freeze-pump-thaw cycles and stored over activated 4 Å molecular sieves under argon or N_2. *Rac*-lactide (*rac*-LA) was purified by double recrystallisation from toluene followed by sublimation under vacuum. For ^{1}H-NMR of depolymerization aliquots, DMSO-d_6 was purchased from Acros Organics and used as received. TrenSal (**LH_3**) ligand, **LH_2Na**, **LNa_3** and **$L_2Mg_3·6H_2O$** were synthesized according to literature procedures.[1,2]

Characterization and Instrumentation

NMR spectroscopy: For catalyst analysis all 1D (^{1}H, ^{13}C), 2D (COSY, HSQC) and DOSY NMR spectra were recorded on Bruker AVA400, AVA500 and AVA600 spectrometer. For

depolymerization studies, ^{1}H spectra were obtained on a Bruker DPX-400 NMR instrument operating at 400 MHz for ^{1}H and 100.57 MHz for ^{13}C. All spectra were recorded at 298 K, unless stated otherwise. ^{1}H NMR spectra are referred to residual proton solvent (δ_H = 2.50 for DMSO-d_6; 7.26 for $CDCl_3$; 5.02, 3.88 for THF-d_8, 7.09, 7.01, 6.97 and 2.08 for toluene-d_8). The resonance multiplicities in the ^{1}H NMR are described as s (singlet), d (douplet), dd (doublet of doublets), ddd (doublet of doublets of doublets), t (triplet), td (triplet of doublets), tdd (triplet of doublet of doublets), q (quartet) and m (multiplet).

X-ray Crystallography: Single crystal data were collected on a Rigaku Oxford Diffraction Supernova or Bruker D8 Venture diffractometer. Structures were solved using ShelXT and refined with ShelXL-2018.[3,4] Crystal structures figures were generated using Mercury and rendered with POV-Ray software.

Size Exclusion Chromatography (SEC): Molecular weights of polymers were determined by size exclusion chromatography (SEC) in a 1260 Agilent Infinity II GPC/SEC single detection system with mixed bed C PLgel columns (300×7.5 mm) and were calibrated using polystyrene standards with a correction factor of 0.58 for poly(lactic acid).[5]

Matrix-Assisted Laser Desorption/Ionization Time of Flight (MALDI-ToF): MALDI-ToF mass analysis was performed using a Bruker Daltonics UltrafleXtreme™ MALDI-ToF/ToF MS instrument. Dithranol was used as matrix and potassium iodide was added as a cationizing additive.

Thermogravimetric Analysis (TGA): TGA measurements were performed on a Q550 Thermogravimetric analyser (TA Instruments), heating at 10 °C min^{-1} and up to 180 °C and kept at this temperature for 3.5h under N_2 atmosphere.

Experimental Details

Synthesis and characterization of TrenSal complexes **LZnNa, LZnK, LMgNa**, **LMgK** and **LH_2K**

*Synthesis and characterization of heterometallic TrenSal complex **LZnNa***

TrenSal ligand [LH_3] (458.6 mg, 1 mmol) and NaH (26.4 mg, 1.1 mmol) were added to a Schlenk flask, dissolved in dry THF (15 mL) and stirred for 1 hour. The resulting solution was then added dropwise to a $ZnEt_2$ solution (0.10 mL, in 5 mL dry THF, 1 mmol). The resulting mixture was stirred for 3 hours at ambient temperature under an argon atmosphere. The solvent was subsequently removed *in vacuo* resulting in a pale-yellow powder (530.6 mg, 97.6%). Single crystals suitable for X-ray diffraction analysis were obtained *via* cooling a THF solution to -34 °C. ^{1}H NMR (500 MHz, THF-d_8) δ 7.98 (s, 3H, N=CH), 6.89 (dd, J = 7.7, 1.9 Hz, 3H, ArH), 6.85 (ddd, J = 8.7, 6.9, 2.0 Hz, 3H, ArH), 6.35 (dd, J = 8.6, 1.2 Hz, 3H, ArH), 6.14 (ddd, J = 7.9, 6.8, 1.2 Hz, 3H, ArH), 3.80 – 3.70 (m, 3H, CH_2), 3.16 (dd, J = 14.1, 3.3 Hz, 3H, CH_2), 2.76 (dd, J = 11.0, 3.4 Hz, 3H, CH_2), 2.64 (td, J = 13.4, 3.6 Hz, 3H, CH2). ^{13}C NMR (126 MHz, THF-d8) δ 173.43 (C=N), 169.50(C-O), 136.18, 132.53, 124.01, 120.79, 110.64 (Ar-C), 63.17, 60.15 (CH_2). APPI-MS: m/z 543.13 [LZnNa + H]$^+$ (calc: 543.14). Elemental Analysis Calculated for LZnNa (543.91 g mol^{-1}): C 59.62, H 5.00, N 10.30. Found: C 58.79, H 4.88, N 9.77.

*Synthesis and characterization of heterometallic TrenSal complex **LZnK***

TrenSal ligand [LH_3] (458.6 mg, 1 mmol) and KHMDS (199.5 mg, 1 mmol) were added to a Schlenk flask, dissolved in dry THF (15 mL) and stirred for 1 hour. The resulting solution was then added dropwise to a $ZnEt_2$ solution (0.10 mL, in 5 mL dry THF, 1 mmol). The resulting mixture was stirred for 3 hours at ambient temperature under an argon atmosphere. The solvent was subsequently removed *in vacuo* resulting in a white powder (457.8 mg, 83.5%). Single crystals suitable for X-ray diffraction analysis were obtained *via* cooling a THF solution to -34 °C. ^{1}H NMR (500 MHz, THF-d_8) δ 7.98 (s, 3H, N=CH), 6.89 (d, J = 7.0 Hz, 3H, ArH), 6.84 (d, J = 7.8 Hz, 3H, ArH), 6.30 (s, 3H, ArH), 6.13 (t, J = 7.3 Hz, 3H, ArH), 3.80 (s, 3H, CH_2), 3.17 (d, J = 13.8 Hz, 3H, CH_2), 2.81 – 2.71 (m, 3H, CH_2), 2.65 (t, J = 13.6 Hz, 3H, CH_2). ^{13}C NMR (126 MHz, THF-d_8) δ 173.24 (C=N), 169.06 (C-O), 136.32, 132.61, 123.46, 121.05, 110.45 (Ar-C), 63.47, 59.79 (CH_2). APPI-MS: m/z 559.1 [LZnK + H]$^+$ (calc: 559.1). Elemental Analysis Calculated for LZnK·THF (632.15 g mol^{-1}): C 58.90, H 5.58, N 8.86. Found: C 58.92, H 5.46, N 8.36.

The related complexes **$[LZnK]_\infty$** and **$[K(THF)_2(18\text{-}c\text{-}6)]^+[K\{LZn\}_2]^-$** were obtained by recrystallizing **LZnK** in the presence of 2 equivalents of 18-crown-6 ether, from toluene and THF respectively.

*Synthesis and characterization of heterometallic TrenSal complex **LMgNa***

TrenSal ligand [LH_3] (458.6 mg, 1 mmol) and NaHMDS (183.4 mg, 1 mmol) were added to a Schlenk flask, dissolved in dry THF (15 mL) and stirred for 1 hour. The resulting solution was then added dropwise to a $Mg(HMDS)_2$ solution (345.1 in 5 mL dry THF, 1 mmol). The resulting mixture was stirred for 3 hours at ambient temperature under an argon atmosphere. The solvent was subsequently removed *in vacuo* resulting in a white powder (477.2 mg, 94.9%). Single crystals suitable for X-ray diffraction analysis were obtained *via* cooling a THF solution to -34 °C. ^{1}H NMR (500 MHz, THF-d_8) δ 7.97 (s, 3H, N=CH), 6.93 (dd, J = 7.6, 2.0 Hz, 3H, ArH), 6.87 (ddd, J = 8.7, 6.9, 2.0 Hz, 3H, ArH), 6.36 (dd, J = 8.5, 1.2 Hz, 3H, ArH), 6.21 – 6.12 (m, 3H, ArH), 3.73 (tdd, J = 10.0, 4.0, 2.7 Hz, 3H, CH_2), 3.13 (dd, J = 13.7, 3.2 Hz, 3H, CH_2), 2.75 (dd, J = 10.9, 3.3 Hz, 3H, CH2), 2.59 (td, J = 13.4, 3.5 Hz, 3H, CH2). ^{13}C NMR (126 MHz,

THF-d8) δ 171.90 (C=N), 169.29 (C-O), 135.81, 132.64, 123.62, 122.08, 110.83 (Ar-C), 63.24, 60.60 (CH_2). APPI-MS: m/z 503.18 [LMgNa + H]$^+$ (calc: 503.20). Elemental Analysis Calculated for LMgNa (502.83 g mol^{-1}): C 64.49, H 5.41, N 11.14. Found: C 63.40, H 5.14, N 10.20.

*Synthesis and characterization of heterometallic TrenSal complex **LMgK***

TrenSal ligand [LH_3] (458.6 mg, 1 mmol) and KHMDS (199.5 mg, 1 mmol) were added to a Schlenk flask, dissolved in dry THF (15 mL) and stirred for 1 hour. The resulting solution was then added dropwise to a $Mg(HMDS)_2$ solution (345.1 in 5 mL dry THF, 1 mmol). The resulting mixture was stirred for 3 hours at ambient temperature under an argon atmosphere. The solvent was subsequently removed *in vacuo* resulting in a white powder (549.1 mg, 92.9%). Single crystals suitable for X-ray diffraction analysis were obtained *via* cooling a THF solution to -34 °C. ^{1}H NMR (500 MHz, THF-d8) δ 7.96 (s, 3H, N=CH), 6.93 (dd, J = 7.6, 2.0 Hz, 3H, ArH), 6.87 (ddd, J = 8.7, 6.8, 2.0 Hz, 3H, ArH), 6.27 (d, J = 8.4 Hz, 3H, ArH), 6.15 (t, 3H, ArH), 3.86 – 3.75 (m, 3H, CH_2), 3.11 (dd, J = 13.7, 3.1 Hz, 3H, CH2), 2.75 (dd, J = 11.0, 3.2 Hz, 3H, CH_2), 2.59 (td, J = 13.4, 3.4 Hz, 3H, CH_2). ^{13}C NMR (126 MHz, THF-d_8) δ 171.74 (C=N), 168.71 (C-O), 136.00, 132.78, 122.81, 122.38, 110.55 (Ar-C), 63.49, 60.17 (CH_2). APPI-MS: m/z 663.27 [LMgK + H + 2 x THF]$^+$ (calc: 663.45). Elemental Analysis Calculated for LMgK·THF (591.05 g mol^{-1}): C 63.00, H 5.97, N 9.48. Found: C 62.60, H 5.78, N 8.98.

*Synthesis and characterization of homometallic TrenSal complex **LH_2K***

TrenSal ligand [LH_3] (459 mg, 1 mmol) and KHMDS (200 mg, 1 mmol) were weighed into a Schlenk flaskand dissolved in dry toluene (20 mL) in the glove box. The resulting mixture was stirred for 16 hours at room temperature under an argon atmosphere. The solvent was subsequently removed *in vacuo*, resulting in a yellow powder. Complex **LH_2K**: (433 mg, 87%). ^{1}H NMR (500 MHz, d_6-DMSO) δ 14.13 (s, 2H, OH), 8.48 (s, 3H, N=CN), 7.24 (dd, J = 7.4, 1.8 Hz, 3H, ArH), 7.09 (td, J = 8.3, 1.8 Hz, 3H, ArH), 6.62 (d, J = 8.3 Hz, 3H, ArH), 6.46 (t, J = 7.4 Hz, 3H, ArH), 3.55 (t, J = 6.4 Hz, 6H, CH_2), 2.80 (t, J = 6.4 Hz, 6H, CH_2). ^{13}C{^{1}H} NMR (126 MHz, d_6-DMSO) δ 164.2 (C=N), 137.3 (C-O), 131.6, 129.4, 120.6, 118.5, 114.5 (ArC), 58.3, 55.4 (CH_2). Elemental analysis calculation for [LH_2K + 2/7 HMDSH] (542.76 g mol^{-1}): C 63.54, H 6.39, O 11.06; found: C 63.81, H 6.10, N 10.54. APPI-MS: m/z [M + H]$^+$ 497.35; calculated [M + H]$^+$: 497.19.

Depolymerization of PET powder

A 20 mL scintillation glass vial containing a magnetic stirrer was charged with 0.01 equiv. of catalyst, 2.8 mL (50 mmol, 20 equiv.) of ethylene glycol and 0.024 mL (0.25 mmol, 0.10 equiv.) of NMP were added. The vial with the mixture was sealed, placed in a metallic heating block and heated to the desired temperature (180, 150 or 120 °C) while stirring at 500 rpm for 5 min to allow the heterogeneous reaction mixture temperature to stabilize. After this time, 480 mg (2.5 mmol, 1 equiv.) of PET was added and the vial was sealed again and the time was set. Aliquots of 0.1 mL were collected and measured by ^{1}H NMR in 0.6 mL of DMSO-d_6. The depolymerization conversion was followed by comparing the signal δ_H = 8.10 (s, 4H) of bis(2 hydroxyethylene)terephthalate (BHET) and δ_H = 2.71 (s, 3H) from NMP as already reported in the literature.[6] At the end of the reaction, the system was cooled at room temperature and water was added to precipitate BHET formed. BHET, ^{1}H-NMR (400 MHz, DMSO-d_6, 298 K) δ = 8.12 (s, 4H; Ar-H), 4.96 (t, 2H; OH), 4.32 (m, 4H; CH_2), 3.72 (m, 4H; CH_2).

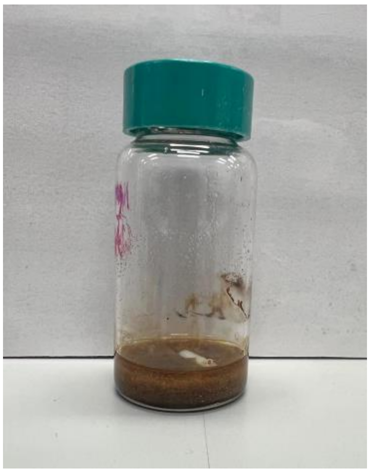

Figure S1. Experimental set-up for PET depolymerization, highlighting the heterogeneous nature.

Polymerization of rac-LA in THF or toluene solvent

In the glovebox, the relevant homo- or heterometallic complex (0.02 mmol), *rac*-LA (0.288 g, 2 mmol), BnOH (2.07 µL, 0.02 mmol) and a magnetic stirrer bar were placed in an air-tight vial. The polymerisation was initiated by adding the appropriate solvent (THF or toluene, 2.0 mL) to the mixture. The reaction was subsequently stirred under the appropriate reaction conditions and quenched by adding the reaction to excess hexane after a set time period. The volatiles were removed using compressed air and an aliquot of the crude product was dissolved in $CDCl_3$ for ^1H NMR spectroscopic analysis. Monomer conversion was calculated based on the ratio of monomer to polymer. The reaction was repeated for each time point until duplicate results were observed. SEC, $^1H\{^1H\}$ NMR and MALDI-TOF analysis were then performed for one set of the generated polymer for different conversions.

Bulk polymerization of L-LA

In the glovebox, a series of identical reaction mixtures were prepared in air-tight glass vials, each containing 0.01 equiv. of the relevant catalyst (0.02 mmol), LA (0.288 g, 2 mmol), BnOH (2.07 µL, 0.02 mmol) and a magnetic stirrer bar. The vials were sealed and removed from the glovebox, then placed on a preheated aluminium heating block maintained at 180 °C. At predetermined time intervals, individual vials were removed from the heat source to quench the reaction by rapid cooling to room temperature, followed by the addition of excess hexane. The volatiles were removed using compressed air and the crude products were dissolved in $CDCl_3$ for ^1H NMR spectroscopic analysis.

Depolymerization of PLA

A 20 mL glass vial containing a magnetic stirrer was charged with 0.01 equiv. of catalyst, 2.8 mL (50 mmol, 20 equiv.) of ethylene glycol and 0.024 mL (0.25 mmol, 0.10 equiv.) of NMP were added. The vial with the mixture was sealed, placed in an aluminium heating block and heated to 180 °C while stirring at 500 rpm for 5 min to allow the reaction mixture temperature to stabilize. After this time, 180 mg (2.5 mmol, 1 equiv.) of PLA was added and the vial was sealed again and the time was set. Aliquots of 0.1 mL were collected and measured by ^1H NMR in 0.6 mL of DMSO-d_6. The depolymerization conversion was followed by comparing the signal δH = 1.25 (d, 6H) of lactide and δH = 2.71 (s, 3H) from NMP (refer to Figure S36 for details).

Supplementary Figures and Tables

Literature reports of mono-, bi- and multimetallic catalysts for polyester depolymerization

Schiff base ligand

a)

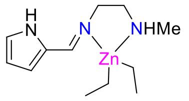

b)

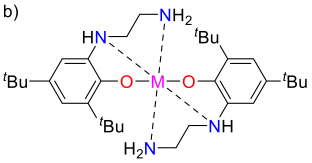

M = Zn, Mg

c)

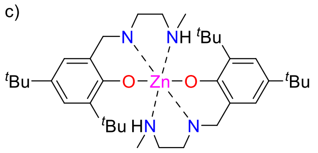

a) *Angew. Chem. Int. Ed.* **2025**, *64*, e202502845

b) *Macromolecules*, **2021**, *54*, 8453 – 8469

c) *ACS Catal.* **2019**, *9*, 409 – 416

Pincer ligand

a)

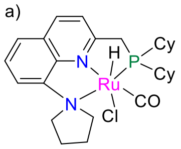

b)

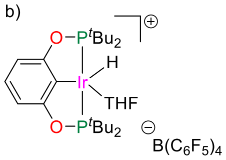

c)

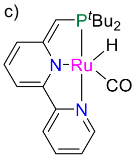

a) Angew. Chem. Int. Ed. **2023**, *62*, e202312564

b) ACS Sustain. Chem. Eng. **2018**, *6*, 10481 – 10488

c) *Chem. Commun.* **2014**, *50*, 4884 – 4887

Guanidine ligand

a)

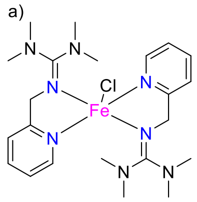

b)

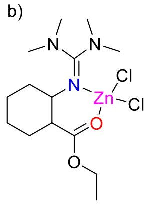

c)

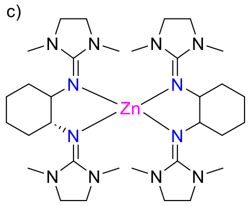

a) Catal. Sci. Technol. **2023**, *13*, 6006 – 6021

b) ChemSusChem **2023**, *16*, e202300192

c) *Angew. Chem. Int. Ed.* **2020**, *59*, 21778 – 21784

Phosphine ligand

a)

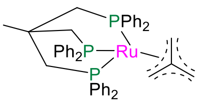

b)

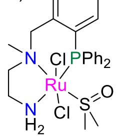

a) Sci. Adv. **2018**, *4*, eaat9669

b) Chem. Eur. J. **2015**, *21*, 10851 – 10860

NHC ligand

a)

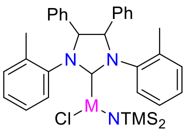

M = Zn, Mg.HNTMS$_2$

b)

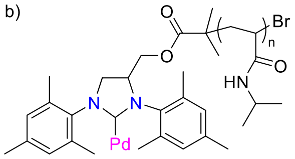

a) *Catal. Sci. Technol*. **2025**, *15*, 822 – 835

b) *J. Appl. Polym. Sci.* **2022**, *139*, e52474

Figure S2. Key ligand classes of monometallic catalysts featuring ancillary ligands for PLA and PET depolymerization.[7–19]

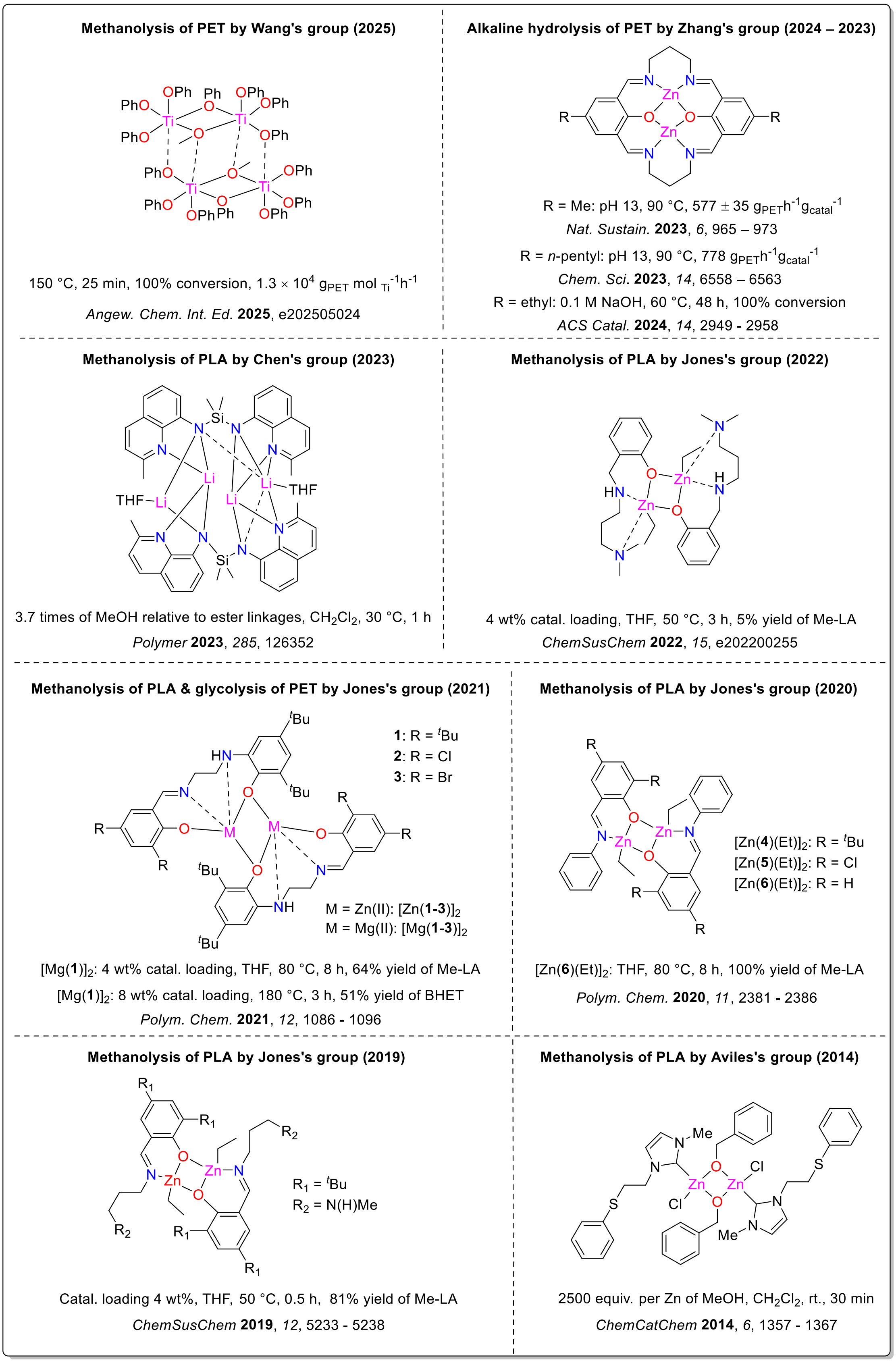

Figure S3. Comprehensive literature examples of homobimetallic and homomultimetallic catalysts for PLA and PET depolymerization.[20–30]

Crystallographic Data

Table S1. Crystallographic data and refinement detail for complexes **LZnNa**, **LMgNa** and **LMgK**.

Complexes	**LZnNa**	**LMgNa**	**LMgK**
Formula	$C_{41}H_{55}N_4NaO_{6.5}Zn$	$C_{41}H_{55}MgN_4NaO_{6.5}$	$C_{62}H_{70}K_2Mg_2N_8O_8$
**μ**/mm^{-1}	0.682	0.910	2.181
Formula Weight	796.25	755.19	1182.08
Size/mm^3	0.154 × 0.094 × 0.084	0.094 × 0.064 × 0.04	0.08 × 0.03 × 0.02
T/K	120.00(10)	120.00(10)	120.00(10)
Crystal System	triclinic	triclinic	tetragonal
Space Group	*P-1*	*P*-1	$I4_1/a$
a/Å	12.8905(18)	12.9938(11)	30.7539(6)
b/Å	13.2627(18)	13.2964(9)	30.7539(6)
c/Å	13.3187(18)	13.3495(11)	12.2015(4)
α/°	119.252(4)	119.367(8)	90
β/°	91.254(4)	91.144(7)	90
γ/°	91.062(4)	91.348(6)	90
V/Å^3	1985.0(5)	2008.2(3)	11540.1(6)
Z	2	2	8
Measured Refl's.	79572	33326	16587
Indep't Refl's	7298	16178	3086
Parameters	524	525	370
Restraints	32	85	0
GooF	1.038	0.937	1.026
wR_2 (all data)	0.2031	0.2144	0.1617
wR_2	0.2125	0.1964	0.1447
R_1 (all data)	0.0804	0.1342	0.0782
R_1	0.0714	0.0820	0.0577

$R_1 = \Sigma|F_o| - |F_c|/\Sigma|F_o|$, $wR_2 = [\Sigma(F_o^2 - F_c^2)^2/\Sigma w(F_o^2)^2]^{1/2}$.

Table S2. Crystallographic data and refinement detail for complexes **LZnK**, **[LZnK]$_\infty$** and **[K(THF)$_2$(18-c-6)]$^+$[K{LZn}$_2$]$^-$**.

Complexes	**LZnK**	**[LZnK]$_\infty$**	**[K(THF)$_2$(18-c-6)]$^+$[K{LZn}$_2$]$^-$**
Formula	$C_{62}H_{70}K_2N_8O_8Zn_2$	$C_{27}H_{27}KN_4O_3Zn$	$C_{86}H_{118}K_2N_8O_{17}Zn_2$
**μ**/mm^{-1}	2.832	1.191	0.728
Formula Weight	1264.20	559.99	1744.82
Size/mm^3	0.305 × 0.059 × 0.043	0.16×0.12×0.08	0.39×0.29×0.18
T/K	120.01(10)	120.00(10)	120.00(10)
Crystal System	tetragonal	monoclinic	triclinic
Space Group	$I4_1/a$	$P2_1/n$	*P*-1
a/Å	30.7481(2)	13.9857(4)	12.7953(2)
b/Å	30.7481(2)	10.0598(3)	17.6698(3)
c/Å	12.19520(10)	17.7453(5)	20.7633(3)
α/°	90	90	91.2940(10)
β/°	90	94.438(3)	105.5790(10)
γ/°	90	90	107.530(2)
V/Å^3	11529.90(18)	2489.16(12)	4284.46(13)
Z	8	4	2
Measured Refl's.	60005	26280	163097
Indep't Refl's	6012	5631	28610
Parameters	482	433	1200
Restraints	2	0	657
GooF	1.032	1.061	1.047
wR_2 (all data)	0.1150	0.0776	0.1669
wR_2	0.1117	0.0713	0.1467
R_1 (all data)	0.0435	0.0662	0.0833
R_1	0.0408	0.0442	0.0594

$R_1 = \Sigma|F_o| - |F_c|/\Sigma|F_o|$, $wR_2 = [\Sigma(F_o^2 - F_c^2)^2/\Sigma w(F_o^2)^2]^{1/2}$.

Table S3. Selected bond lengths (Å) for complexes **LZnNa**, **LMgNa** and **LMgK**.

Bond	LZnNa	Bond	LMgNa	Bond	LMgK
Zn1–O1	2.098(3)	Mg35–O32	2.026(3)	K36–Mg35	3.7411(17)
Zn1–O2	2.105(3)	Mg35–O33	2.025(3)	K36–O32	2.690(3)
Zn1–O3	2.094(3)	Mg35–O34	2.024(3)	K36–O33[1]	2.751(3)
Zn1–N1	2.154(3)	Mg35–N6	2.202(4)	K36–O33	2.793(3)
Zn1–N2	2.152(3)	Mg35–N8	2.211(4)	K36–O34[1]	2.723(3)
Zn1–N3	2.141(4)	Mg35–N10	2.205(4)	K36–O37	2.655(4)
Na1–O1	2.334(3)	Na36–O32	2.381(3)	Mg35–O32	2.010(3)
Na1–O2	2.347(3)	Na36–O33	2.360(3)	Mg35–O33	2.063(3)
Na1–O3	2.341(3)	Na36–O34	2.369(3)	Mg35–O34	2.034(3)
Na1–O4	2.378(4)	Na36–O37	2.398(4)	Mg35–N6	2.241(4)
Na1–O5	2.387(4)	Na36–O42	2.369(4)	Mg35–N8	2.232(4)
Na1–O6	2.363(4)	Na36–O47	2.368(4)	Mg35–N10	2.255(4)

[1]-X,1-Y,1-Z

Table S4. Selected bond lengths (Å) for complexes **LZnK, [LZnK]$_\infty$** and **[K(THF)$_2$(18-c-6)]$^+$[K{LZn}$_2$]$^-$**.

Bond	LZnK	Bond	[LZnK]$_\infty$	Bond	[K(THF)$_2$(18-c)]$^+$ [K{LZn}$_2$]$^-$
Zn35–K36[1]	3.8262(4)	Zn35–O32	2.0932(16)	Zn35–O32	2.0869(15)
Zn35–O32	2.0560(13)	Zn35–O33	2.1098(17)	Zn35–O33	2.0770(14)
Zn35–O33	2.1344(13)	Zn35–O34	2.0961(17)	Zn35–O34	2.0818(15)
Zn35–O34	2.0858(12)	Zn35–N6	2.140(2)	Zn35–N6	2.1776(17)
Zn35–N6	2.1940(15)	Zn35–N8	2.166(2)	Zn35–N8	2.1842(18)
Zn35–N8	2.1819(14)	Zn35–N10	2.240(2)	Zn35–N10	2.1630(18)
Zn35–N10	2.1930(15)	K36–O32	2.8931(17)	Zn37–O82	2.0815(15)
K36–K36[1]	3.5465(7)	K36–O32[1]	2.6974(17)	Zn37–O83	2.0880(15)
K36–O32	2.6828(12)	K36–O33	2.6318(18)	Zn37–O84	2.0796(15)
K36–O33[1]	2.7459(14)	K36–O34	2.5627(17)	Zn37–N56	2.1813(19)
K36–O33	2.7552(13)	K36–N8[1]	3.450(2)	Zn37–N58	2.1834(17)
K36–O34[1]	2.6996(12)	K36–C7[1]	3.397(3)	Zn37–N60	2.1787(19)
		K36–C21[1]	3.200(2)	K36–O32	2.7301(15)
		K36–C23[2]	3.424(3)	K36–O33	2.7760(16)
		K36–C24[2]	3.316(3)	K36–O34	2.7008(16)
				K36–O82	2.7118(16)
				K36–O83	2.7217(16)
				K36–O84	2.8008(16)

[1]-X,1-Y,1-Z

Table S5. Selected bond angles (°) for complexes **LZnNa**, **LMgNa**, **LZnK**, **LMgK, [LZnK]$_\infty$** and **[K(THF)$_2$(18-c-6)]$^+$[K{LZn}$_2$]$^-$**.

LZnNa					
**Atoms**	**Angle/°**	**Atom**	**Angle/°**	**Atoms**	**Angle/°**
O1–Zn1–O2	85.42(12)	O3–Zn1–N3	86.40(12)	O2–Na1–O6	95.11(13)
O1–Zn1–N1	86.29(12)	N2–Zn1–N1	99.07(13)	O3–Na1–O2	74.73(11)
O1–Zn1–N2	88.08(13)	N3–Zn1–N1	99.69(14)	O3–Na1–O4	97.53(13)
O1–Zn1–N3	169.35(12)	N3–Zn1–N2	99.56(13)	O3–Na1–O5	93.38(12)
O2–Zn1–N1	169.72(13)	O1–Na1–O2	75.05(11)	O3–Na1–O6	163.38(13)
O2–Zn1–N2	86.73(12)	O1–Na1–O3	74.57(11)	O4–Na1–O5	95.03(13)
O2–Zn1–N3	87.62(13)	O1–Na1–O4	166.83(14)	O6–Na1–O4	96.05(15)
O3–Zn1–O1	85.01(11)	O1–Na1–O5	95.93(12)	O6–Na1–O5	94.92(14)
O3–Zn1–O2	85.30(11)	O1–Na1–O6	90.25(13)	Zn1–O1–Na1	84.22(11)
O3–Zn1–N1	87.93(12)	O2–Na1–O4	92.84(12)	Zn1–O2–Na1	83.76(11)
O3–Zn1–N2	169.84(12)	O2–Na1–O5	166.52(13)	Zn1–O3–Na1	84.14(10)
**LMgNa**					
**Atom**	**Angle/°**	**Atom**	**Angle/°**	**Atom**	**Angle/°**
O32–Mg35–N6	88.92(14)	O34–Mg35–N10	172.49(15)	O34–Na36–O37	93.94(13)
O32–Mg35–N8	172.21(15)	N6–Mg35–N8	97.97(15)	O42–Na36–O32	93.54(13)
O32–Mg35–N10	84.91(14)	N6–Mg35–N10	97.91(14)	O42–Na36–O34	96.46(14)
O33–Mg35–O32	87.95(13)	N10–Mg35–N8	97.68(14)	O42–Na36–O37	96.32(14)
O33–Mg35–N6	172.14(14)	O32–Na36–O37	164.31(14)	O47–Na36–O32	95.07(14)
O33–Mg35–N8	84.76(13)	O33–Na36–O32	72.78(11)	O47–Na36–O34	161.73(14)
O33–Mg35–N10	88.99(13)	O33–Na36–O34	72.83(11)	O47–Na36–O37	95.63(14)

O34–Mg35–O32	88.20(13)	O33–Na36–O37	95.62(13)	O47–Na36–O42	97.91(15)
O34–Mg35–O33	87.78(13)	O33–Na36–O42	164.47(14)	Mg35–O32–Na36	83.12(11)
O34–Mg35–N6	84.92(13)	O33–Na36–O47	90.75(13)	Mg35–O33–Na36	83.69(11)
O34–Mg35–N8	88.78(14)	O34–Na36–O32	72.79(11)	Mg35–O34–Na36	83.49(11)
**LMgK**					
**Atom**	**Angle/˚**	**Atom**	**Angle/˚**	**Atom**	**Angle/˚**
O34^{1}–K36–O33^{1}	64.00(9)	O32–Mg35–N10	83.87(13)	N6–Mg35–K36	133.64(10)
O34^{1}–K36–O33	104.89(9)	O33–Mg35–K36^{1}	45.69(9)	N6–Mg35–N10	92.03(13)
O37–K36–Mg35	135.65(9)	O33–Mg35–K36	47.39(9)	N8–Mg35–K36^{1}	88.77(10)
O37–K36–O32	108.61(11)	O33–Mg35–N6	174.27(14)	N8–Mg35–K36	131.18(11)
O37–K36–O33^{1}	111.80(11)	O33–Mg35–N8	83.86(13)	N8–Mg35–N6	94.54(14)
O37–K36–O33	148.78(12)	O33–Mg35–N10	93.50(13)	N8–Mg35–N10	91.58(13)
O37–K36–O34^{1}	84.65(10)	O34–Mg35–K36	88.52(9)	N10–Mg35–K36	93.61(10)
K36–Mg35–K36^{1}	57.40(3)	O34–Mg35–K36^{1}	44.71(8)	N10–Mg35–K36^{1}	138.91(10)
O32–Mg35–K36^{1}	91.06(9)	O34–Mg35–O33	90.17(12)	Mg35–O32–K36	104.58(12)
O32–Mg35–K36	44.09(9)	O34–Mg35–N6	84.30(13)	K36^{1}–O33–K36	81.07(8)
O32–Mg35–O33	90.92(13)	O34–Mg35–N8	89.30(13)	Mg35–O33–K36	99.69(12)
O32–Mg35–O34	95.60(13)	O34–Mg35–N10	176.29(14)	Mg35–O33–K36^{1}	101.86(12)
O32–Mg35–N6	91.11(13)	N6–Mg35–K36^{1}	128.91(10)	Mg35–O34–K36^{1}	103.60(12)
O32–Mg35–N8	172.87(14)				
**LZnK**					
**Atom**	**Angle/˚**	**Atom**	**Angle/˚**	**Atom**	**Angle/˚**
O32–Zn35–K36^{1}	87.90(3)	N8–Zn35–K36^{1}	87.64(4)	O34^{1}–K36–Zn35^{1}	31.70(3)
O32–Zn35–O33	87.90(5)	N8–Zn35–N6	96.46(5)	O34^{1}–K36–K36^{1}	82.31(3)
O32–Zn35–O34	91.98(5)	N8–Zn35–N10	93.40(5)	O34^{1}–K36–O33	104.75(4)
O32–Zn35–N6	90.87(5)	N10–Zn35–K36^{1}	137.21(4)	O34^{1}–K36–O33^{1}	64.63(4)
O32–Zn35–N8	172.67(5)	N10–Zn35–N6	93.87(5)	O37–K36–Zn35^{1}	100.83(4)
O32–Zn35–N10	85.93(5)	K36^{1}–K36–Zn35^{1}	61.829(10)	O37–K36–K36^{1}	162.09(4)
O33–Zn35–K36^{1}	44.47(4)	O32–K36–Zn35^{1}	140.72(3)	O37–K36–O32	108.39(5)
O33–Zn35–N6	172.94(5)	O32–K36–K36^{1}	85.53(3)	O37–K36–O33^{1}	113.20(5)
O33–Zn35–N8	84.85(5)	O32–K36–O33	64.66(4)	O37–K36–O33	146.33(5)
O33–Zn35–N10	92.97(5)	O32–K36–O33^{1}	109.20(4)	O37–K36–O34^{1}	84.12(4)
O34–Zn35–K36^{1}	42.84(3)	O32–K36–O34^{1}	167.49(4)	Zn35–O32–K36	105.68(5)
O34–Zn35–O33	87.23(5)	O33–K36–Zn35^{1}	103.19(3)	Zn35–O33–K36	101.07(5)
O34–Zn35–N6	85.86(5)	O33^{1}–K36–Zn35^{1}	32.99(3)	Zn35–O33–K36^{1}	102.54(5)
O34–Zn35–N8	88.70(5)	O33^{1}–K36–K36^{1}	49.97(3)	K36^{1}–O33–K36	80.29(4)
O34–Zn35–N10	177.90(5)	O33–K36–K36^{1}	49.74(3)	Zn35–O34–K36^{1}	105.46(5)
N6–Zn35–K36^{1}	128.55(4)	O33^{1}–K36–O33	99.72(4)		
**[LZnK]$_{\infty}$**					
**Atoms**	**Angle/˚**	**Atom**	**Angle/˚**	**Atoms**	**Angle/˚**
O32–Zn35–O33	89.56(6)	032–K36–N8^{1}	127.37(5)	O34–K36–O32	63.57(5)
O32–Zn35–O34	87.15(6)	O32–K36–C7^{1}	121.92(6)	O34–K36–O33	65.99(5)
O32–Zn35–N6	171.99(7)	O32^{1}–K36–C7^{1}	72.13(6)	O34–K36–N8^{1}	111.77(5)
O32–Zn35–N8	87.11(7)	O32–K36–C21^{1}	148.13(6)	O34–K36–C7^{1}	134.40(6)
O32–Zn35–N10	82.83(7)	O32^{1}–K36–C21^{1}	23.67(5)	O34–K36–C21^{1}	89.76(6)

O33–Zn35–N6	93.85(7)	O32^{1}–K36–C23^{2}	134.85(6)	O34–K36–C23^{2}	129.95(6)
O33–Zn35–N8	84.52(7)	O32–K36–C23^{2}	71.51(6)	O34–K36–C24^{2}	149.05(7)
O33–Zn35–N10	167.90(7)	O32^{1}–K36–C24^{2}	111.47(6)	C7^{1}–K36–N8^{1}	24.78(5)
O34–Zn35–O33	84.55(7)	O32–K36–C24^{2}	94.95(6)	C7^{1}–K36–C23^{2}	87.74(7)
O34–Zn35–N6	85.97(7)	O33–K36–C32	64.60(5)	C21^{1}–K36–N8^{1}	77.13(6)
O34–Zn35–N8	167.68(7)	O33–K36–C32^{1}	111.08(5)	C21^{1}–K36–C7^{1}	88.93(7)
O34–Zn35–N10	85.68(7)	O33–K36–N8^{1}	166.49(5)	C21^{1}–K36–C23^{2}	121.90(6)
N6–Zn35–N8	100.42(8)	O33–K36–C7^{1}	159.54(6)	C21^{1}–K36–C24^{2}	101.20(7)
N6–Zn35–N10	92.57(8)	O33–K36–C21^{1}	89.45(6)	C23^{2}–K36–N8^{1}	112.50(6)
N8–Zn35–N8	104.39(8)	O33–K36–C23^{2}	75.87(6)	C24^{2}–K36–N8^{1}	98.93(6)
O32^{1}–K36–O32	153.07(3)	O33–K36–C24^{2}	85.08(7)	C24^{2}–K36–C7^{1}	75.24(7)
O32^{1}–K36–N8^{1}	55.43(5)	O34–K36–O32^{1}	90.03(5)	C24^{2}–K36–C23^{2}	23.45(6)
**$[K(THF)_2(18\text{-}c\text{-}6)]^+[K\{LZn\}_2]^-$**					
**Atom**	**Angle/˚**	**Atom**	**Angle/˚**	**Atom**	**Angle/˚**
O32–Zn35–N6	86.20(6)	O82–Zn37–N58	85.47(6)	O34–Zn36–O33	63.61(4)
O32–Zn35–N8	172.33(6)	O82–Zn37–N60	85.34(6)	O34–Zn36–O82	112.82(5)
O32–Zn35–N10	85.84(6)	O83–Zn37–N56	84.86(7)	O34–Zn36–O83	177.07(5)
O33–Zn35–O32	90.06(6)	O83–Zn37–N58	85.45(6)	O34–Zn36–O84	116.66(5)
O33–Zn35–O34	87.93(6)	O83–Zn37–N60	170.52(6)	O82–Zn36–O32	176.84(5)
O33–Zn35–N6	172.87(6)	O84–Zn37–O82	89.84(6)	O82–Zn36–O33	112.89(4)
O33–Zn35–N8	85.80(6)	O84–Zn37–O83	87.55(6)	O82–Zn36–O83	64.29(5)
O33–Zn35–N10	84.87(6)	O84–Zn37–N56	85.59(6)	O82–Zn36–O84	64.40(4)
O34–Zn35–O32	88.03(6)	O84–Zn37–N58	171.72(6)	O83–Zn36–O32	118.41(5)
O34–Zn35–N6	85.88(6)	O84–Zn37–N60	85.90(7)	O83–Zn36–O33	116.65(5)
O34–Zn35–N8	85.38(7)	N56–Zn37–N58	98.18(7)	O83–Zn36–O84	62.93(4)
O34–Zn35–N10	170.53(6)	N60–Zn37–N56	101.44(7)	Zn35–O32–K36	88.36(5)
N6–Zn35–N8	97.22(7)	N60–Zn37–N58	100.49(7)	Zn35–O33–K36	87.33(5)
N10–Zn35–N6	100.89(7)	O32–Zn36–O33	64.68(4)	Zn35–O34–K36	89.26(5)
N10–Zn35–N8	100.19(7)	O32–Zn36–O84	118.01(5)	Zn37–O82–K36	89.65(5)
O82–Zn37–O83	87.80(6)	O33–Zn36–O84	177.26(5)	Zn37–O83–K36	89.25(5)
O82–Zn37–N56	171.51(7)	O34–Zn36–O32	64.46(5)	Zn37–O84–K36	87.29(5)

^{1}-X,1-Y,1-Z

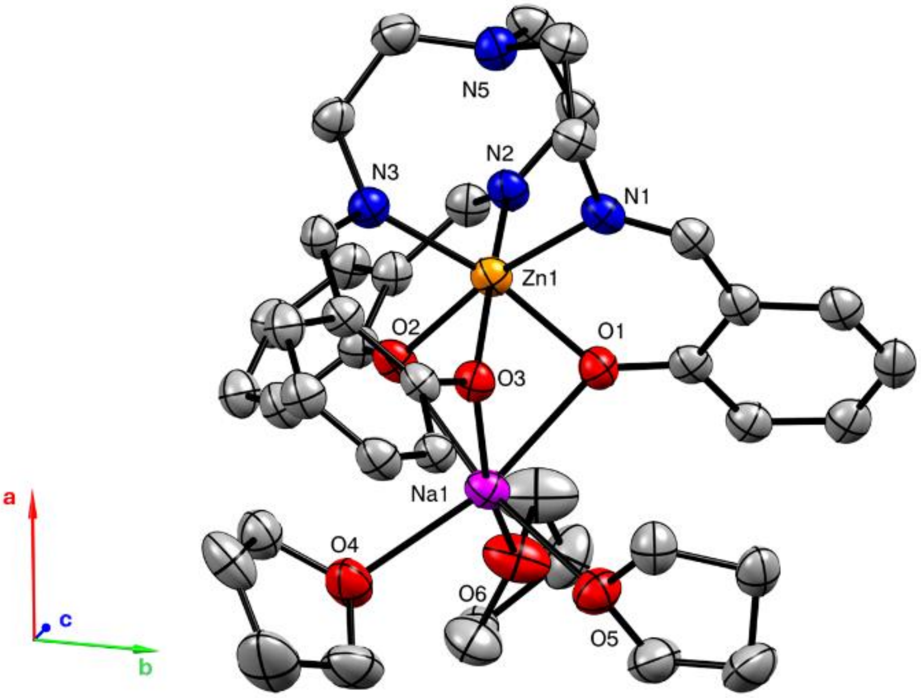

Figure S4. Molecular structure of complex **LZnNa**·3THF crystallized from THF solvent with displacement ellipsoids at the 50% probability level. Hydrogen atoms, solvents and disorder in the THF are omitted for clarity.

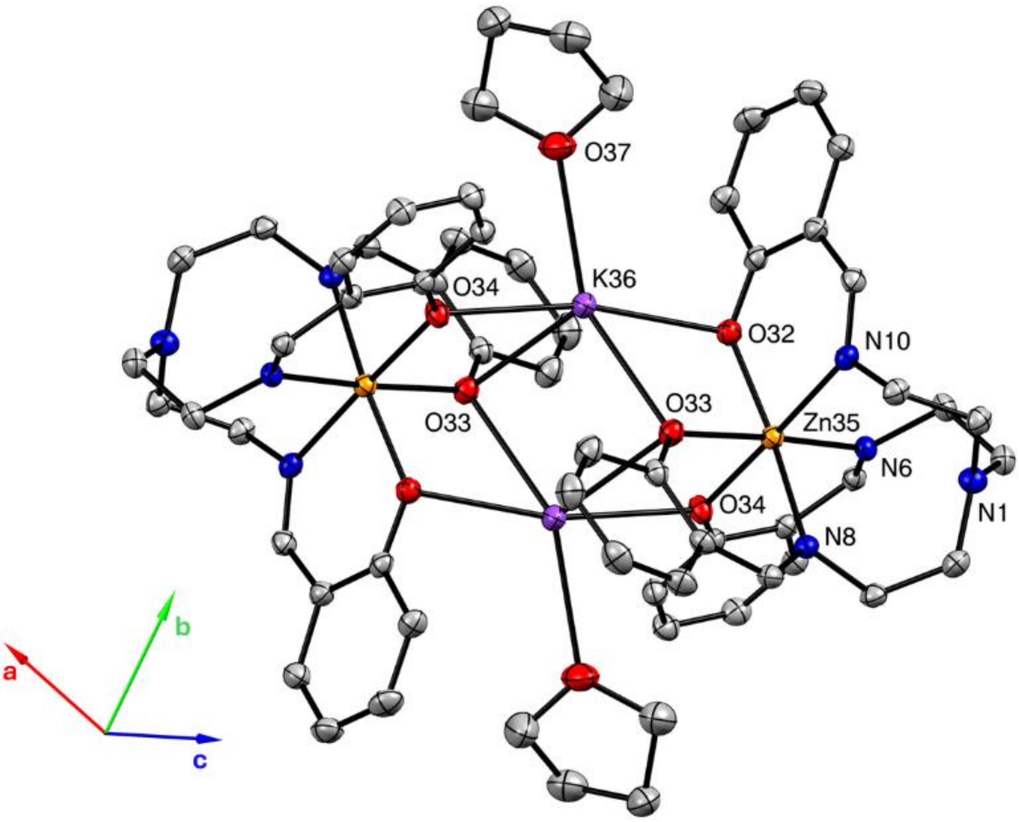

Figure S5. Molecular structure of complex [**LZn$_2$K**]$_2$·2THF crystallized from THF solvent with displacement ellipsoids at the 50% probability level. Hydrogen atoms and solvents are omitted for clarity.

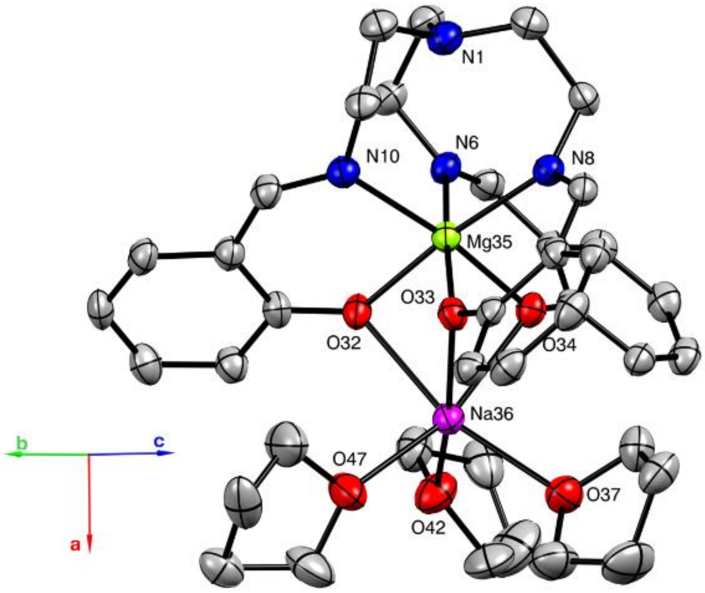

Figure S6. Molecular structure of complex **LMgNa**·3THF crystallized from THF solvent with displacement ellipsoids at the 50% probability level. Hydrogen atoms, solvents and disorder in the THF are omitted for clarity.

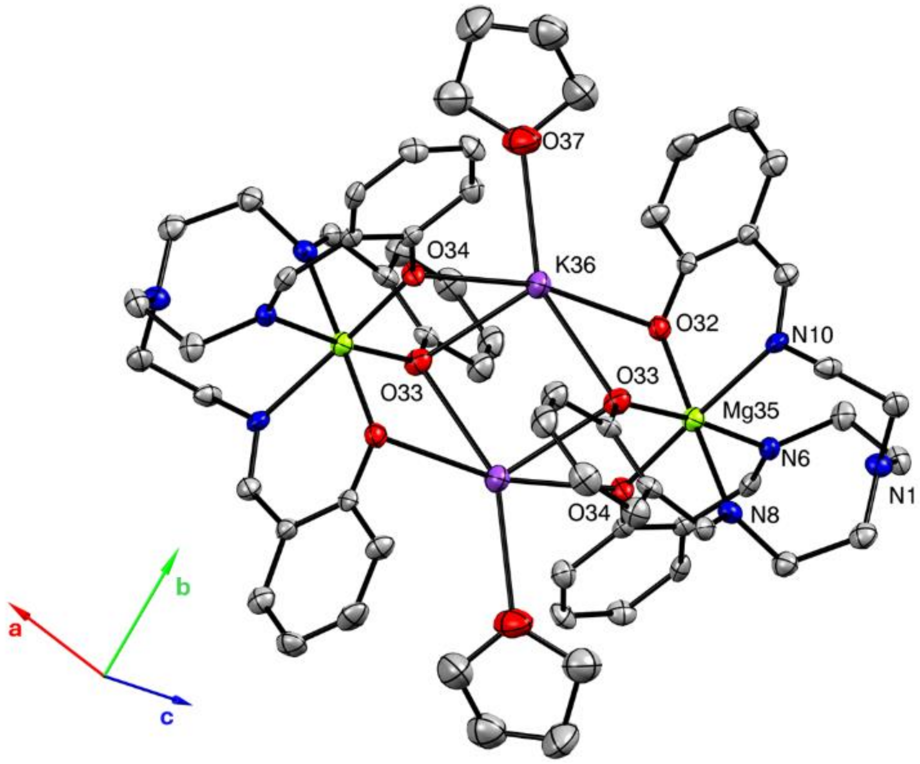

Figure S7. Molecular structure of complex [**LMgK**]$_2$·2THF crystallized from THF solvent with displacement ellipsoids at the 50% probability level. Hydrogen atoms, solvents and disorder in the THF are omitted for clarity.

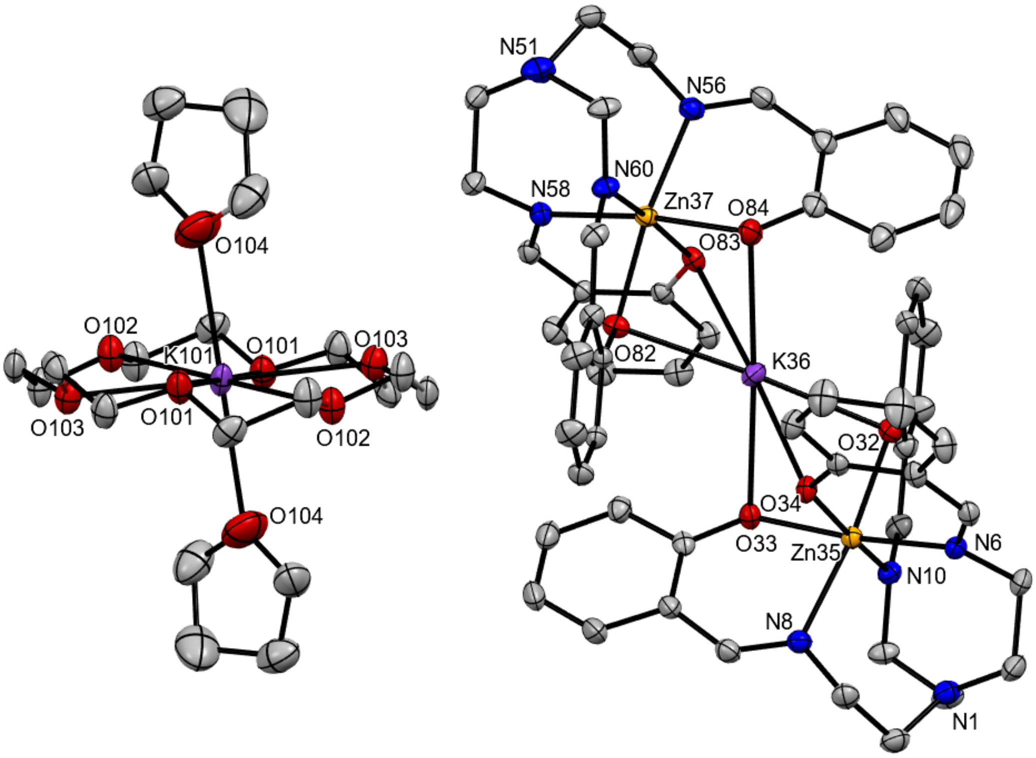

Figure S8. Molecular structure of complex **LZnK** crystallized from THF in the presence of 2 equivalents of 18-crown-6 ether shown with chemically correct proportions, denoted as **[K(THF)$_2$(18-c-6)]$^+$[K{LZn}$_2$]$^-$** with displacement ellipsoids at the 50% probability level. Hydrogen atoms and solvents are omitted for clarity.

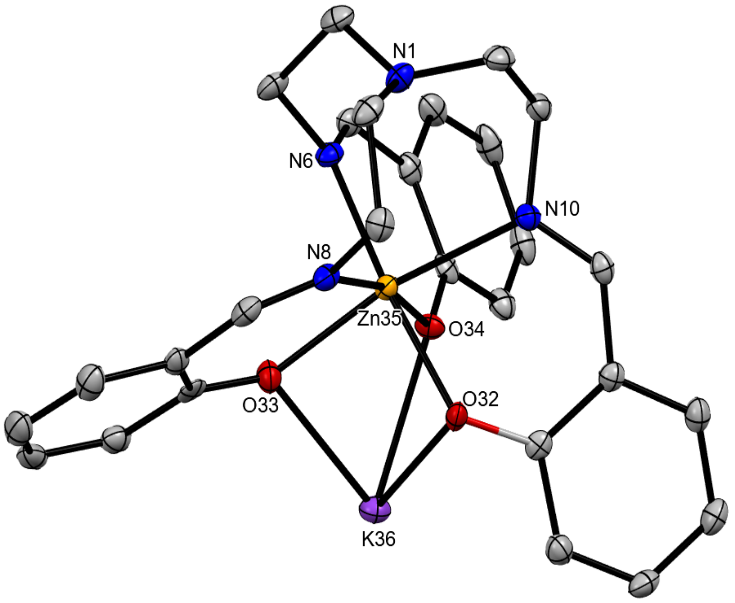

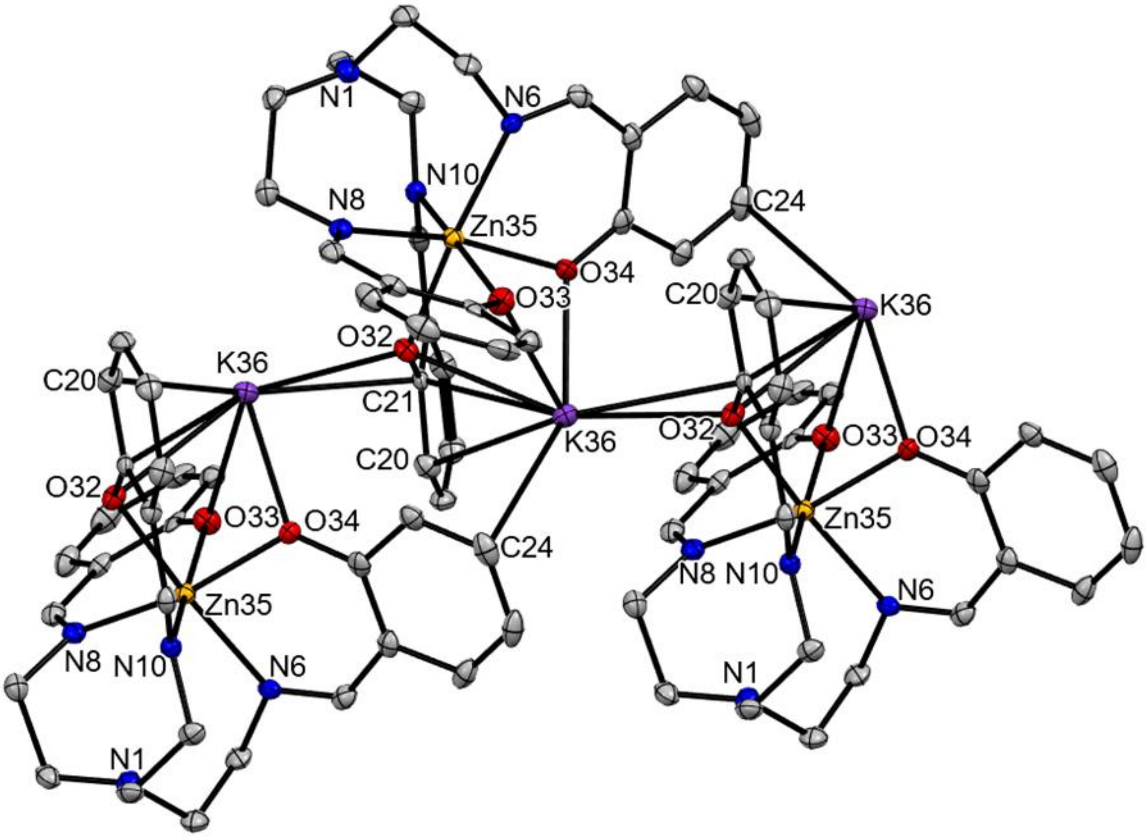

Figure S9. Molecular structure of the unit cell (top) and the polymeric structure (bottom) of complex **LZnK** crystallized from toluene in the presence of 2 equivalents of 18-crown-6 ether, denoted **[LZnK]$_\infty$**, with displacement ellipsoids at the 50% probability level. Hydrogen atoms and solvents are omitted for clarity.

^{1}H and ^{13}C NMR Spectra

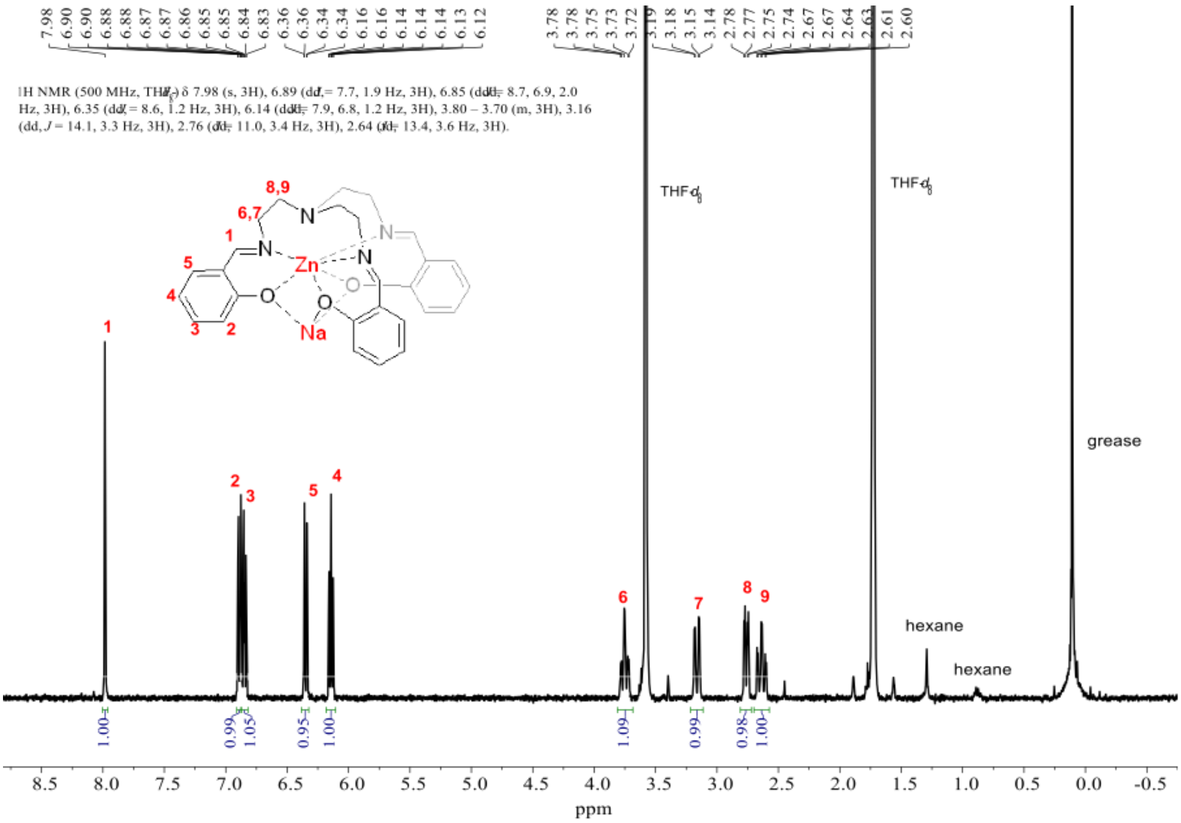

Figure S10. ^{1}H NMR spectrum of **LZnNa** (THF-d_8, 500 MHz, 298 K).

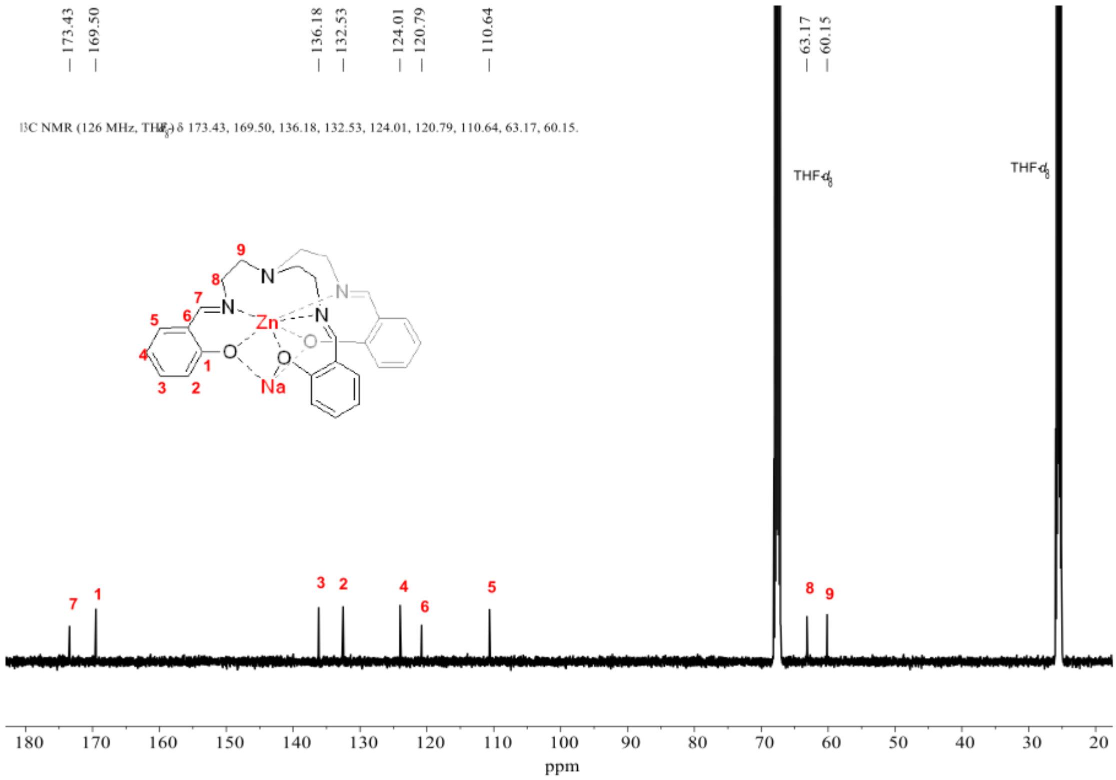

Figure S11. ^{13}C NMR spectrum of **LZnNa** (THF-d_8, 126 MHz, 298 K).

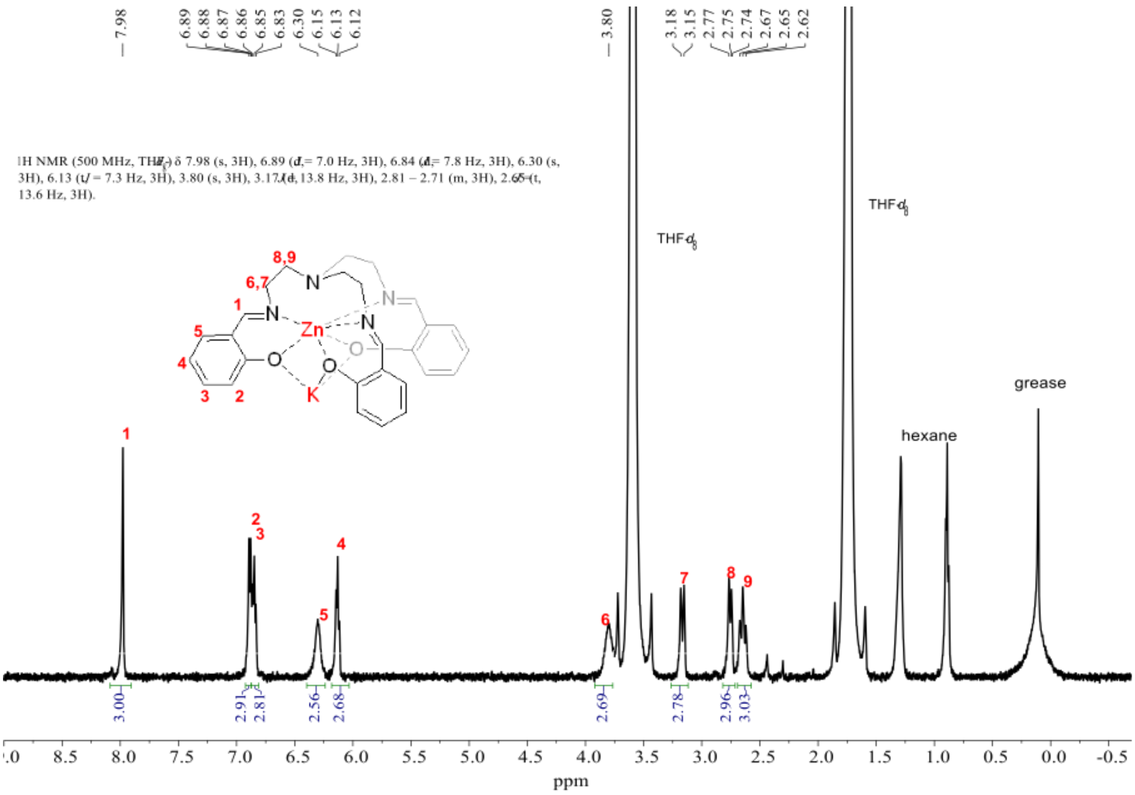

Figure S12. ^{1}H NMR spectrum of **LZnK** (THF-d$_8$, 500 MHz, 298 K).

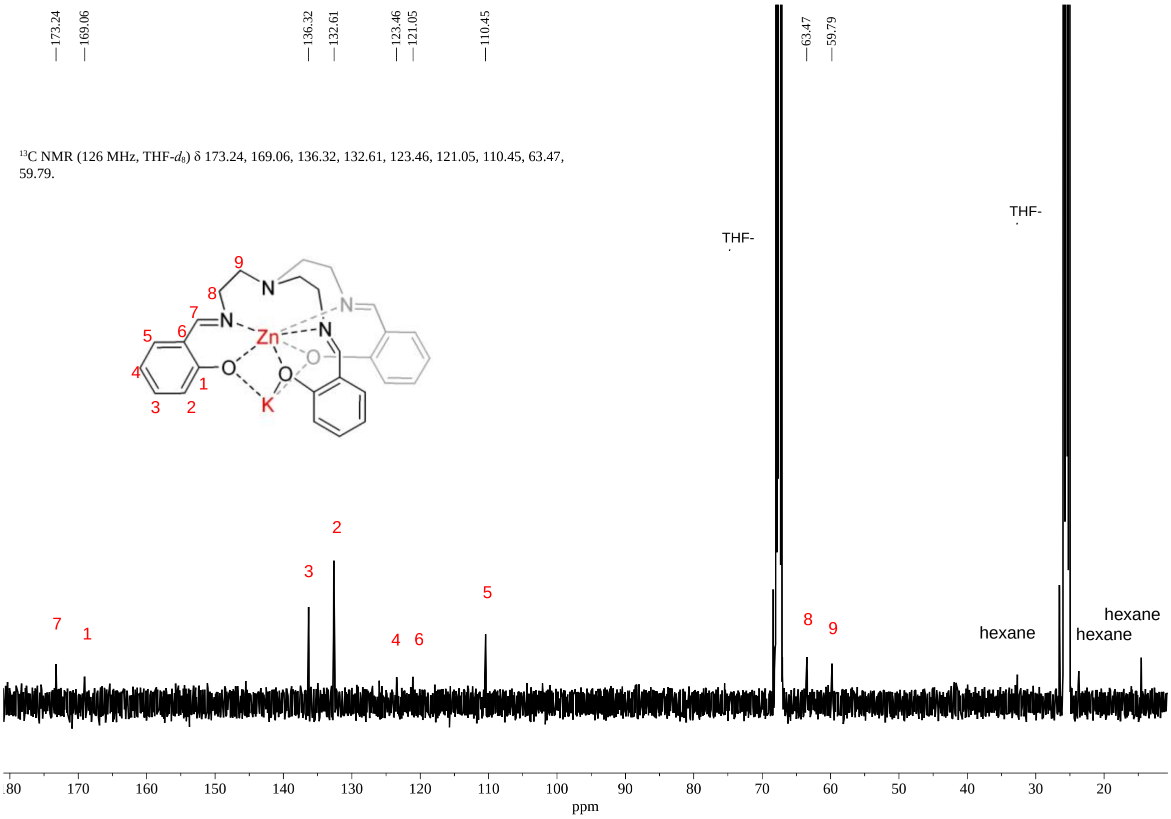

Figure S13. ^{13}C NMR spectrum of **LZnK** (THF-d$_8$, 126 MHz, 298 K).

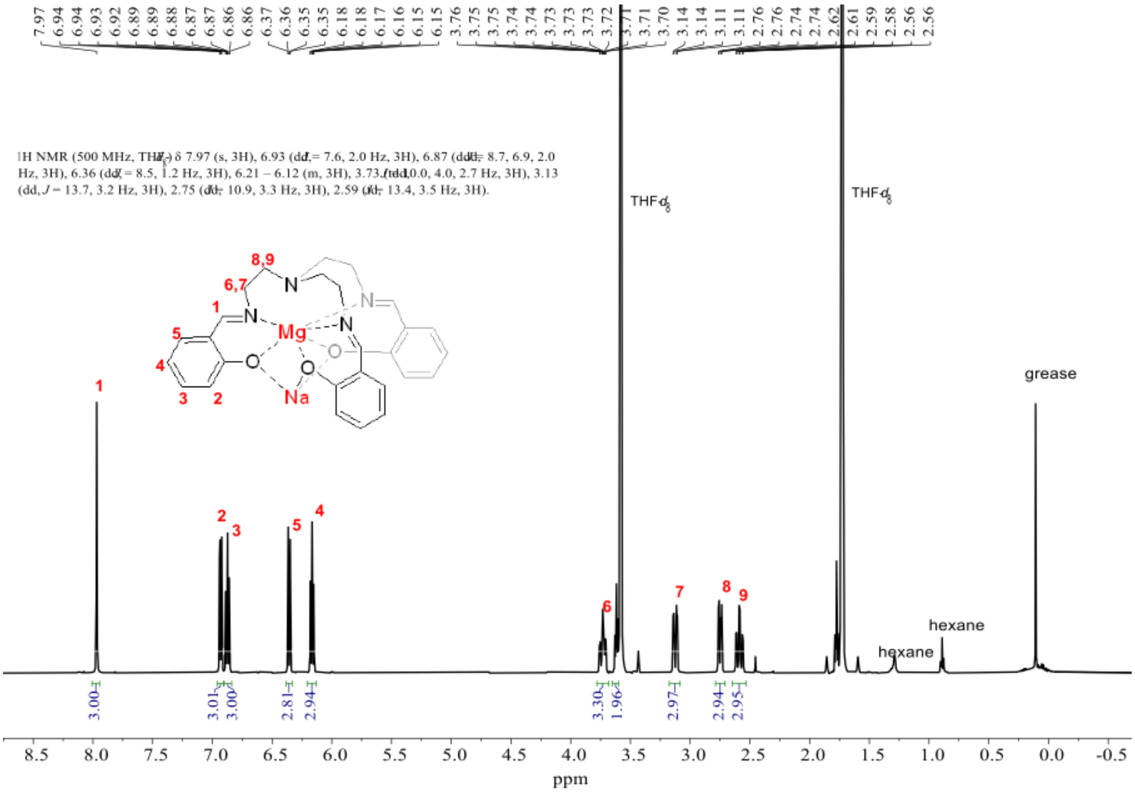

Figure S14. ^{1}H NMR spectrum of **LMgNa** (THF-d$_8$, 500 MHz, 298 K).

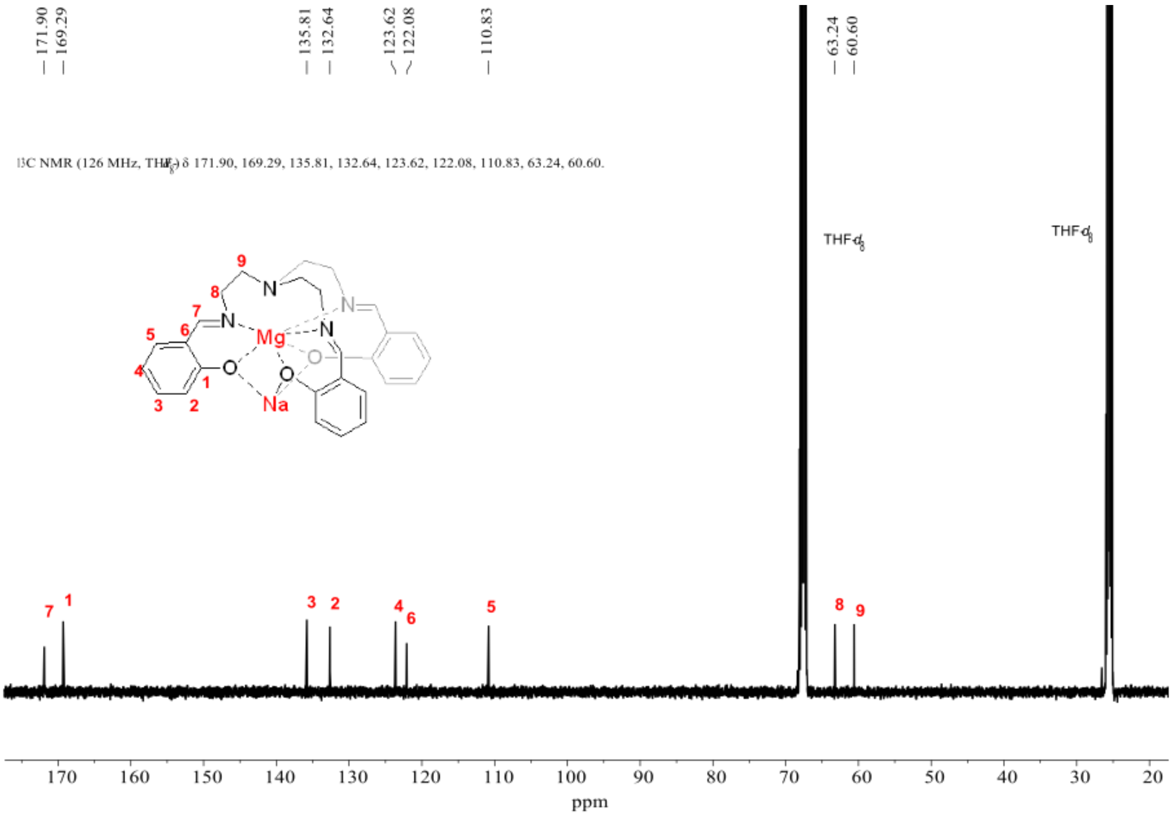

Figure S15. ^{13}C NMR spectrum of **LMgNa** (THF-d$_8$, 126 MHz, 298 K).

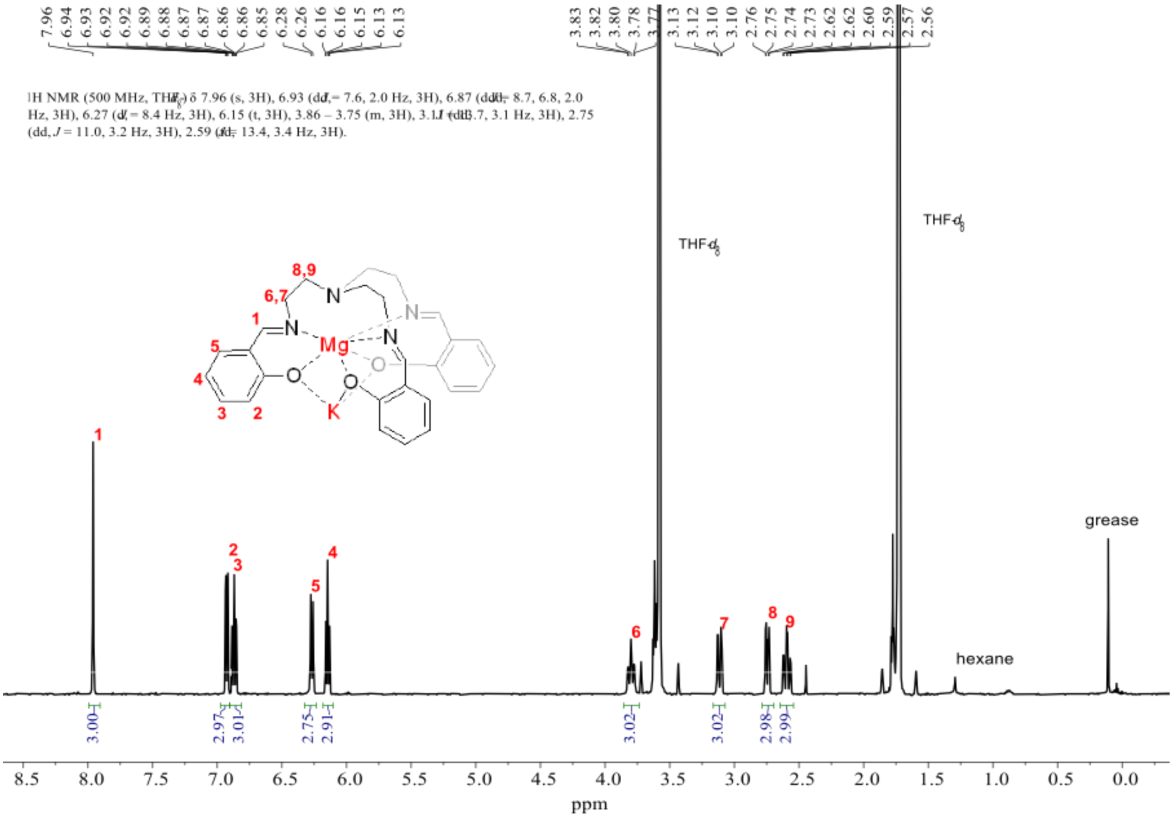

Figure S16. ^{1}H NMR spectrum of **LMgK** (THF-d_8, 500 MHz, 298 K).

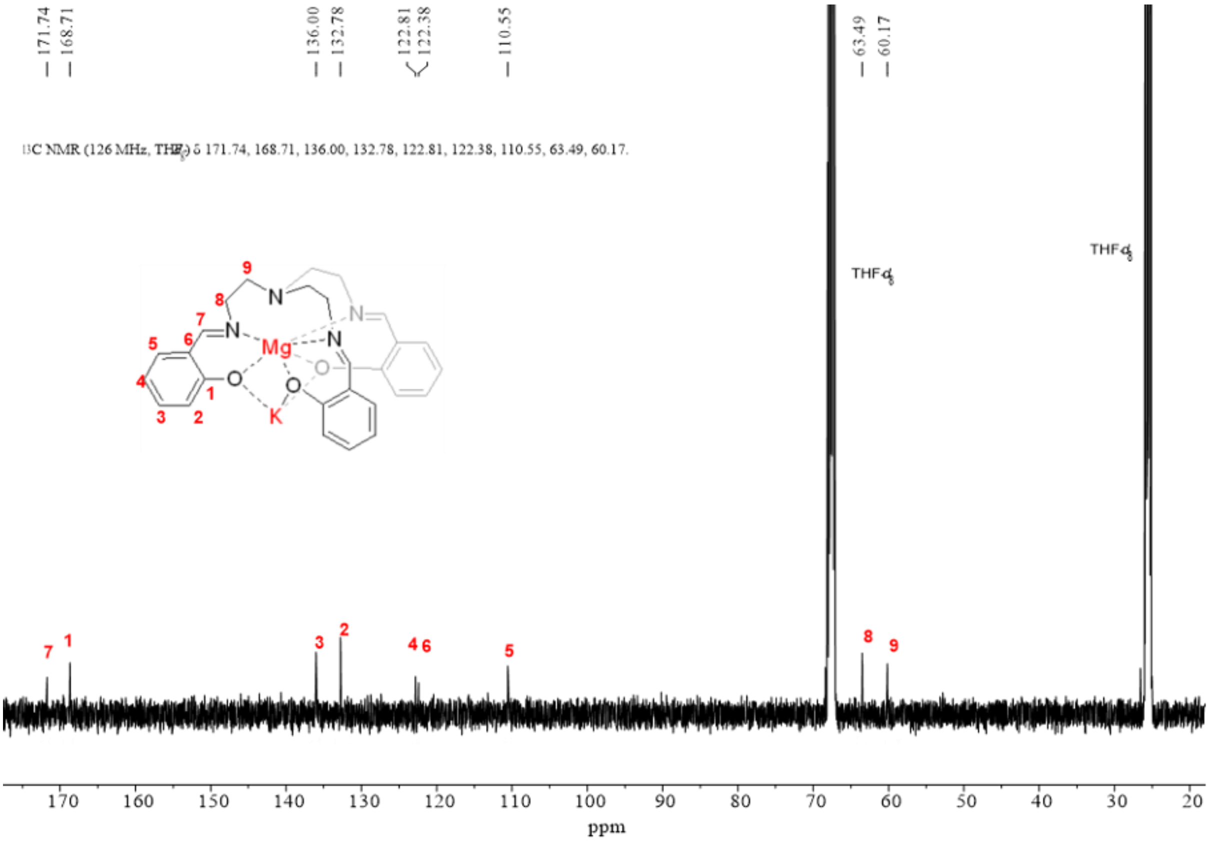

Figure S17. ^{13}C NMR spectrum of **LMgK** (THF-d_8, 126 MHz, 298 K).

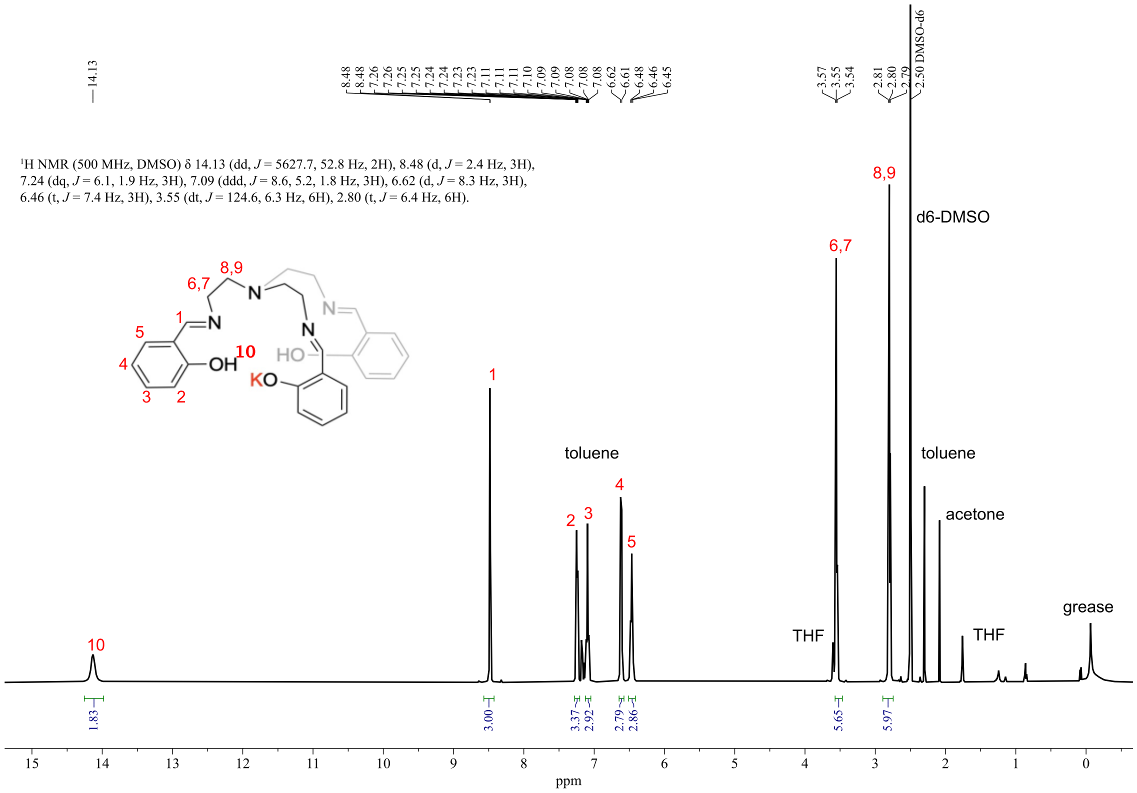

Figure S18. ^{1}H NMR spectrum of **LH_2K** (DMSO-d6, 500 MHz, 298 K).

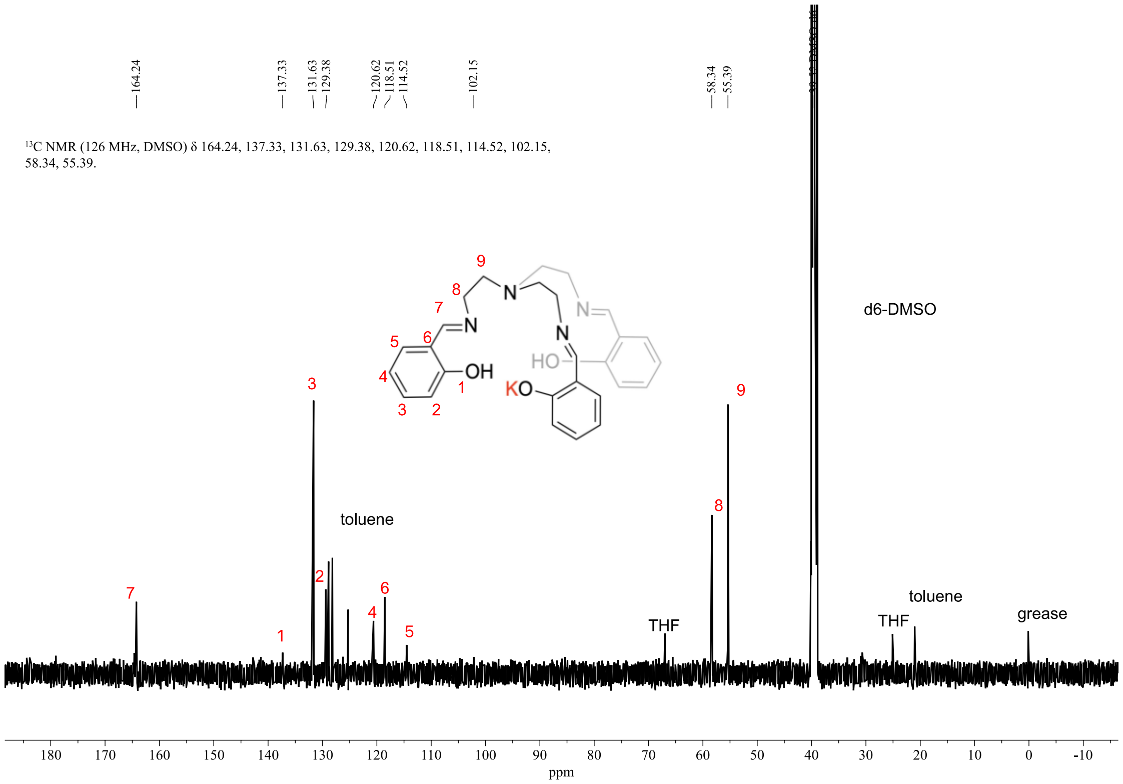

Figure S19. ^{13}C NMR spectrum of **LH_2K** (DMSO-d6, 126 MHz, 298 K).

Table S6. Diffusion coefficients and predicted molecular weights for complexes **LZnNa**, **LZnK**, **LMgNa**, **LMgK**, **LH_2Na** and **LH_2K** (THF-d_8, 500 MHz, 298 K).[a]

Complex	logD / $m^2 s^{-1}$	MW_{obs}/g mol^{-1}	MW_{calc}/ g mol^{-1}
**LZnNa**	-9.020	450	543.91
**LZnK**	-9.039	480	560.02
**LMgNa**	-9.033	470	502.83
**LMgK**	-9.039	480	591.05
**LH_2Na**	-9.014	440	480.54
**LH_2K**	-9.961	520	496.65

[a]MW_{obs} was obtained from the DOSY NMR analysis (THF-d_8, 500 MHz, 298 K) by comparison of the diffusion coefficient observed (logD value) to a calibration plot of known standards.[1]

Depolymerization Data

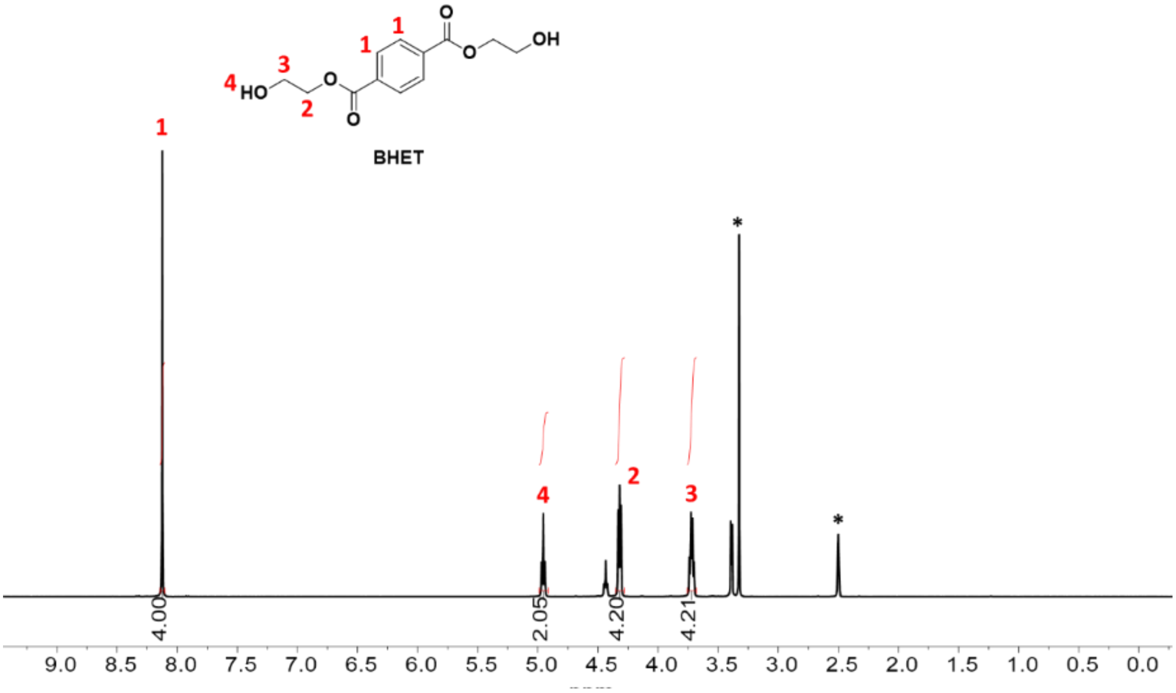

Figure S20. ^{1}H NMR spectrum of BHET crystals obtained from depolymerization of PET (DMSO-d_6, 400 MHz, 298K).

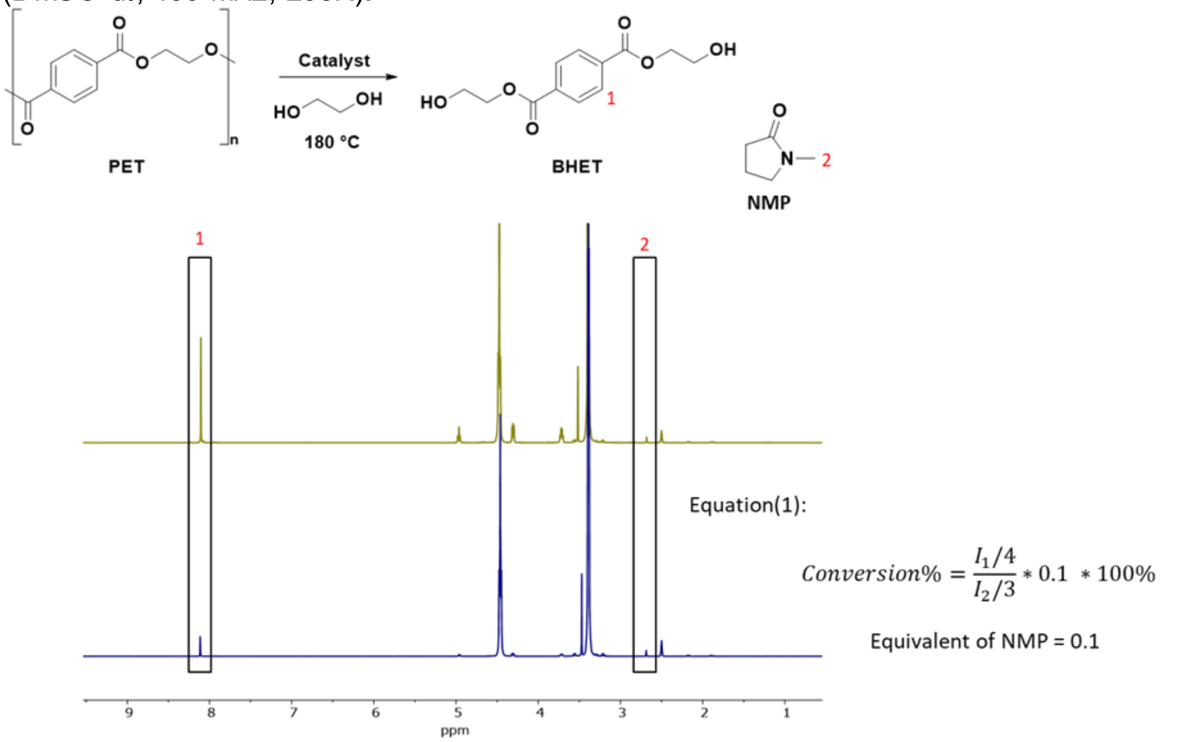

Figure S21. Overlaid ^{1}H NMR spectra (400 MHz, DMSO-d_6) of PET depolymerization catalyzed by **LZnK** in ethylene glycol at 180 °C. Spectra correspond to aliquots taken at 5 mins (bottom) and 40 mins (top). Conversion was determined from the integration ratio of the BHET signal at δ = 8.10 ppm (s, 4H) relative to the internal standard NMP signal at δ = 2.71 ppm (s, 3H), using the formula given in Equation (1).

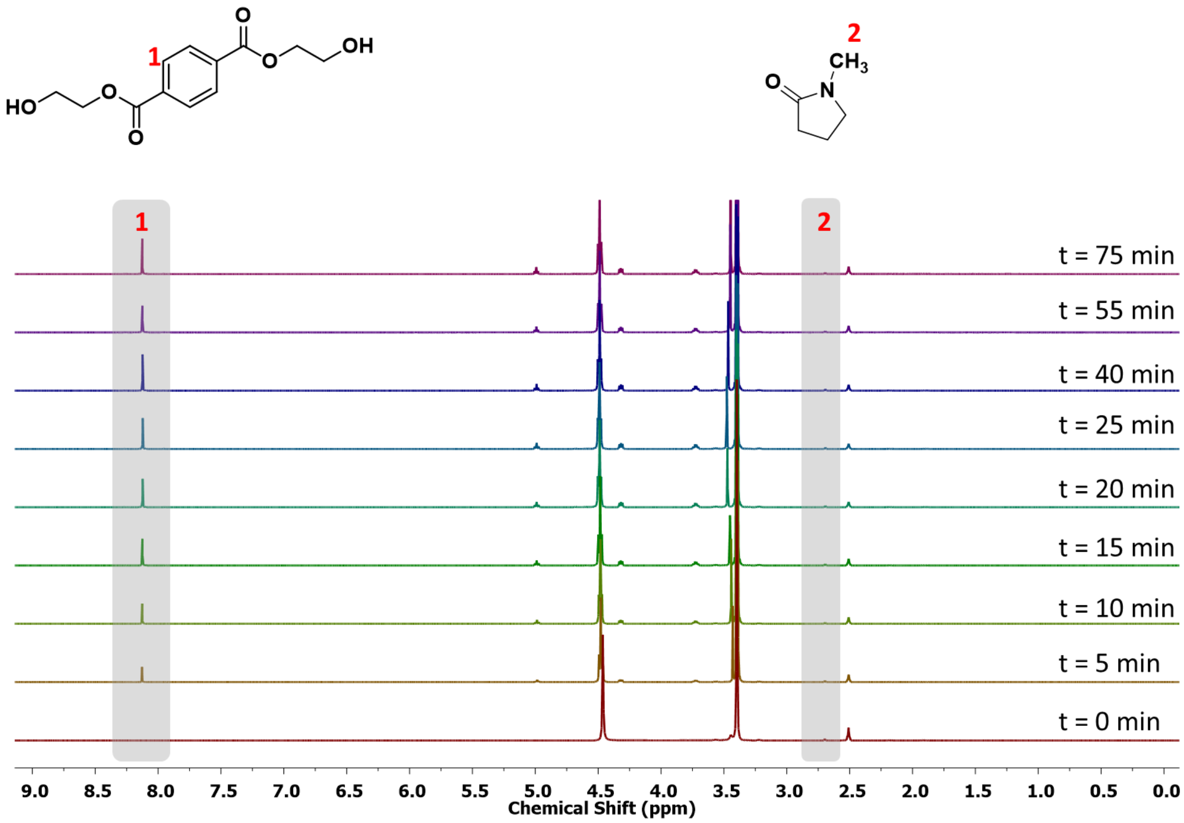

Figure S22. Overlaid ^{1}H NMR spectra of PET depolymerization catalyzed by **LZnNa** in ethylene glycol at 180 °C over a 75 minute.

Table S7. Depolymerization of PET with ethylene glycol at 180 °C using complexes **LZnNa**, **LZnK**, **LMgNa** and **LMgK**.

Entry	Time/ min	%Conv.[a]				
		**LMgK**	**LMgNa**	**LZnK**	**LZnNa**	**LMgK**
1	0	0	0	0	0	0
2	5	45.0	39.2	57.5	51.2	45.0
3	10	64.2	56.3	78.8	74.1	64.2
4	15	79.5	69.7	87.7	86.7	79.5
5	20	88.7	81.7	95.0	92.2	88.7
6	25	93.2	94.2	96.6	95.6	93.2
7	40	>99.9	>99.9	99.4	97.9	>99.9
8	55	>99.9	>99.9	99.6	98.5	>99.9

[a] Determined by ^{1}H NMR spectroscopy.

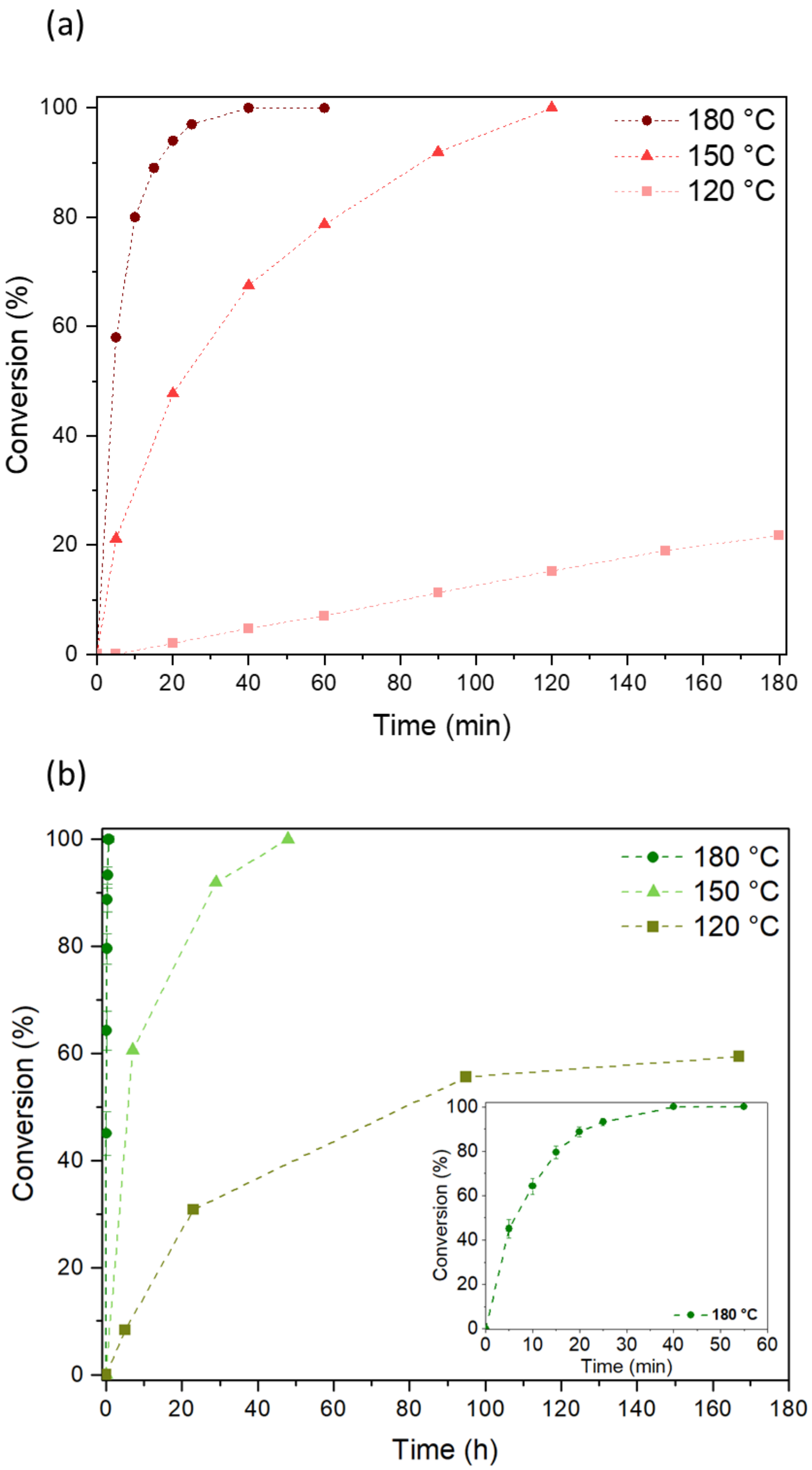

Figure S23. (a) Conversion *vs* time for PET depolymerization catalyzed by **LZnK** at 120, 150 and 180 °C over a period of 180 minutes. (b) Conversion *vs* time for PET depolymerization catalyzed by **LMgK** at 120, 150 and 180 °C over a period of 180 hours. The inset graph for PET depolymerization catalyzed by **LMgK** at 180 °C shows the x-axis in minutes, to enable comparison with the **LZnK** catalyzed reaction shown in (a).

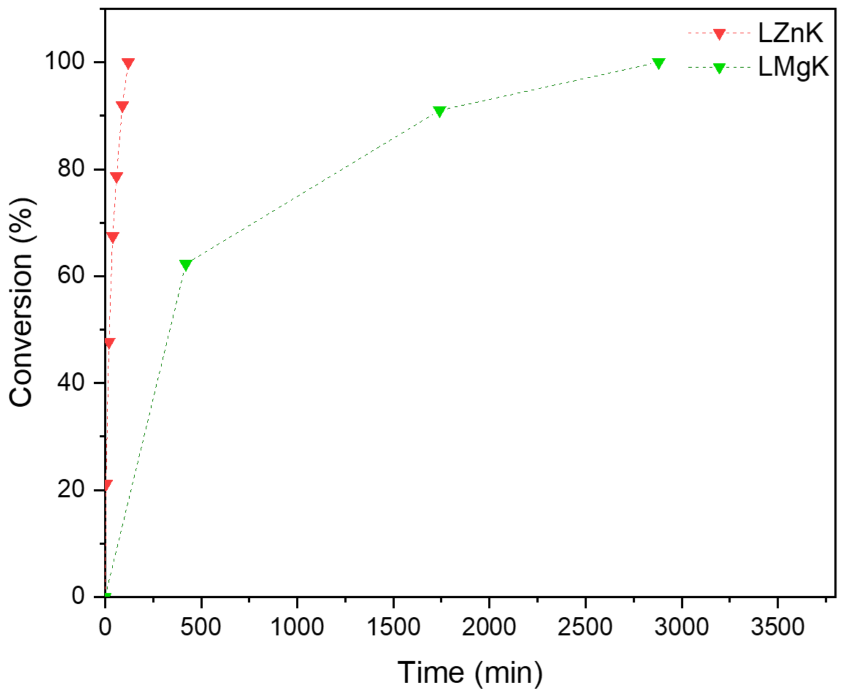

Figure S24 Conversion *vs* time for PET depolymerization catalyzed by **LZnK** or **LMgK** at 150 °C over a period of 48 hours.

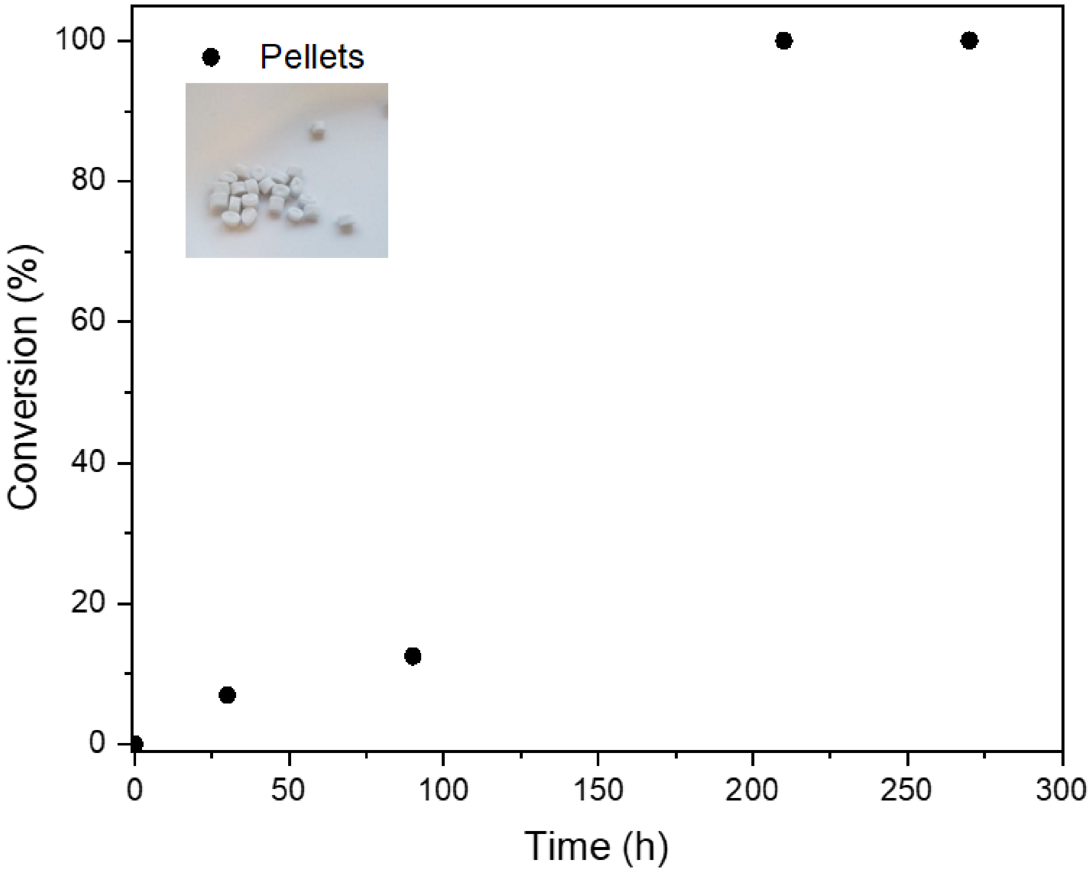

Figure S25. Conversion *vs* time for PET depolymerization catalyzed with **LMgK** using PET pellets.

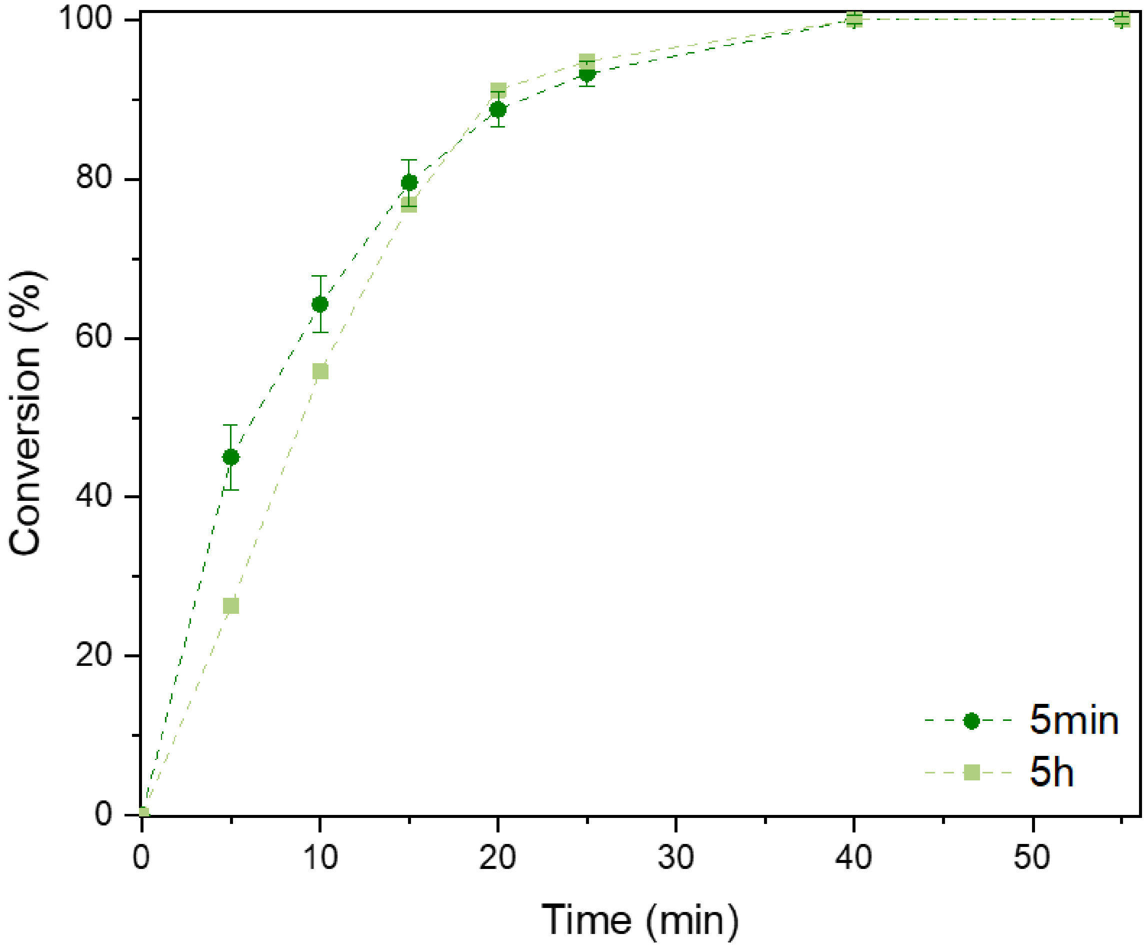

Figure S26. Conversion *vs* time of PET catalyzed by **LMgK** heating the system with catalyst, NMP, and ethylene glycol at 180 °C for 5 min and 5 h before PET addition and kinetics data point collection.

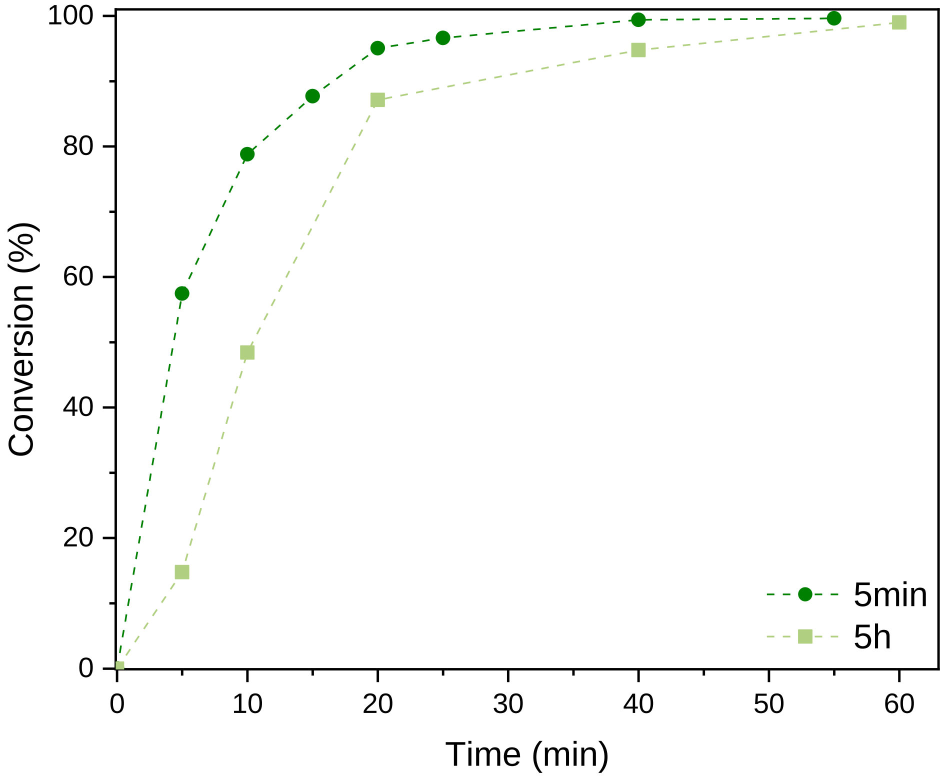

Figure S27. Conversion *vs* time of PET catalyzed by **LZnK** heating the system with catalyst, NMP, and ethylene glycol at 180 °C for 5 min and 5 h before PET addition and kinetics data point collection.

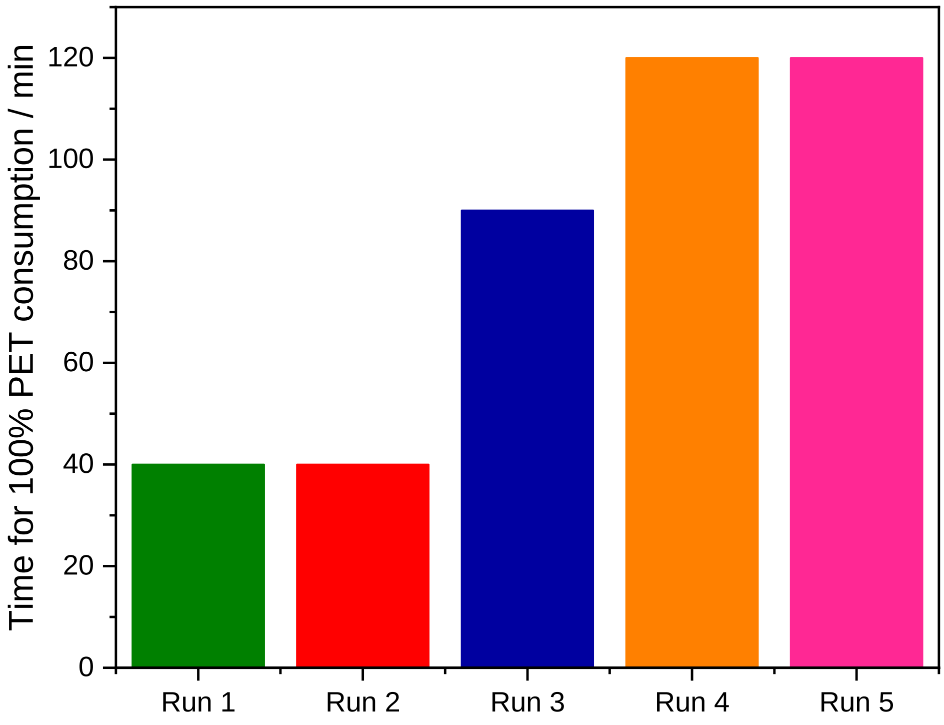

Figure S28. Depolymerization of successive additions of PET using **LZnK**. After complete depolymerization, the system was left to cool down to room temperature for different periods of time prior to restarting the reaction *via* heating to 180 °C for 5 min and adding a new batch of PET (Run 2: immediate addition; Run 3: 24 h; Run 4: 7 days and Run 5: 3 weeks).

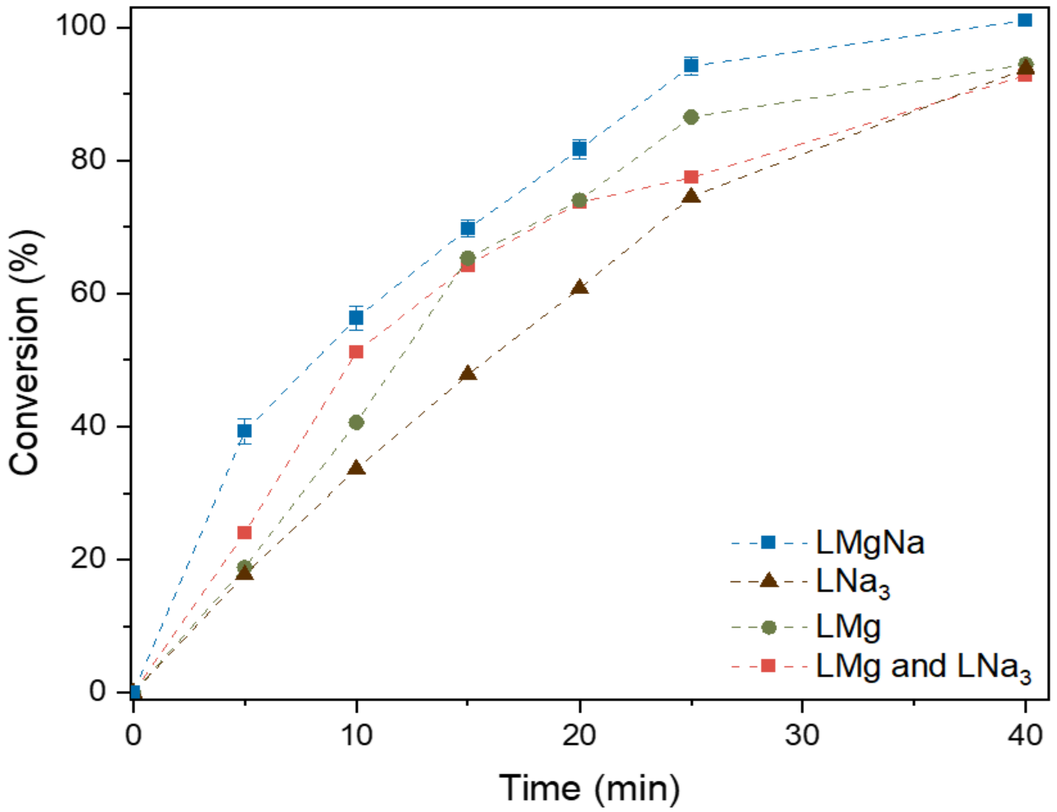

Figure S29. Comparison of the kinetics curves for the depolymerization of PET with ethylene glycol at 180 °C using catalysts **LMgNa**, **$L_2Mg_3·6H_2O$** (denoted as **LMg** in the key) and **LNa_3**.

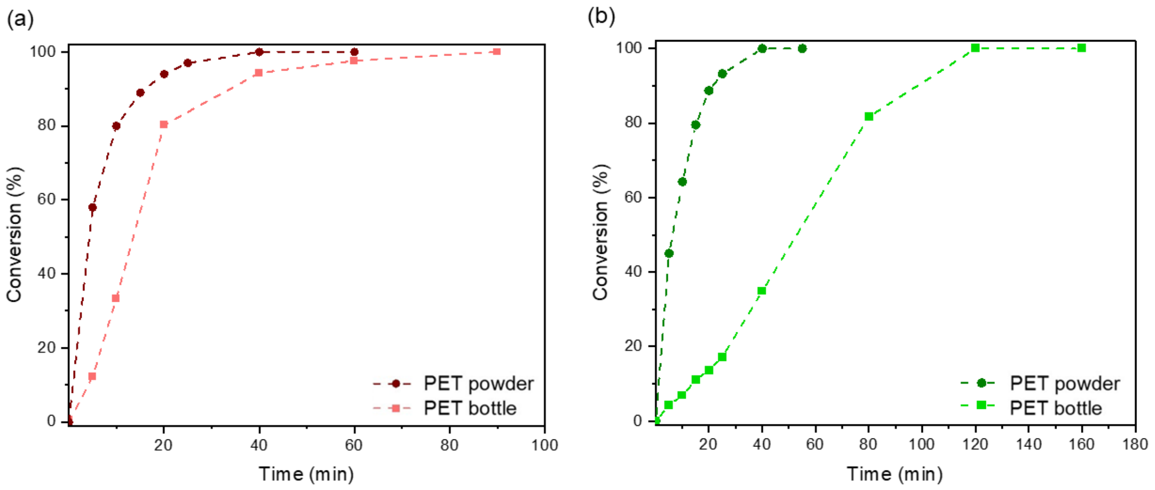

Figure S30. Conversion *vs* time for commercial PET bottle (5 mm^2) and PET powder, catalyzed by (a) **LZnK** and (b) **LMgK**

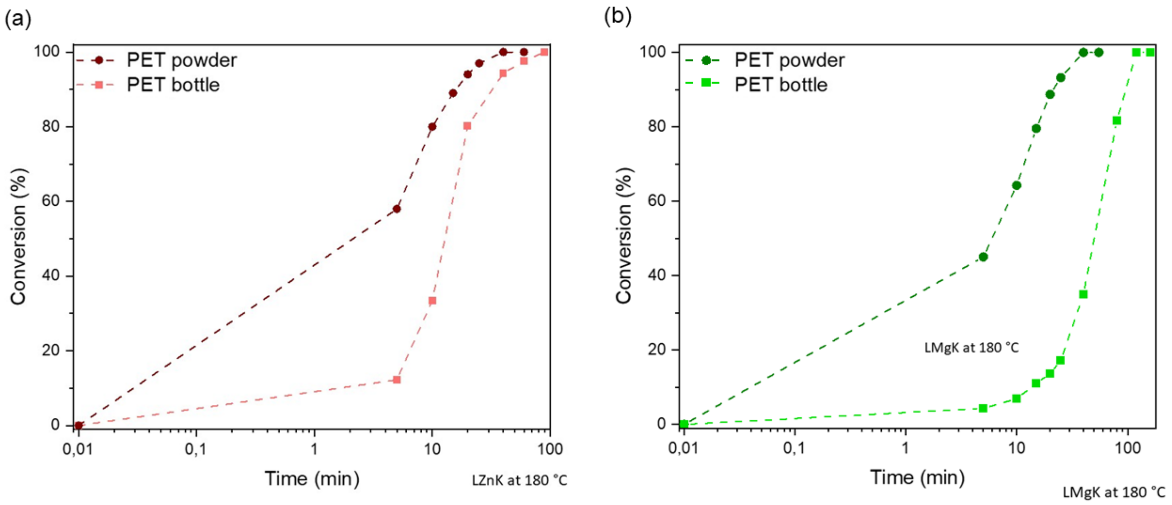

Figure S31. Conversion *vs* time (with a logarithmic x-axis) for commercial PET bottle (5 mm^2) and PET powder depolymerization, catalyzed by a) **LZnK** and b) **LMgK**.

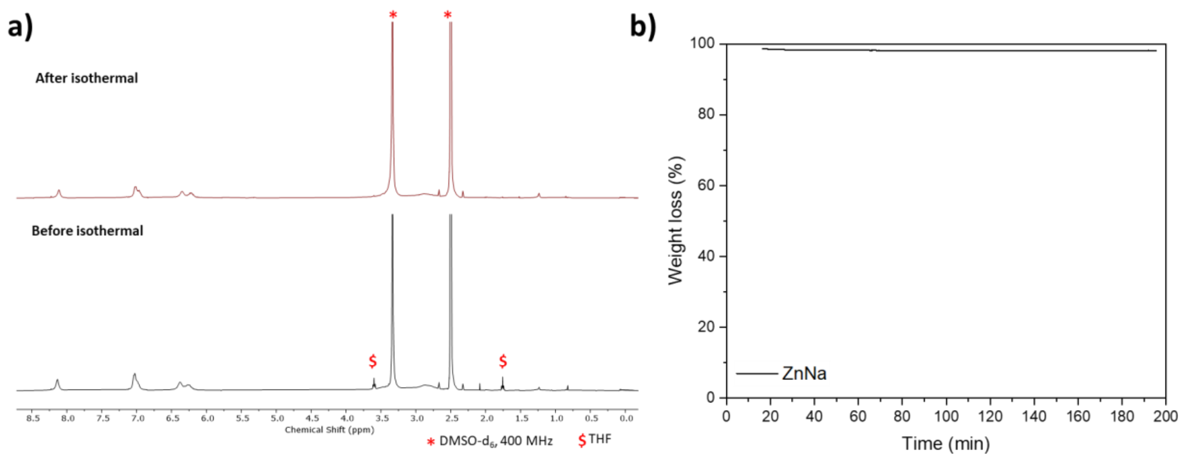

Figure S32. a) ^{1}H NMR spectra of complex **LZnNa** before and after heating at 180 °C for 3 h, (DMSO-d$_6$, 400 MHz, 298 K). b) TGA isothermal analysis of complex **LZnNa** at 180 °C for 3 h.

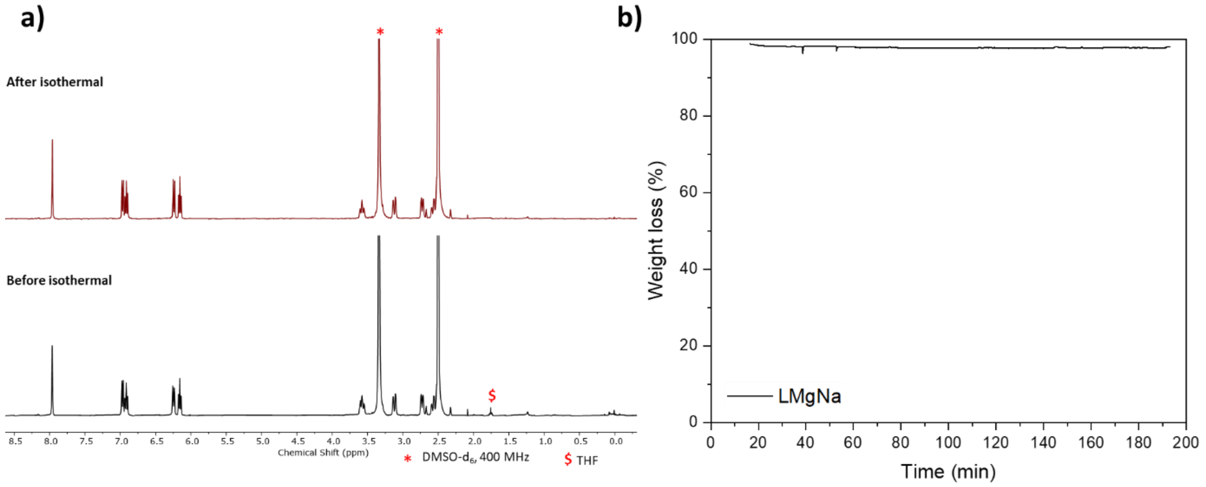

Figure S33. a) ^{1}H NMR spectra of complex **LMgNa** before and after heating at 180 °C for 3 h, (DMSO-d_6, 400 MHz, 298 K). b) TGA isothermal analysis of complex **LMgNa** at 180 °C for 3 h.

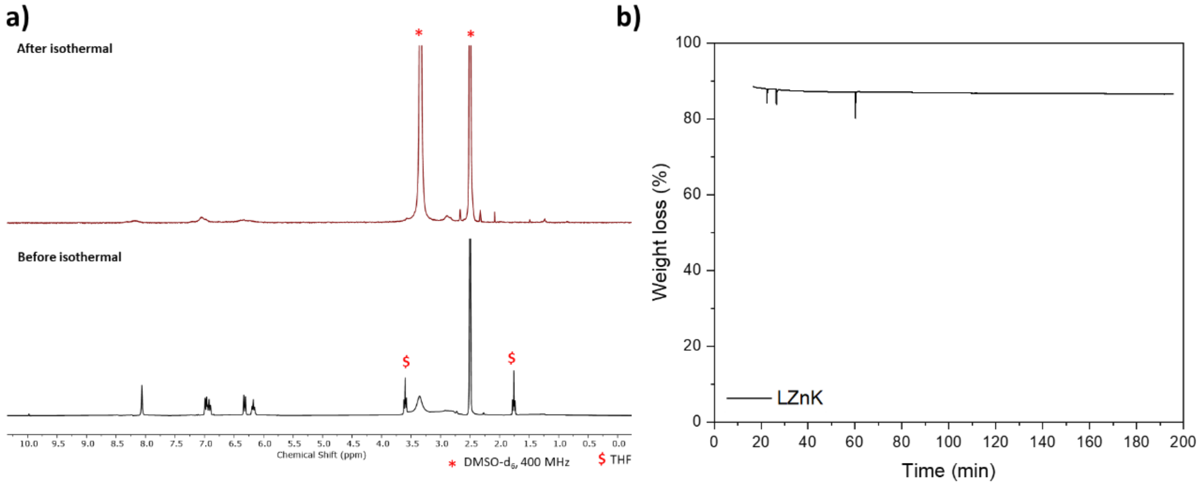

Figure S34. a) ^{1}H NMR spectra of complex **LZnK** before and after heating at 180 °C for 3 h (DMSO-d_6, 400 MHz, 298 K). b) TGA isothermal analysis of complex **LZnK** at 180 °C for 3 h.

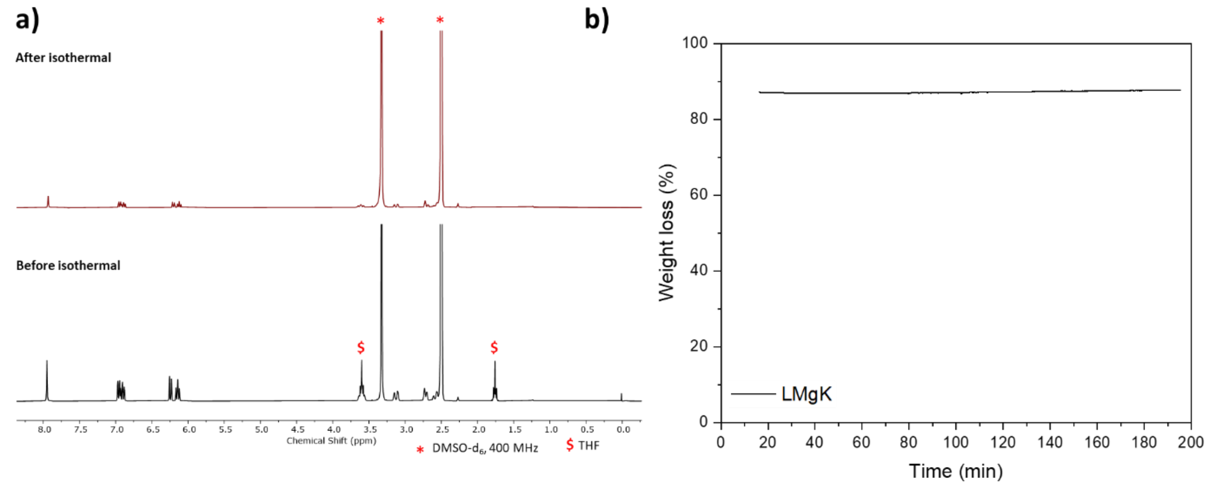

Figure S35. a) ^{1}H NMR spectra of complex **LMgK** before and after heating at 180 °C for 3 h (DMSO-d$_6$, 400 MHz, 298 K). b) TGA isothermal analysis of complex **LMgK** at 180 °C for 3 h.

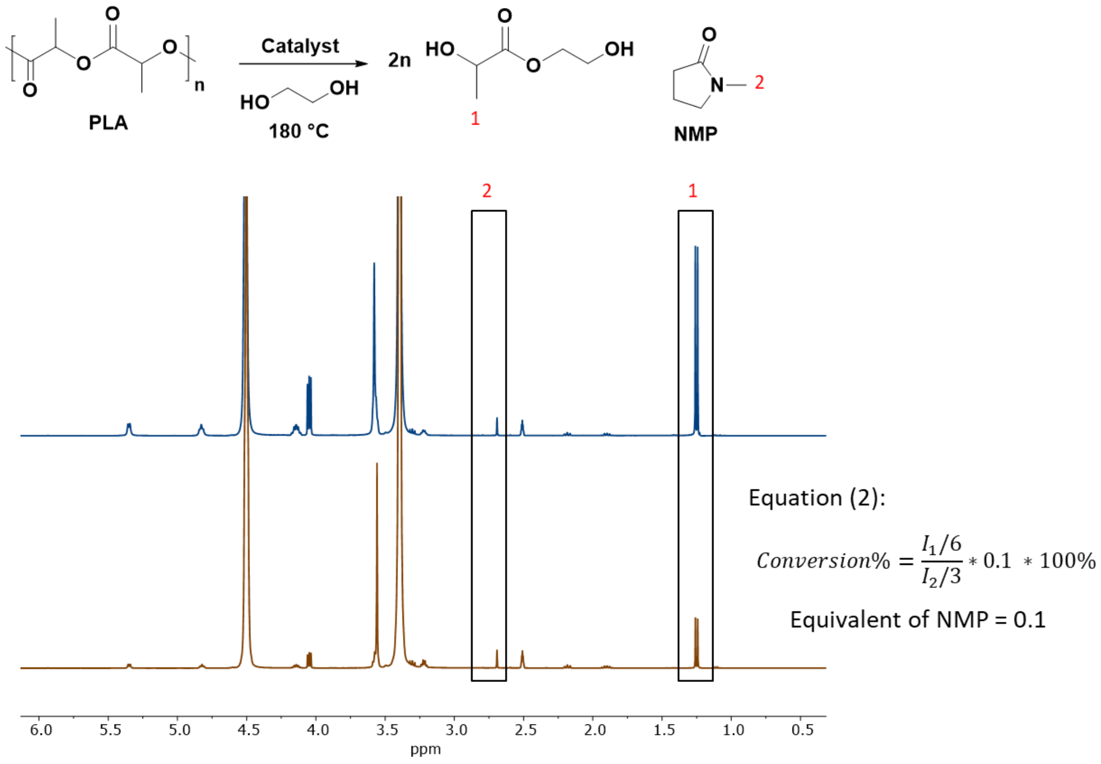

Figure S36. Overlaid ^{1}H NMR spectra (400 MHz, DMSO-d□) of PLA depolymerization catalyzed by **LZnK** in ethylene glycol at 180 °C. Spectra correspond to aliquots taken at 2 min (bottom) and 15 min (top). Conversion was determined from the integration ratio of the 2-HEtLa signal at δ = 1.25 ppm (d, 6H) relative to the internal standard NMP signal at δ = 2.71 ppm (s, 3H), using the formula given in Equation (2).

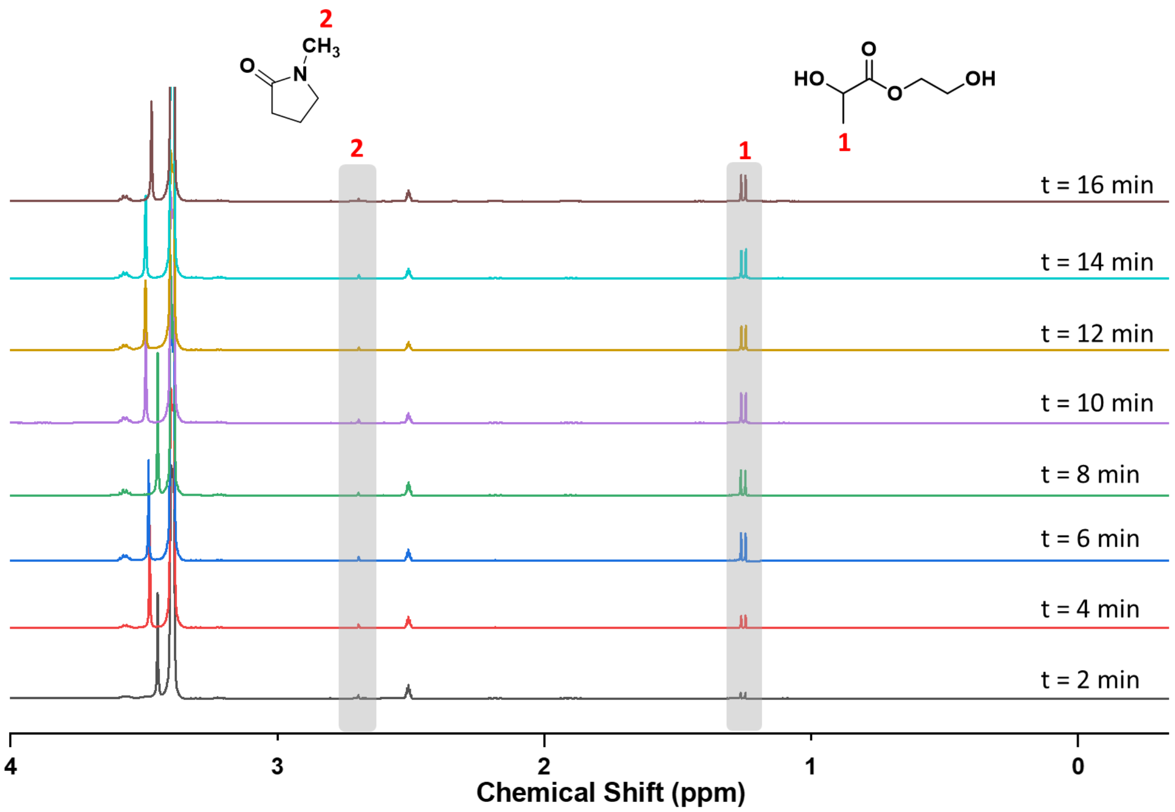

Figure S37. Overlaid ^{1}H NMR spectra of PLA depolymerization catalyzed by complex **LZnNa** in ethylene glycol at 180 °C over a 16 minute period.

Polymerization Data

Table S8 Ring-opening polymerisation of *rac*-LA with complexes **LZnNa**, **LZnK**, **LMgNa** and **LMgK** in the presence and absence of BnOH.[a]

Entry	[cat.]	Solvent	T / ˚C	Time/ min	%Conv.[b]	$M_{n,calc}$[c]/ g mol^{-1} (1 chain)	$M_{n,obs}$[d]/ g mol^{-1}	*Đ*[d]	P_i[e]
**1**	**LMgNa**	Toluene	60	180	79	11370	7320	2.31	0.57
**2[f]**	**LMgNa**	Toluene	60	1080	56	8030	7420	3.47	0.48
**3**	**LMgK**	Toluene	60	10	51	7380	6320	1.11	0.62
**4[f]**	**LMgK**	Toluene	60	10	4	550	-	-	-
**5**	**LZnNa**	THF	60	3	76	10960	11900	1.45	0.54
**6[f]**	**LZnNa**	THF	60	3	16	2300	7970	1.56	-
**7**	**LZnNa**	Toluene	120	5	98	14140	9800	1.58	0.51
**8[f]**	**LZnNa**	Toluene	120	6	72	10400	13970	1.40	0.46
**9**	**LZnK**	THF	60	1.5	86	12370	9920	1.47	0.57
**10[f]**	**LZnK**	THF	60	1.5	8	1190	-	-	-
**11**	**LZnK**	Toluene	60	5	93	13510	6950	1.97	0.63
**12[f]**	**LZnK**	Toluene	60	5	30	4260	9710	1.51	-

[a] Reaction conditions: Loading ratio of [cat.]:[BnOH]:[*rac*-LA] = 1:1(0):100, [*rac*-LA] = 1 M. All catalysts are considered as monomers. Reactions were duplicated. [b] Determined by ^{1}H NMR spectroscopy. [c] Calculated as ([*rac*-LA]/[catalyst])×(%conversion/100)×MW of *rac*-LA. [d] Determined by SEC analysis in THF solvent with universal calibration relative to polystyrene standards; M_n was calculated using a correction factor for M_n ($M_{n,obs}$ = 0.58×[M_n(SEC)]).[5] [e] Determined by homodecoupled ^{1}H NMR spectroscopy. [f] Reaction performed in the absence of BnOH.

Table S9. Ring-opening polymerisation of *rac*-LA with Zn-based complexes **LZnNa** and **LZnK** at 60 °C in toluene or THF using various time point to determine the reaction kinetics.[a]

Entry	[cat.]	Solvent	Time/ min	%Conv.[b]	$M_{n,calc}$[c]/ g mol^{-1} (1 chain)	$M_{n,calc}$[c]/ g mol^{-1} (2 chains)	$M_{n,obs}$[d]/ g mol^{-1}	$Đ$[d]	P_i[e]
1	LZnNa	THF	0.67	30	4260	2130	5710	1.41	-
2	LZnNa	THF	1	43	6170	3090	7080	1.38	-
3	LZnNa	THF	1.5	51	7310	3650	7960	1.40	-
4	LZnNa	THF	3	76	10960	5480	11900	1.45	0.54
5	LZnNa	toluene	0.67	35	4990	2490	4880	1.51	-
6	LZnNa	toluene	1	37	5340	2670	5230	1.62	0.48
7	LZnNa	toluene	1.5	47	6740	3370	6950	1.97	-
8	LZnNa	toluene	3	68	9860	4930	7660	1.63	0.47
9	LZnK	THF	0.25	55	7850	3930	10980	1.67	-
10	LZnK	THF	0.42	68	9720	4860	14230	1.58	-
11	LZnK	THF	1	80	11580	5790	11100	1.71	
12	LZnK	THF	1.5	86	12370	6180	9920	1.47	0.57
13	LZnK	toluene	0.67	43	6310	3160	7230	1.61	0.62
14	LZnK	toluene	1.5	68	9810	4910	8850	2.02	-
15	LZnK	toluene	3	81	11680	5840	8590	2.10	0.53
16	LZnK	toluene	5	93	13510	6750	6950	1.97	0.63

[a] Reaction conditions: Loading ratio of [cat.]:[BnOH]:[*rac*-LA] = 1:1:100, [*rac*-LA] = 1 M, 60 °C. All catalysts are considered as monomers. Reactions were duplicated. [b] Determined by ^{1}H NMR spectroscopy. [c] Calculated as ([*rac*-LA]/[catalyst])×(%conversion/100)×MW of *rac*-LA. [d] Determined by SEC analysis in THF solvent with universal calibration relative to polystyrene standards; M_n was calculated using correction factor for M_n ($M_{n,obs}$ = 0.58×[M_n(SEC)]).[5] [e] Determined by homodecoupled ^{1}H NMR spectroscopy.

Table S10. Ring-opening polymerisation of *rac*-LA with Mg-based complexes **LMgNa** and **LMgK** at 60 °C in toluene or THF using various time point to determine the reaction kinetics.[a]

Entry	[cat.]	Solvent	Time/ min	%Conv.[b]	$M_{n,calc}$[c]/ g mol^{-1} (1 chain)	$M_{n,calc}$[c]/ g mol^{-1} (2 chains)	$M_{n,obs}$[d]/ g mol^{-1}	$Đ$[d]	P_i[e]
**1**	**LMgNa**	THF	30	7	1070	530	-	-	-
**2**	**LMgNa**	THF	90	14	1990	990	-	-	-
**3**	**LMgNa**	THF	120	15	2200	1100	1370	1.13	-
**4**	**LMgNa**	THF	180	20	2690	1350	1890	1.10	0.63
**5**	**LMgNa**	THF	1080	67	9650	4820	4590	1.74	0.51
**6**	**LMgNa**	toluene	10	3	420	210	-	-	-
**7**	**LMgNa**	toluene	30	7	940	470	-	-	-
**8**	**LMgNa**	toluene	40	10	1430	710	-	-	-
**9**	**LMgNa**	toluene	50	12	1660	830	930	1.05	-
**10**	**LMgNa**	toluene	180	79	11370	5680	7320	2.31	0.57
**11**	**LMgK**	THF	15	4	550	280	-	-	-
**12**	**LMgK**	THF	30	6	820	410	-	-	-
**13**	**LMgK**	THF	60	8	1200	590	-	-	-
**14**	**LMgK**	THF	90	11	1500	770	1120	1.07	-
**15**	**LMgK**	THF	180	21	2970	1490	1130	1.11	-
**16**	**LMgK**	toluene	3	30	4330	2170	2930	1.16	-
**17**	**LMgK**	toluene	5	38	5460	2730	4800	1.11	0.62
**18**	**LMgK**	toluene	9	48	6940	3470	5380	1.10	-
**19**	**LMgK**	toluene	10	51	7380	3690	6320	1.11	0.62

[a] Reaction conditions: Loading ratio of [cat.]:[BnOH]:[*rac*-LA] = 1:1:100, [*rac*-LA] = 1 M, 60 °C. All catalysts are considered as monomers. Reactions were duplicated. [b] Determined by ^{1}H NMR spectroscopy. [c] Calculated as ([*rac*-LA]/[catalyst])×(%conversion/100)×MW of *rac*-LA. [d] Determined by SEC analysis in THF solvent with universal calibration relative to polystyrene standards; M_n was calculated using correction factor for M_n ($M_{n,obs}$ = 0.58×[M_n(SEC)]).[5] [e] Determined by homodecoupled ^{1}H NMR spectroscopy.

Table S11. Ring-opening polymerisation of *rac*-LA with **LZnNa**, **LZnK**, **LMgNa**, **LMgK**, $\mathbf{LH_2Na}$ and $\mathbf{LH_2K}$ at 60 °C in toluene or THF to benchmark heterometallic complexes against the homometallic alkali metal analogues.[a]

Entry	[cat.]	Solvent	Time/ min	%Conv.[b]	$M_{n,calc}$[c]/ g mol^{-1}	$M_{n,obs}$[d]/ g mol^{-1}	$Đ$[d]	P_i[e]
**1**	**LMgNa**	THF	180	20	2690	1890	1.10	0.63
**2**	**LMgK**	THF	15	4	550	-	-	-
**3**	**LZnNa**	THF	1.5	51	7310	7960	1.40	0.54
**4**	**LZnNa**	THF	3	76	10960	11900	1.45	0.54
**5**	**LZnK**	THF	1.5	86	12370	9920	1.47	0.57
**6**	$\mathbf{LH_2Na}$	THF	1.5	29	4110	7380	1.46	-
**7**	$\mathbf{LH_2K}$	THF	1	89	12800	4580	2.11	0.68
**8**	**LMgNa**	toluene	10	3	420	-	-	-
**9**	**LMgK**	toluene	5	38	5460	4800	1.11	0.62
**10**	**LZnNa**	toluene	1	37	5340	5230	1.62	0.48
**11**	**LZnK**	toluene	5	93	13510	6950	1.97	0.63
**12**	$\mathbf{LH_2Na}$	toluene	1	40	5780	7020	1.37	0.56
**13**	$\mathbf{LH_2K}$	toluene	5	76	10960	10180	2.41	0.47

[a] Reaction conditions: Loading ratio of [cat.]:[BnOH]:[*rac*-LA] = 1:1:100, [*rac*-LA] = 1 M, 60 °C. All catalysts are considered as monomers. Reactions were duplicated. [b] Determined by ^{1}H NMR spectroscopy. [c] Calculated as ([*rac*-LA]/[catalyst])×(%conversion/100)×MW of *rac*-LA. [d] Determined by SEC analysis in THF solvent with universal calibration relative to polystyrene standards; M_n was calculated using correction factor for M_n ($M_{n,obs}$ = 0.58×[M_n(SEC)]).[5] [e] Determined by homodecoupled ^{1}H NMR spectroscopy.

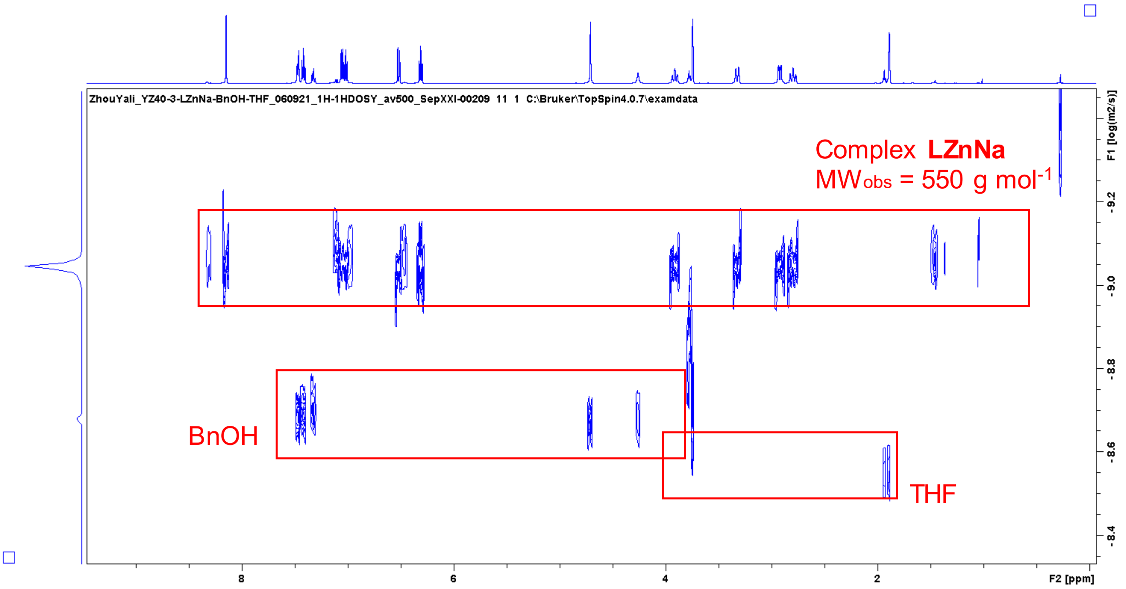

Figure S38. DOSY NMR spectrum (500 MHz) for the stoichiometric combination between complex **LZnNa** and BnOH (1:1) in THF-d_8 at 298 K.

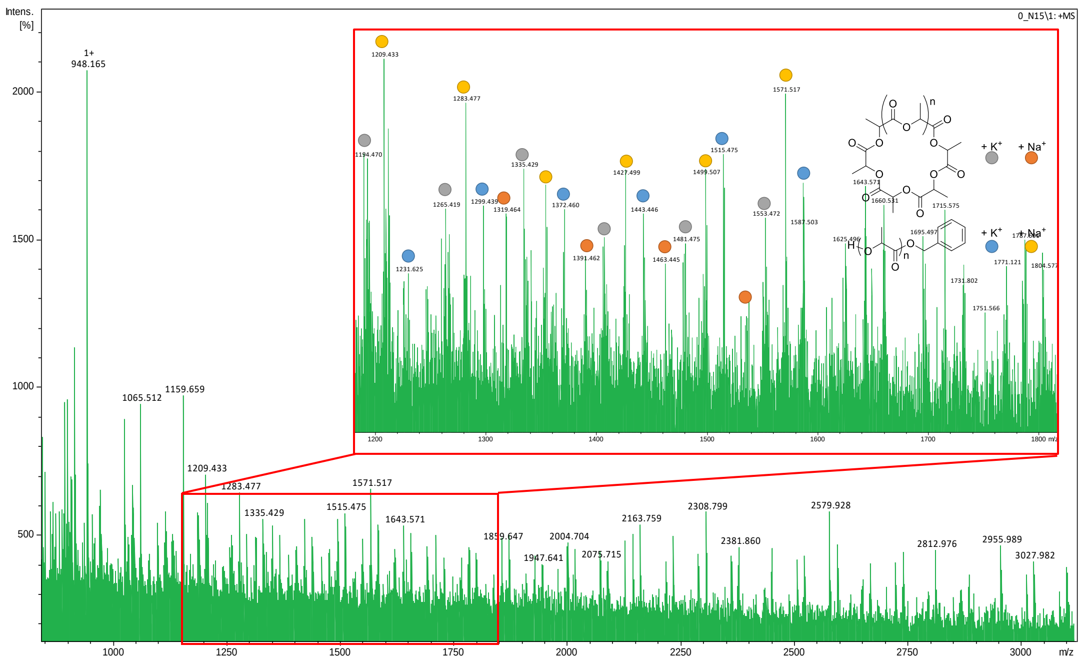

Figure S39. MALDI-ToF mass spectrum of PLA produced by **LZnNa** in the presence of BnOH in toluene at 60 °C showing the series of peaks corresponding to (i) linear BnO-PLA-H (blue $[M+K]^+$, yellow $[M+Na]^+$); and (ii) cyclic PLA (grey $[M+K]^+$, orange $[M+Na]^+$).

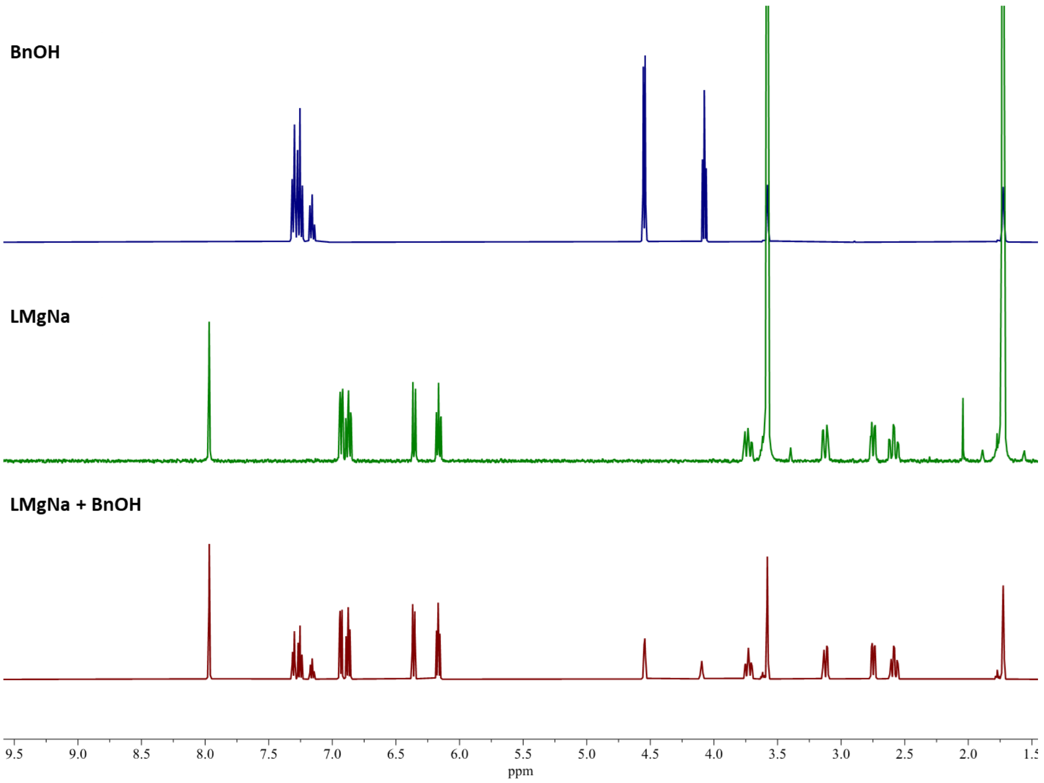

Figure S40. Overlaid ^1H NMR spectra (THF-d_8, 500 MHz) for the stoichiometric combination of complex **LMgNa** and BnOH at 298 K.

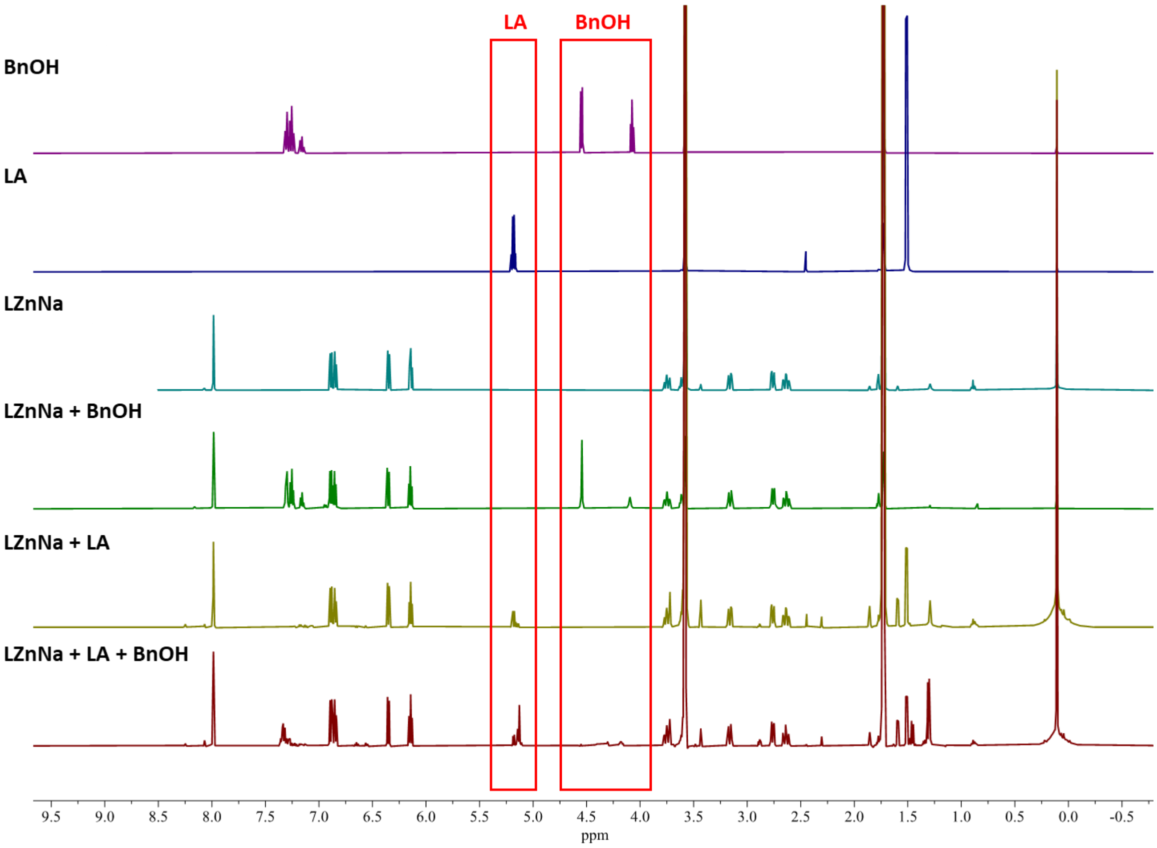

Figure S41. Overlaid ^{1}H NMR spectra (THF-d_8, 500 MHz) for the stoichiometric reaction of complex **LZnNa** and *rac*-LA in the presence or absence of BnOH at 298 K.

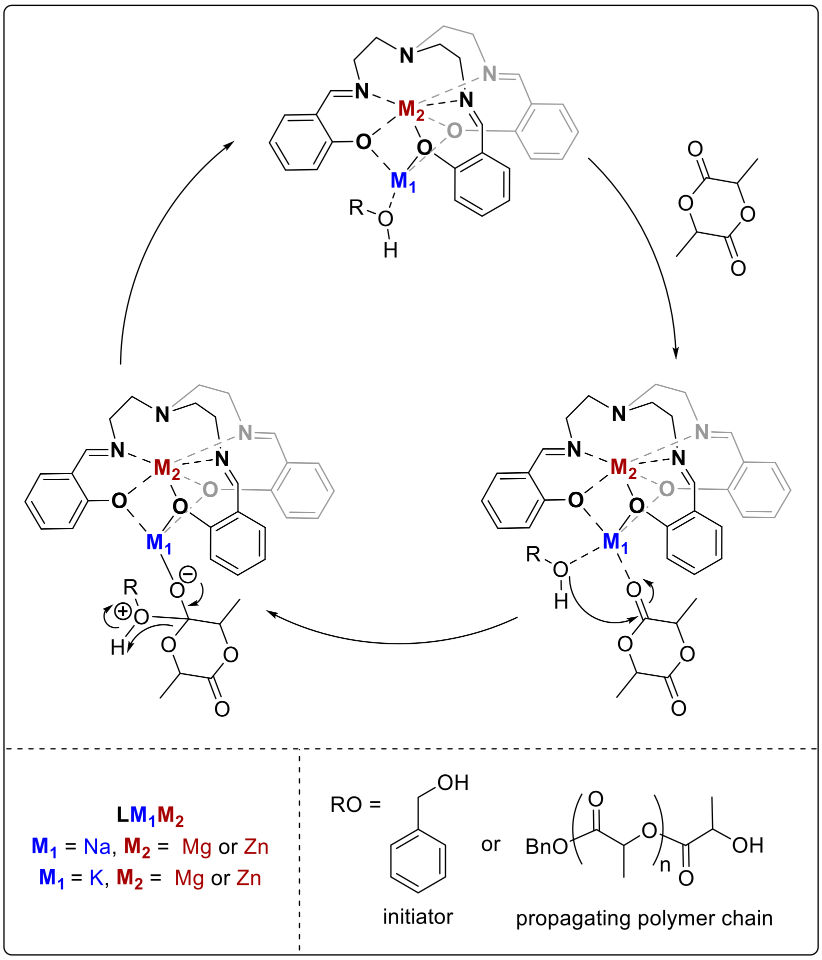

Figure S42. Proposed mechanism for the ring-opening polymerization of lactide by catalysts **LZnNa**, **LZnK**, **LMgNa** and **LMgK**, in the presence of 1 equiv. of benzyl alcohol (BnOH) as an exogeneous initiator.

References

(1) Zhou, Y.; Nichol, G. S.; Garden, J. A. Incorporating Sodium to Boost the Activity of Aluminium TrenSal Complexes towards Rac-Lactide Polymerisation. *Eur. J. Inorg. Chem.* **2022**, 2022 (16), e202200134.

(2) Tai, X.-S.; Zhao, W.-H. Synthesis, Spectral Characterization, and Luminescence Properties of a Cup-like Ligand and Its Magnesium(II) *Complex. Res. Chem. Intermed.* **2014**, 40 (5), 2075–2082.

(3) Sheldrick, G. M. SHELXT – Integrated Space-Group and Crystal-Structure Determination. *Acta Crystallogr. A Found. Adv.* **2015**, 71 (1), 3–8.

(4) Sheldrick, G. M. Crystal Structure Refinement with SHELXL. *Acta Crystallogr. C Struct. Chem.* **2015**, 71 (1), 3–8.

(5) Kowalski, A.; Duda, A.; Penczek, S. Polymerization of L,L-Lactide Initiated by Aluminum Isopropoxide Trimer or Tetramer. *Macromolecules* **1998**, 31 (7), 2114–2122.

(6) Spicer, A. J.; Brandolese, A.; Dove, A. P. Selective and Sequential Catalytic Chemical Depolymerization and Upcycling of Mixed Plastics. *ACS Macro. Lett.* **2024**, 13(2), 189–194.

(7) Gayathri, V.; Sheyara, R. T. B.; Devassy, N.; Samanta, D. Investigating the Degradation of PET Utilizing NHC-Based Catalysts and Effective Reuse of the Degradation Product as an Additive with Polyurethane Adhesive Material. *J. Appl. Polym. Sci.* **2022**, 139 (27), e52474.

(8) Tufano, F.; Santulli, F.; Liguori, C.; Santoriello, G.; Ritacco, I.; Caporaso, L.; Grisi, F.; Mazzeo, M.; Lamberti, M. Versatile NHC-Based Zinc and Magnesium Complexes for the Synthesis and Chemical Recycling of Aliphatic Polyesters and Polycarbonates. *Catal. Sci. Technol.* **2025**, 15 (3), 822–835.

(9) Fuentes, J. A.; Smith, S. M.; Scharbert, M. T.; Carpenter, I.; Cordes, D. B.; Slawin, A. M. Z.; Clarke, M. L. On the Functional Group Tolerance of Ester Hydrogenation and Polyester Depolymerisation Catalysed by Ruthenium Complexes of Tridentate Aminophosphine Ligands. *Chem.-Eur. J.* **2015**, 21 (30), 10851–10860.

(10) Westhues, S.; Idel, J.; Klankermayer, J. Molecular Catalyst Systems as Key Enablers for Tailored Polyesters and Polycarbonate Recycling Concepts. *Sci. Adv.* **2018**, 4 (8), eaat9669.

(11) Hermann, A.; Hill, S.; Metz, A.; Heck, J.; Hoffmann, A.; Hartmann, L.; Herres-Pawlis, S. Next Generation of Zinc Bisguanidine Polymerization Catalysts towards Highly Crystalline, Biodegradable Polyesters. *Angew. Chem. Int. Ed.* **2020**, 59 (48), 21778–21784.

(12) Fuchs, M.; Schäfer, P. M.; Wagner, W.; Krumm, I.; Walbeck, M.; Dietrich, R.; Hoffmann, A.; Herres-Pawlis, S. A Multitool for Circular Economy: Fast Ring-Opening Polymerization and Chemical Recycling of (Bio)Polyesters Using a Single Aliphatic Guanidine Carboxy Zinc Catalyst. *ChemSusChem* **2023**, 16 (12), e202300192.

(13) Conrads, C.; Burkart, L.; Soerensen, S.; Noichl, S.; Kara, Y.; Heck, J.; Hoffmann, A.; Herres-Pawlis, S. Understanding Structure–Activity Relationships: Iron(II) Complexes of "Legacy Guanidines" as Catalysts for the Synthesis of Polylactide. *Catal. Sci. Technol.* **2023**, 13 (20), 6006–6021.

(14) Krall, E. M.; Klein, T. W.; Andersen, R. J.; Reader, D. S.; Dauphinais, B. C.; McIlrath, S. P.; Fischer, Anne A.; Carney, M. J.; Robertson, N. J. Controlled Hydrogenative Depolymerization of Polyesters and Polycarbonates Catalyzed by Ruthenium(II) PNN Pincer Complexes. *Chem. Commun.* **2014**, 50 (38), 4884–4887.

(15) Monsigny, L.; Berthet, J. C.; Cantat, T. Depolymerization of Waste Plastics to Monomers and Chemicals Using a Hydrosilylation Strategy Facilitated by Brookhart's Iridium(III) Catalyst. *ACS Sustain. Chem. Eng.* **2018**, 6 (8), 10481–10488.

(16) Hu, Y.; Zhang, S.; Xu, J.; Liu, Y.; Yu, A.; Qian, J.; Xie, Y. Highly Efficient Depolymerization of Waste Polyesters Enabled by Transesterification/Hydrogenation Relay Under Mild Conditions. *Angew. Chem. Int. Ed.* **2023**, 62 (45), e202312564.

(17) Román-Ramírez, L. A.; McKeown, P.; Jones, M. D.; Wood, J. Poly(Lactic Acid) Degradation into Methyl Lactate Catalyzed by a Well-Defined Zn(II) Complex. *ACS Catal.* **2019**, 9 (1), 409–416.

(18) Payne, J. M.; Kociok-Köhn, G.; Emanuelsson, E. A. C.; Jones, M. D. Zn(II)- And Mg(II)-Complexes of a Tridentate {ONN} Ligand: Application to Poly(Lactic Acid) Production and Chemical Upcycling of Polyesters. *Macromolecules* **2021**, 54 (18), 8453–8469.

(19) Stewart, J. A.; Powell, L. T. W.; Cullen, M. J.; Kociok-Köhn, G.; Davidson, M. G.; Jones, M. D. Imino-Pyrrole Zn(II) Complexes for the Rapid and Selective Chemical Recycling of Commodity Polymers. *Angew. Chem. Int. Ed.* **2025**, 64 (22), e202502845.

(20) Zhang, S.; Hu, Q.; Zhang, Y. X.; Guo, H.; Wu, Y.; Sun, M.; Zhu, X.; Zhang, J.; Gong, S.; Liu, P.; Niu, Z. Depolymerization of Polyesters by a Binuclear Catalyst for Plastic Recycling. *Nat. Sustain.* **2023**, 6 (8), 965–973.

(21) Fliedel, C.; Vila-Viçosa, D.; Calhorda, M. J.; Dagorne, S.; Avilés, T. Dinuclear Zinc-N-Heterocyclic Carbene Complexes for Either the Controlled Ring-Opening Polymerization of Lactide or the Controlled Degradation of Polylactide under Mild Conditions. *ChemCatChem* **2014**, 6 (5), 1357–1367.

(22) Payne, J.; McKeown, P.; Driscoll, O.; Kociok-Köhn, G.; Emanuelsson, E. A. C.; Jones, M. D. Make or Break: Mg(II)- and Zn(II)-Catalen Complexes for PLA Production and Recycling of Commodity Polyesters. *Polym. Chem.* **2021**, 12 (8), 1086–1096.

(23) Shi, Z.; Jiang, H.; Xue, C.; Yang, Y.; Hou, Z.; Wang, H. Metal Synergistic Dual Activation Enables Efficient Transesterification by Multinuclear Titanium Catalyst: Recycling and Upcycling of Polyester Waste. *Angew. Chem.* **2025**, 137 (26), e202505024.

(24) Payne, J.; McKeown, P.; Mahon, M. F.; Emanuelsson, E. A. C.; Jones, M. D. Mono- and Dimeric Zinc(Ii) Complexes for PLA Production and Degradation into Methyl Lactate – a Chemical Recycling Method. *Polym. Chem.* **2020**, 11 (13), 2381–2389.

(25) Zhang, S.; Xue, Y.; Wu, Y.; Zhang, Y. X.; Tan, T.; Niu, Z. PET Recycling under Mild Conditions via Substituent-Modulated Intramolecular Hydrolysis. *Chem. Sci.* **2023**, 14 (24), 6558–6563.

(26) Lin, J.; Wang, P.; Fan, L.; Xiao, T.; Cheng, J.; Chen, X. Quinolyl/Pyridyl-Amino Li Complexes as Dual Catalysts for the Ring-Opening Polymerization of Cyclic Esters and Degradation toward a Circular Economy Approach. *Polymer* **2023**, 285, 126352.

(27) Li, M.; Zhang, S. Tandem Chemical Depolymerization and Photoreforming of Waste PET Plastic to High-Value-Added Chemicals. *ACS Catal*. **2024**, 14 (5), 2949–2958.

(28) Sarazin, Y.; Howard, R. H.; Hughes, D. L.; Humphrey, S. M.; Bochmann, M. Titanium, Zinc and Alkaline-Earth Metal Complexes Supported by Bulky O,N,N,O-Multidentate Ligands: Syntheses, Characterisation and Activity in Cyclic Ester Polymerisation. *Dalton Trans.* **2006**, 60 (2), 340–350.

(29) Payne, J. M.; Kamran, M.; Davidson, M. G.; Jones, M. D. Versatile Chemical Recycling Strategies: Value-Added Chemicals from Polyester and Polycarbonate Waste. *ChemSusChem* **2022**, 15 (8), e202200255.

(30) McKeown, P.; Román-Ramírez, L. A.; Bates, S.; Wood, J.; Jones, M. D. Zinc Complexes for PLA Formation and Chemical Recycling: Towards a Circular Economy. *ChemSusChem* **2019**, 12 (24), 5233–5238.
